# Coordinatively Unsaturated Metallates of Cobalt(II), Nickel(II), and Zinc(II) Guarded by a Rigid and Narrow Void

**DOI:** 10.1021/acs.inorgchem.3c01335

**Published:** 2023-07-18

**Authors:** Christopher D. Hastings, Lucy S. X. Huffman, Chandan Kumar Tiwari, Jolaine Galindo Betancourth, William W. Brennessel, Brandon R. Barnett

**Affiliations:** Department of Chemistry, University of Rochester, Rochester, New York 14627, United States

## Abstract

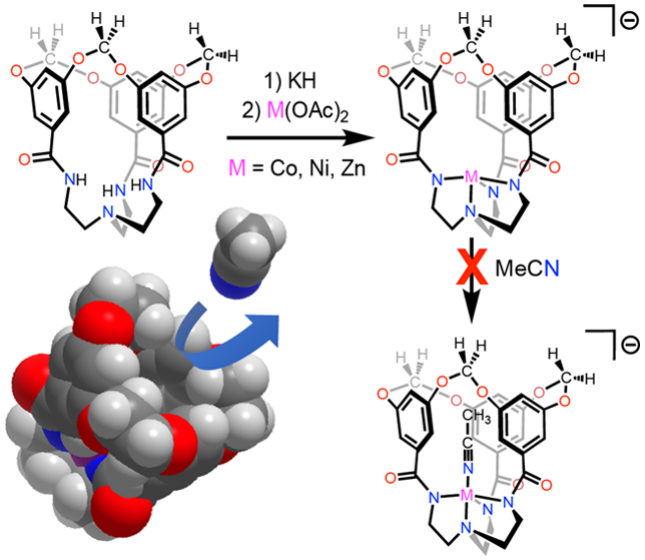

Both natural enzymatic systems and synthetic porous material catalysts utilize well-defined and uniform channels to dictate reaction selectivities on the basis of size or shape. Mimicry of this design element in homogeneous systems is generally difficult owing to the flexibility inherent in most small molecular species. Herein, we report the synthesis of a tripodal ligand scaffold that orients a narrow and rigid cavity atop accessible metal coordination space. The permanent void is formed through a macrocyclization reaction whereby the 3,5-dihydroxyphenyl arms are covalently linked through methylene bridges. Deprotonative metallation leads to anionic and coordinatively unsaturated complexes of divalent cobalt, nickel, and zinc. An analogous series of trigonal monopyramidal complexes bearing a nonmacrocyclized variant of the tripodal ligand are also reported. Physical characterization of the coordination complexes has been carried out using multiple spectroscopic techniques (NMR, EPR, and UV–vis), cyclic voltammetry, and X-ray diffraction. Complexes of the macrocyclized [L^OCH2O^]^3–^ ligand retain a rigid cavity upon metallation, with this cavity guarding the entrance to the open axial coordination site. Through a combination of spectroscopic and computational studies, it is shown that acetonitrile entry into the void is sterically precluded, disrupting anticipated coordination at the intracavity site.

## Abstract 2

A new tripodal ligand system is used to access unsaturated metallates of cobalt(II), nickel(II), and zinc(II). A narrow cavity built into the ligand through a macrocyclization reaction is shown to gate access to the open metal coordination site.

## Introduction

Synthetic inorganic chemistry has witnessed an increasing focus on the secondary coordination sphere as a means to control reaction outcomes at ligated metal centers.^1−5^ Specific efforts are often guided by the finely tuned microenvironments designed by nature to govern substrate binding and product formation at metallocofactors.^3,6^ Indeed, carefully tailored ligands incorporating H-bond donors/acceptors,^7−15^ Lewis acids,^16−21^ or electrostatic charges^22−25^ are now often used to stabilize transition states or products as a means to alter reaction outcomes of metal complexes with small molecules.

In order to regulate the arrival of exogenous species and control substrate conformation, nature frequently buries metalloenzymes within protein structures, with access for exogenous substrates available only through well-defined channels. Mimicry of this property in synthetic complexes can be approached by housing metal-binding chelators within channels defined by macrocyclic motifs.^26−29^ Provided that the channel serves as the sole point of ingress for substrates, metal-based reactivity becomes dependent on the kinetic feasibility of passage through the channel. Similar considerations guard reactivity within crystalline porous materials, as substrates must diffuse into a crystallite to access non-surface active sites.^30,31^ Industrial zeolite catalysis often exploits this approach on enormous scales to realize selectivities on the basis of size/shape.^32^ In principle, such a design strategy could effect interesting selectivities in synthetic homogeneous catalytic schemes, although realization has often proven elusive using known systems.^33^

**Figure 1:**
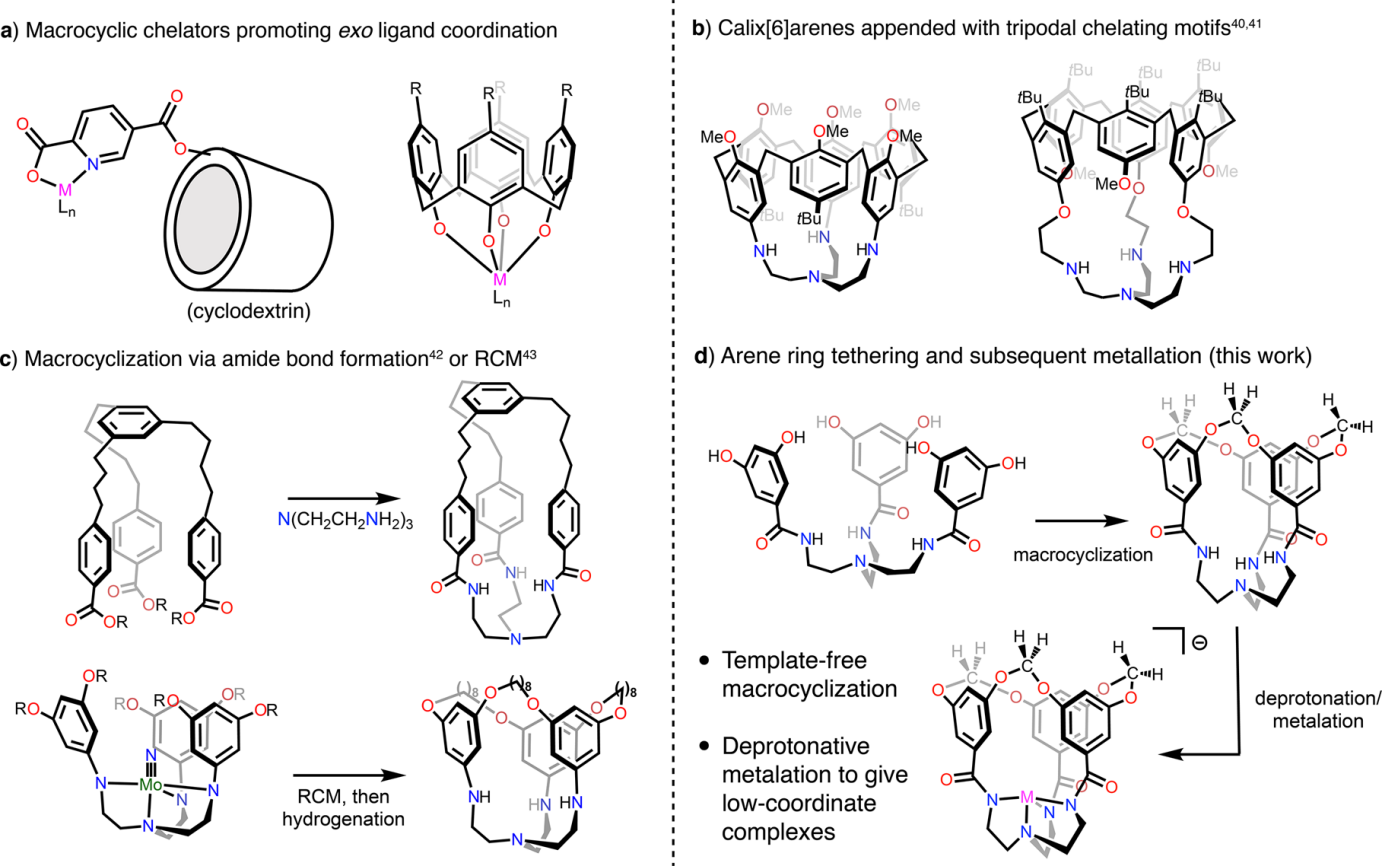
Examples of synthetic cavity-bearing ligand systems.

Pioneering work from Breslow demonstrated that cyclodextrins appended with chelating motifs could localize a hydrophobic channel adjacent to a metal center.^26,34^ Subsequent approaches in ligand design have expanded the diversity of macrocyclic systems–most frequently employing calixarenes and resorcinarenes–and have focused on spatially controlling the macrocycle’s orientation with respect to the metal center (Figure 1a).^35−41^ Macrocycles bearing judiciously designed chelating groups can result in the macrocycle interior serving as the sole access point for the enshrouded metal center (Figure 1b). However, synthetic routes to these systems can be laborious as the macrocycle must be synthesized, chemically modified, and then covalently linked to the metal binding motif. As an alternative approach, the groups of Lu^42^ and Schrock^43^ have demonstrated macrocyclization reactions upon tris(2-aminoethyl)amine (TREN)-based scaffolds to orient either a 45-membered ring or an arene-capped organic cavity above the metal binding pocket (Figure 1c). These procedures obviate the separate synthesis of a macrocyclic species that is subsequently decorated with chelating motifs and theoretically could enable the construction of a library of proligands with varying cavity dimensions, although no follow-up reports have appeared for either system.

We endeavored to develop a short and modular synthetic route to a ligand that orients a narrow and rigid channel above an accessible metal coordination site. Starting with a TREN-derived tris-amide platform bearing 3,5-dihydroxyphenyl arms, we demonstrate the construction of a rigid and narrow macrocycle-defined channel that is oriented directly atop the metal-binding pocket (Figure 1d). This three-step procedure employs macrocyclization as the final step and evokes the rigidification of resorcinarenes to form cavitands by linking adjacent phenol groups through a methylene bridge.^44,45^ Deprotonation and metallation with cobalt(II), nickel(II), and zinc(II) sources leads to anionic four-coordinate complexes containing an open coordination site that is housed inside the rigid cavity. An additional series of complexes bearing a ligand variant that lacks a rigid cavity is also presented for comparison. It is shown that the narrow channel profile in the macrocyclized ligand precludes acetonitrile binding to the open site in the cobalt(II) congener, while nitrile coordination readily occurs in the nonmacrocyclized cobalt(II) complex. Computational investigations reveal that intracavity acetonitrile binding is precluded by the narrow dimensions of the void.

## Results and Discussion

**Figure 2:**
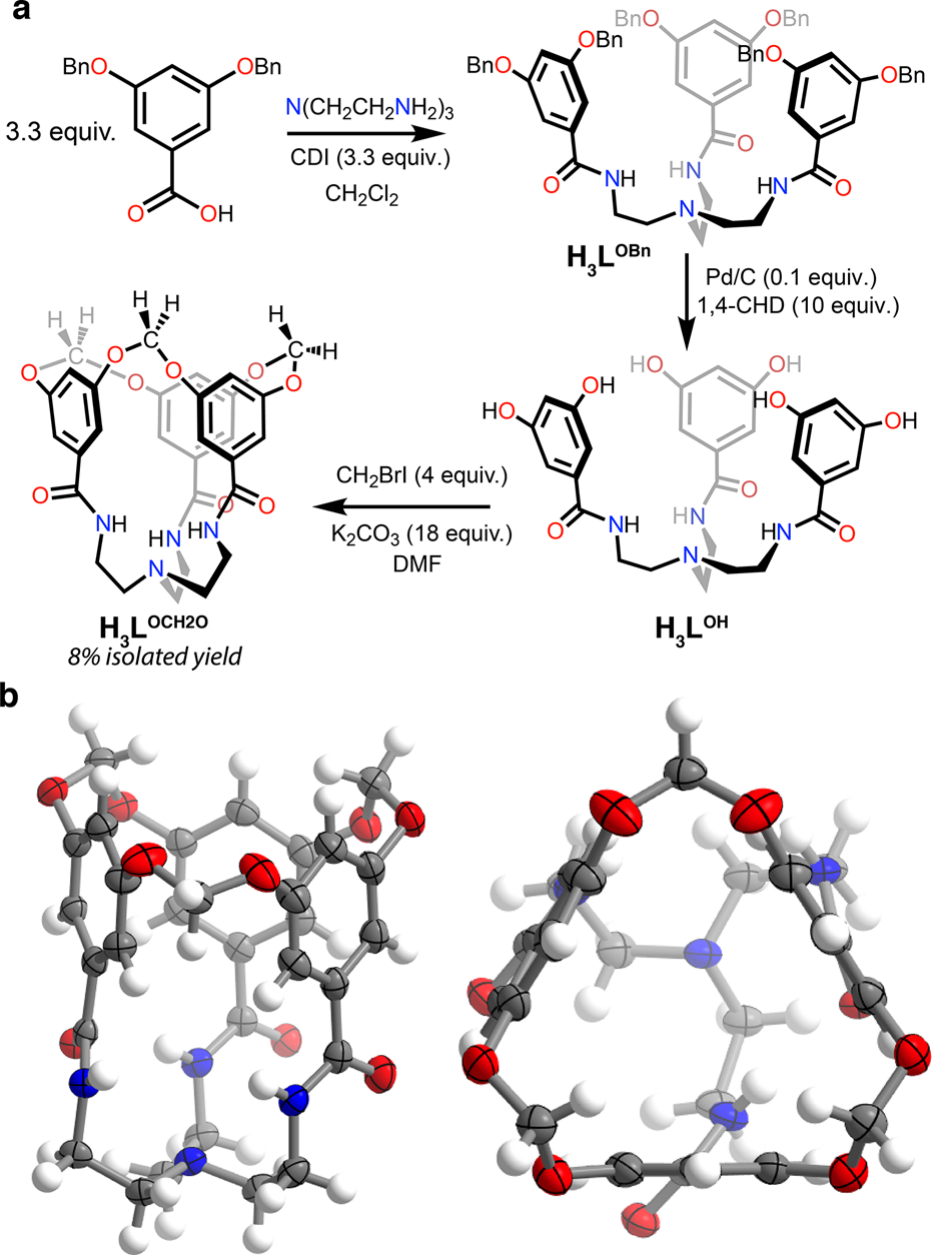
(a) Synthesis and (b) two views of the solid-state structure of **H**_**3**_**L**^**OCH2O**^. CDI is an abbreviation for 1,1′-carbonyldiimidazole, while 1,4-CHD denotes 1,4-cyclohexadiene. Thermal ellipsoids are shown at the 50% level.

A tripodal tris-amide bearing 3,5-dihydroxyphenyl arms was targeted as a precursor to a macrocyclized proligand (Figure 2a). Benzyl protection of the phenolic groups in 3,5-dihydroxybenzoic acid allowed for smooth amide bond formation with TREN using 1,1′-carbonyldiimidazole (CDI) as a coupling mediator.^46^ Deprotection of **H**_**3**_**L**^**OBn**^ with 1,4-cyclohexadiene (1,4-CHD) and catalytic Pd/C resulted in the formation of the desired **H**_**3**_**L**^**OH**^ as a hygroscopic colorless solid. Taking inspiration from the rigidification of resorcinarenes to form cavitands,^44,45^ it was endeavored to covalently tether adjacent aromatic rings to one another through methylene bridges. A variety of dihalomethane reagents (CH_2_BrCl, CH_2_Br_2_, CH_2_BrI, and CH_2_I_2_) were found capable of producing the desired macrocyclized species **H**_**3**_**L**^**OCH2O**^ upon heating with **H**_**3**_**L**^**OH**^ in the presence of a Brønsted base, as assayed by ^1^H NMR spectroscopy and electrospray ionization mass spectrometry (Figure 2). It should be noted that macrocyclization must compete with oligomerization processes which link multiple tris-amide species together through a methylene unit.^47^

Given that organic cavitand formation typically employs carbonate bases and *N,N*-dimethylformamide (DMF) as the solvent,^48,49^ our optimization efforts surveyed a library of carbonate salts (Li_2_CO_3_, Na_2_CO_3_, K_2_CO_3_, Cs_2_CO_3_, Ag_2_CO_3_, and (NH_4_)_2_CO_3_) while also varying the dihalomethane reagent (see above) and its rate of addition, reaction temperature, and dilution. It was found that the slow addition of CH_2_BrI to **H**_**3**_**L**^**OH**^ (12 mM) and K_2_CO_3_ in DMF at room temperature, with subsequent heating to 60 °C, produces **H**_**3**_**L**^**OCH2O**^ in the highest consistent yields (Figure 2). By the removal of aliquots of known volume from reaction mixtures and analysis by ^1^H NMR with an internal standard, it was determined that **H**_**3**_**L**^**OCH2O**^ was produced in approximately 10% yield. Isolation of macrocyclized **H**_**3**_**L**^**OCH2O**^ from these crude products is accomplished via Soxhlet extraction with CHCl_3_. Serial triturations of the obtained extractant with water, methanol, and hexanes yields spectroscopically pure **H**_**3**_**L**^**OCH2O**^ as a colorless solid in average isolated yields of approximately 8%.

Single crystals of **H**_**3**_**L**^**OCH2O**^ were obtained from a dilute methanol solution. Solid-state structure determination by X-ray diffraction confirms the formation of an 18-membered ring that defines the crest of a narrow cavity, which is bound by the faces of three aromatic rings (Figure 2b). The methylene bridges give rise to a pair of doublets in the ^1^H NMR spectrum that correspond to diastereotopic H atoms bearing an *endo* or *exo* relationship to the cavity (Figure S5). Although the solid-state structure depicts two distinct methylene orientations (two point upward atop the crest while the third is rotated downward), these bridges hinge rapidly and display equivalence by ^1^H NMR spectroscopy at temperatures of as low as −80 °C (Figure S15). The covalent linkages defining the macrocycle endow **H**_**3**_**L**^**OCH2O**^ with excellent thermal stability, as heating to 100 °C in dry DMSO-*d*_6_ for 1 week leaves **H**_**3**_**L**^**OCH2O**^ unchanged. Notably, exposing **H**_**3**_**L**^**OCH2O**^ to wet DMSO-*d*_6_ at this temperature results in substantial decomposition over the course of several days (Figure S16), potentially due to the hydrolysis of the acetal linkages.

**Figure 3:**
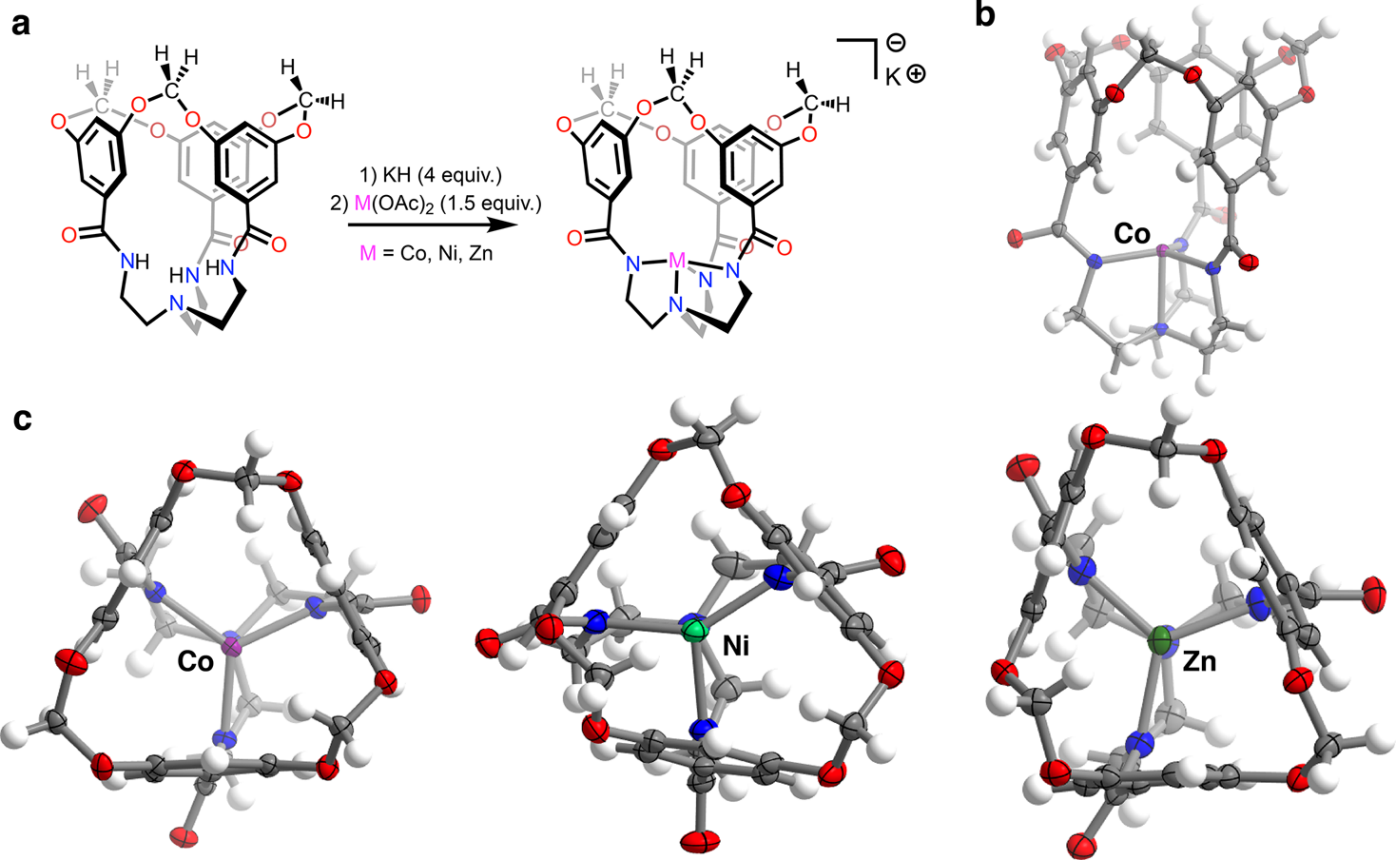
(a) Synthesis of the metallates **K[ML**^**OCH2O**^**]** (M = Co, Ni, Zn). (b) Solid-state structure of **[CoL**^**OCH2O**^**]**^**–**^ as viewed along the equatorial coordination plane. (c) Solid-state structures of **[CoL**^**OCH2O**^**]**^**–**^ (left), **[NiL**^**OCH2O**^**]**^**–**^ (center), and **[ZnL**^**OCH2O**^**]**^**–**^ (right) as viewed through the macrocycle-defined cavity. For all solid-state structures, the [K(18-crown-6)]^+^ cation and cocrystallized solvent molecules are omitted for clarity. Thermal ellipsoids are shown at the 50% level.

Despite the conformationally constrained nature of **H**_**3**_**L**^**OCH2O**^, metallation to form monometallic complexes can be carried out following deprotonation of the amide N–H bonds. Treatment with KH in *N*,*N*-dimethylacetamide (DMA) followed by the addition of M(OAc)_2_ (M = Co, Ni, Zn) yields the four-coordinate complexes **[ML**^**OCH2O**^**]**^**–**^ (Figure 3). Crystallographic characterization of these metallates as their [K(18-crown-6)]^+^ salts revealed the conservation of a tight void space bound by the faces of the tethered aromatic rings. This void encapsulates the open axial coordination site, with the metal center buried beneath the rim of the cavity by approximately 6 Å. All three complexes crystallize as infinite one-dimensional chains, with [K(18-crown-6)]^+^ cations each bridging two metallate species through contacts with amidyl or acetal O atoms along one crystallographic axis (Figures S38–S40). Both **[CoL**^**OCH2O**^**]**^**–**^ and **[ZnL**^**OCH2O**^**]**^**–**^ adopt nearly idealized trigonal monopyramidal geometries; all equatorial N–M–N bond angles fall between 117 and 121° while the complexes’ τ_4_ parameters^50^ lie squarely in the range expected for trigonal monopyramidal geometries (Co = 0.86; Zn = 0.84). The nickel(II) congener **[K(18-crown-6)][NiL**^**OCH2O**^**]** contains two formula units in the asymmetric unit (Figure S42). The primary coordination spheres of both crystallographically independent nickel complexes show a distortion from trigonal symmetry, with each complex bearing one large equatorial N–Ni–N angle (130.09(12) and 141.78(7)°)^51^ and τ_4_ values describing geometries intermediate between trigonal monopyramidal and sawhorse (τ_4_ = 0.80 and 0.72, respectively). While a high-spin *d*^8^ complex in a (pseudo) trigonal monopyramidal geometry is susceptible to Jahn–Teller distortion,^52^ the magnitude of distortion seen for the **[NiL**^**OCH2O**^**]**^**–**^ unit with τ_4_ = 0.72 is surprising. Of the previously reported four-coordinate nickel(II) complexes bearing TREN-derived ligands,^52−56^ the largest equatorial N–Ni–N angle occurs within Cummins’ and Nocera’s Ni_2_ metallocryptand (133.83(17)°).^54^ Accordingly, although the large N–Ni–N angle of 141.78(7)° observed here is likely not representative of the average geometry present in solution, it does provide a snapshot of the equatorial distortions that are conformationally accessible in complexes of the [L^OCH2O^]^3–^ ligand.

**Figure 4:**
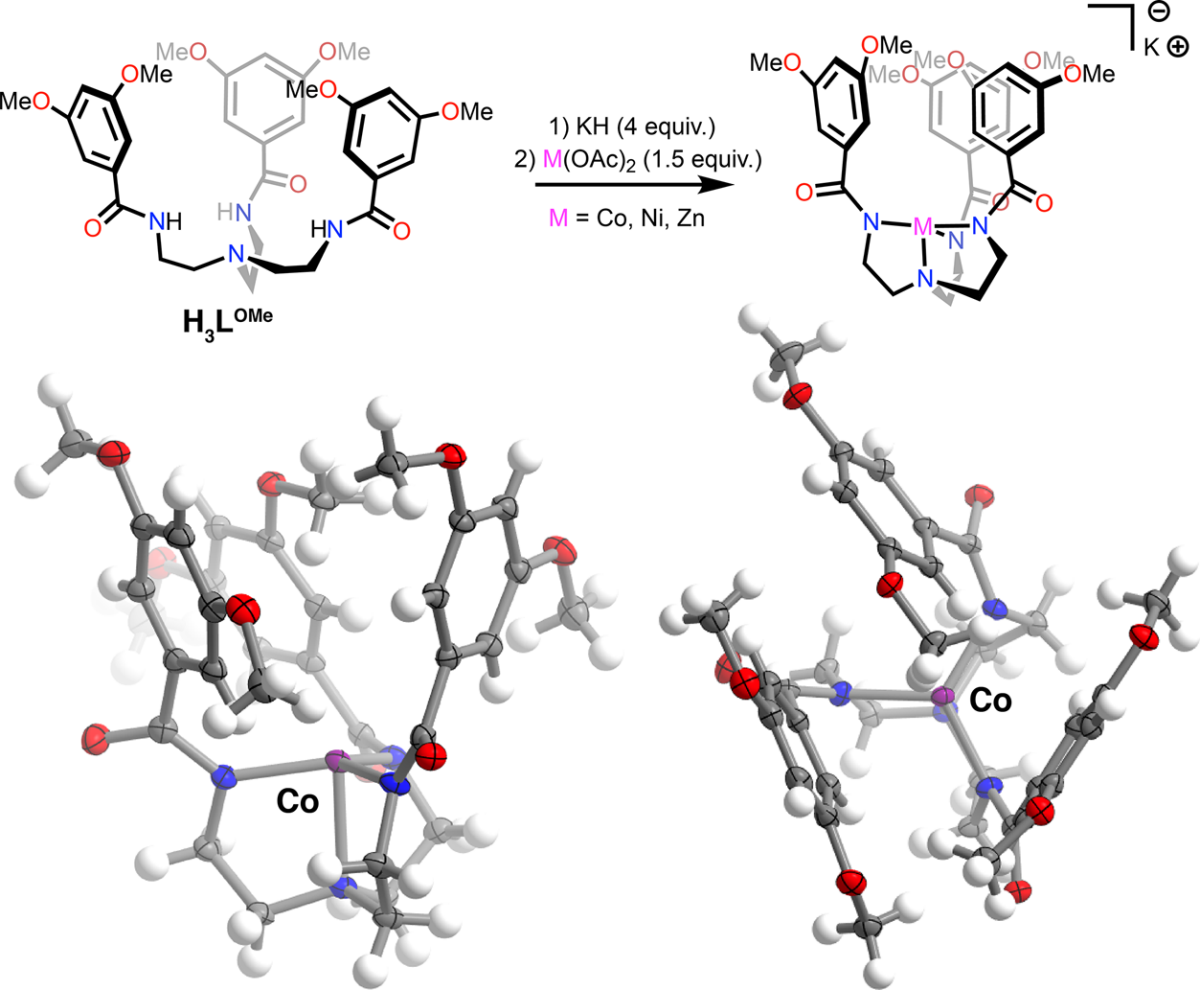
Synthesis of **K[ML**^**OMe**^**]** (top) and solid-state structure of **[CoL**^**OMe**^**]**^**–**^ (bottom). The [K(18-crown-6)]^+^ cation and cocrystallized solvent molecules are omitted for clarity. The nickel and zinc congeners pack in an isomorphous fashion. Thermal ellipsoids are shown at the 50% level.

To facilitate the analysis of the structural effects of macrocyclization on the resultant complexes, metallation of the 3,5-dimethoxyphenyl-substituted tris-amide^57^**H**_**3**_**L**^**OMe**^ was carried out in a similar fashion as for **H**_**3**_**L**^**OCH2O**^. The corresponding four-coordinate cobalt(II), nickel(II), and zinc(II) metallates were crystallographically characterized as their [K(18-crown-6)]^+^ salts and adopt isomorphous structures (Figures 4 and S35 and S36). Infinite one-dimensional chains arise through the contacts of each [K(18-crown-6)]^+^ cation with amidyl O atoms on two adjacent **[ML**^**OMe**^**]**^**–**^ units (Figure S41). As for the complexes of [L^OCH2O^]^3–^, **[CoL**^**OMe**^**]**^**–**^ (τ_4_ = 0.85) and **[ZnL**^**OMe**^**]**^**–**^ (τ_4_ = 0.84) exhibit nearly idealized trigonal monopyramidal geometries, while **[NiL**^**OMe**^**]**^**–**^ distorts toward a sawhorse conformation (τ_4_ = 0.78, largest N–Ni–N angle = 127.81(5)°). In each structure, the edge of one arene ring points inward toward the metal site and sits between the other two rings, which orient their faces inward (Figure 4, bottom right). However, the ^1^H NMR spectra of all three **[ML**^**OMe**^**]**^**–**^ congeners are consistent with a *C*_3*v*_ symmetric structure in solution, indicating conformational fluxionality on the NMR time scale (Figures S11–S14). Accordingly, while both ligands capably promote a trigonal coordination environment about the metal and the presence of an open axial coordination site, macrocyclized [L^OCH2O^]^3–^ alone enforces a rigid, unoccupied channel that leads to the primary coordination sphere.

**Figure 5:**
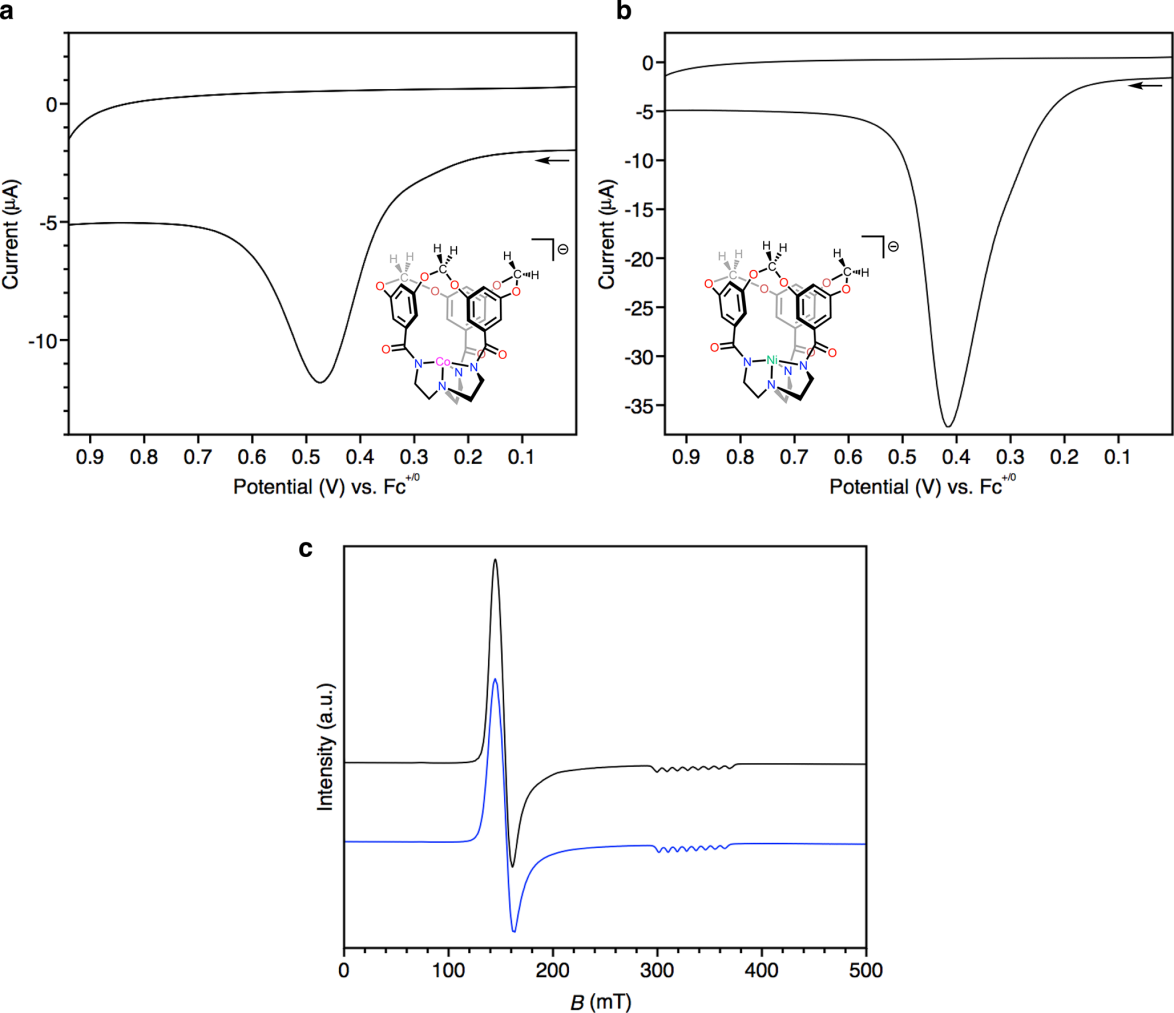
Portions of the cyclic voltammograms (scan rate = 0.1 V/s) for (a) **[CoL**^**OCH2O**^**]**^**–**^ and (b) **[NiL**^**OCH2O**^**]**^**–**^ in CH_2_Cl_2_ ([N(*n*-Bu)_4_]PF_6_ electrolyte). (c) X-band EPR spectra of **[CoL**^**OCH2O**^**]**^**–**^ (black line) and **[CoL**^**OMe**^**]**^**–**^ (blue line) recorded at 10 K in frozen CH_2_Cl_2_.

As is common for TREN-derived LX_3_-type ligands,^58,59^ both [L^OCH2O^]^3–^ and [L^OMe^]^3**–**^ promote high-spin electronic configurations, with Evans method measurements yielding values consistent with *S* = 3/2 and *S* = 1 configurations for the Co and Ni complexes, respectively (Table 1). Cyclic voltammograms of **[CoL**^**OCH2O**^**]**^**–**^ and **[NiL**^**OCH2O**^**]**^**–**^ reveal electrochemically irreversible waves that we attribute to formal Co^3+/2+^ and Ni^3+/2+^ redox events (Figures 5a,b and S27 and S28). These events remain electrochemically irreversible at scan rates of as fast as 10 V/s and do not appear in the voltammograms of **[ZnL**^**OCH2O**^**]**^**–**^, substantiating their assignments as metal-based events (Figure S29). The onset potentials of these waves compare well with those seen for the corresponding cobalt(II) and nickel(II) dimetallocryptands reported by Cummins and Nocera.^54,60^ However, they are shifted anodically when compared to four-coordinate tris-amidylamine cobalt(II) and nickel(II) complexes reported by others.^10,12^ These differences may reflect the rigidity of [L^OCH2O^]^3–^ hindering the distortion of **[CoL**^**OCH2O**^**]**^**–**^ and **[NiL**^**OCH2O**^**]**^**–**^ toward tetragonal coordination geometries upon oxidation.^61^ The EPR spectrum of **[CoL**^**OCH2O**^**]**^**–**^ in frozen CH_2_Cl_2_ solution (10 K) displays axial symmetry with *g* values of 4.49 and 2.01 (Figures 5c and S24). An eight-line pattern due to hyperfine coupling with ^59^Co (*I* = 7/2, 100% abundance) is evident for the latter feature. The axial nature of this spectrum indicates that **[CoL**^**OCH2O**^**]**^**–**^ in solution maintains the trigonally symmetric coordination geometry seen in the solid state by X-ray diffraction. These EPR spectral parameters are consistent with those seen for other trigonal monopyramidal cobalt complexes with *S* = 3/2 ground states^11−13,51,55^ and additionally are nearly identical to those for **[CoL**^**OMe**^**]**^**–**^ (Table 1 and Figures 5c and S25).

**Table 1:** Solution-State Magnetic Moments and Selected EPR Fitting Parameters

Compound	Effective Magnetic Moment (μ_B_)^a^	*g*_1_	*g*_*2*_	*A* (^59^Co) (MHz)
**[CoL**^**OCH2O**^**]**^**–**^	4.2	4.49	2.01	271.4
**[CoL**^**OMe**^**]**^**–**^	4.2	4.48	2.01	257.0
**[NiL**^**OCH2O**^**]**^**–**^	3.7	–	–	–
**[NiL**^**OMe**^**]**^**–**^	3.3	–	–	–

a Measured in solution using the Evans method at 23 °C.

**Figure 6:**
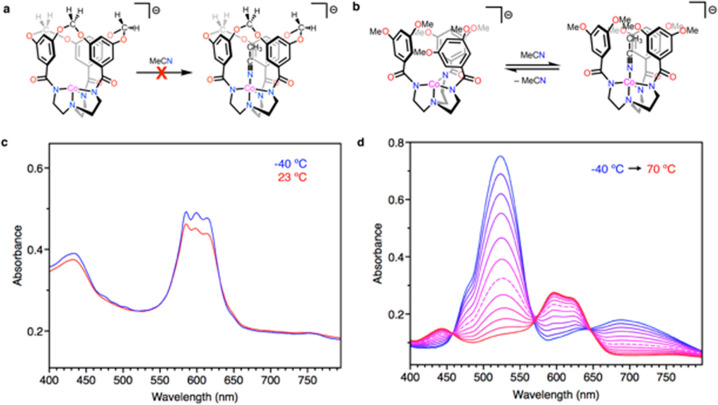
(a) Exclusion of MeCN from the coordination sphere of **[CoL**^**OCH2O**^**]**^**–**^, while (b) binding to **[CoL**^**OMe**^**]**^**–**^ occurs readily at ambient and low temperatures. (c) UV–visible spectra of **[CoL**^**OCH2O**^**]**^**–**^ in MeCN at 23 and −40 °C. (d) UV–visible spectra of **[CoL**^**OMe**^**]**^**–**^ in acetonitrile at temperatures of between −40 and 70 °C (spectra were obtained at intervals of 10 °C). The dashed line corresponds to the 20 °C measurement.

**Figure 7:**
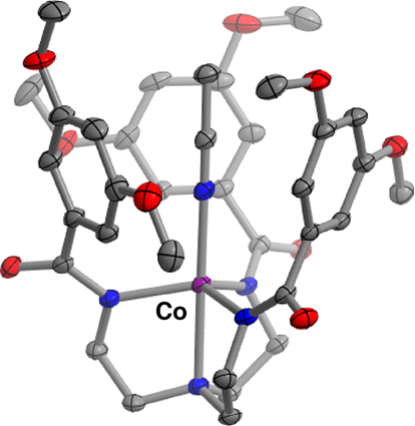
Solid-state structure of **[Co(NCMe)L**^**OMe**^**]**^**–**^. Hydrogen atoms, the potassium countercation, and cocrystallized acetonitrile molecules have been omitted for clarity. Thermal ellipsoids are shown at the 50% level.

We hypothesized that the rigid apertures guarding the coordination space in **[ML**^**OCH2O**^**]**^**–**^ could gate the access of exogenous substrates into the cavity interior. Barring ingress through the equatorial plane, substrate binding to the open coordination site relies on penetration through the aperture defined by the three aromatic rings and their acetal linkages. This scenario evokes the diffusion of substrates through narrow channels in zeolite catalysts and represents a design feature that could lead to size-based selectivities in homogeneous metal-based reactivity. Notably, the dissolution of turquoise **[CoL**^**OCH2O**^**]**^**–**^ in the coordinating solvent acetonitrile (MeCN) does not result in any noticeable color changes. UV–visible spectra at both 23 and −40 °C show exclusively *d*–*d* bands that correspond to a trigonal monopyramidal high-spin Co center, indicating that acetonitrile does not coordinate to the open axial site (Figure 6a,c). In contrast, the dissolution of green **[CoL**^**OMe**^**]**^**–**^ in acetonitrile gives a deep-purple solution, and the electronic spectra of these solutions show peaks corresponding to both trigonal monopyramidal and trigonal bipyramidal Co centers.^12,62,63^ Variable-temperature measurements evidence that the binding of acetonitrile to **[CoL**^**OMe**^**]**^**–**^ becomes increasingly favored at lower temperatures (Figure 6b,d). Cooling concentrated acetonitrile solutions of **[CoL**^**OMe**^**]**^**–**^ to −35 °C precipitated both green crystals of **[CoL**^**OMe**^**]**^**–**^ and purple crystals of **[Co(NCMe)L**^**OMe**^**]**^**–**^. Structural determination of the latter by X-ray diffraction (Figure 7) reveals an axially bound acetonitrile ligand (Co–N_NCMe_ = 2.113(2) Å) and an elongation of the *trans* Co–N_amine_ bond (2.299(2) Å vs 2.1605(19) Å for the trigonal monopyramidal complex). Space for the bound acetonitrile to reside within the coordination sphere is created by a rearrangement of the arene rings compared to the structure of **[CoL**^**OMe**^**]**^**–**^ (*vide supra*), which rotates such that all three orient their faces inward and surround the nitrile. The attendant π–π interactions between MeCN and the arene rings may enable observable coordination at ambient temperatures, as related tris-amidylamine cobaltates bearing alkyl substituents have been reported to retain four-coordinate geometries in acetonitrile solution.^13^ Examples of trigonal monopyramidal cobalt(II) complexes that do coordinate acetonitrile include those bearing weaker *trans* donors^63,64^ and cationic complexes,^65^ both of which can be reasoned to exhibit enhanced Lewis acidity. Note that variable-temperature UV–visible spectroscopy measurements for acetonitrile solutions of **[NiL**^**OMe**^**]**^**–**^ show changes potentially indicative of solvent coordination at lower temperatures (Figure S20), whereas ambient-temperature and low-temperature spectra of [**NiL**^**OCH2O**^**]**^**–**^ in acetonitrile are essentially unchanged (Figure S21), again suggesting that only the nonmacrocyclized ligand permits acetonitrile coordination. Variable-temperature ^1^H NMR spectra of **[ZnL**^**OMe**^**]**^**–**^ in CD_3_CN show minimal changes with the temperature (Figure S17), indicating that the 18-valence-electron zinc center resists acetonitrile coordination.

**Figure 8:**
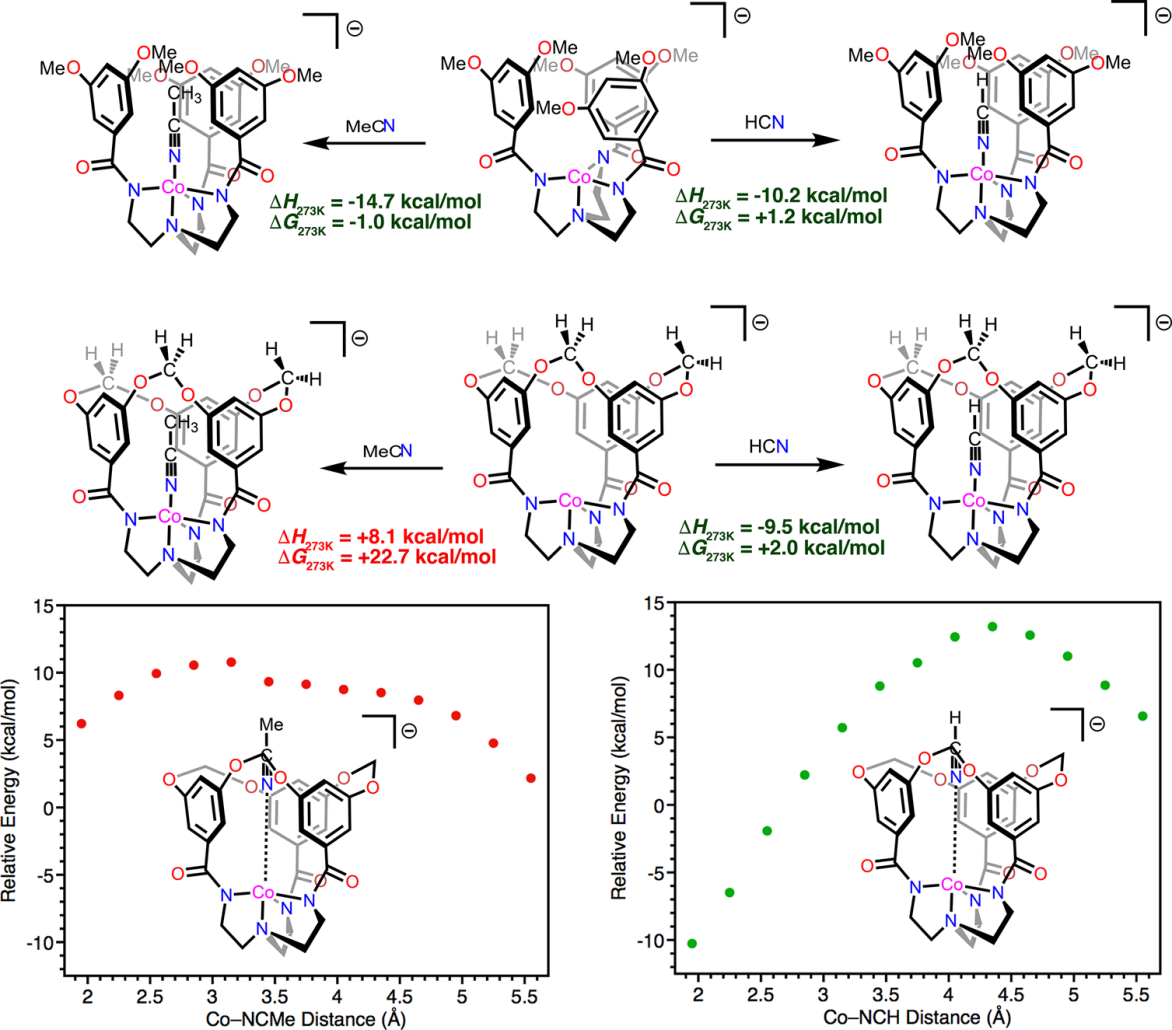
Density functional theory (DFT) investigations into the coordination of nitrile ligands to the axial sites in **[CoL**^**OCH2O**^**]**^**–**^ and **[CoL**^**OMe**^**]**^**–**^. Top: Calculated enthalpy and Gibbs free energy values for (left) acetonitrile and (right) hydrogen cyanide coordination to the cobalt complexes at standard temperature. Bottom: Relaxed surface scans along the Co–N_nitrile_ coordinate for (left) acetonitrile and (right) hydrogen cyanide cavity penetration and coordination. Energy values are relative to the summed single-point energies of **[CoL**^**OCH2O**^**]**^**–**^ and free nitrile.

In accordance with experimental observations, dispersion-corrected density functional theory (DFT) calculations indicate a substantial difference in the thermodynamics of acetonitrile binding between the two cobalt complexes (Figure 8). At the OLYP^66^ level (def2-TZVP(-f) basis set on Co; def2-SVP basis set on light atoms),^67^ acetonitrile binding to *S* = 3/2 **[CoL**^**OMe**^**]**^**–**^ to give **[Co(NCMe)L**^**OMe**^**]**^**–**^ at standard temperature (273.15 K) is computed to be exothermic (Δ*H* = −14.7 kcal/mol) with a Gibbs free energy of reaction that is nearly thermoneutral (Δ*G* = −1.0 kcal/mol). In contrast, the binding of acetonitrile to **[CoL**^**OCH2O**^**]**^**–**^ is computed to be endothermic (Δ*H* = +8.1 kcal/mol) and accordingly strongly endergonic (Δ*G* = +22.7 kcal/mol). The metrical parameters about cobalt in the optimized structure of **[Co(NCMe)L**^**OCH2O**^**]**^**–**^ do not differ substantially from those in **[Co(NCMe)L**^**OMe**^**]**^**–**^ with the exception of the bond length between Co and the axial tertiary amine (Tables S10 and S13); this is a reflection of both the increased conformational flexibility possessed by the [L^OMe^]^3–^ ligand and the very shallow potential energy surface along the Co–N_amine_ coordinate for **[Co(NCMe)L**^**OMe**^**]**^**–**^ (see the Supporting Information section S6.2).^68^ Rather, it seems apparent that the rigid steric profile of the narrow void in **[CoL**^**OCH2O**^**]**^**–**^ precludes MeCN binding. Support for this hypothesis was obtained by computationally exploring intracavity binding of the smaller and rigorously linear nitrile HCN. The primary coordination spheres in the optimized geometries of **[Co(NCH)L**^**OCH2O**^**]**^**–**^ and **[Co(NCH)L**^**OMe**^**]**^**–**^ are readily comparable with significant discrepancies seen only on comparing the Co–N_amine_ distances (Tables S11 and S14). Importantly, however, the calculated enthalpy and free energy of HCN binding exhibit only very minor differences between these cobalt complexes and are similar in magnitude to those computed for the binding of MeCN to **[CoL**^**OMe**^**]**^**–**^ (Figure 8). This observation suggests that the intracavity binding of very small ligands in **[ML**^**OCH2O**^**]**^**–**^ complexes is sterically feasible.

The presence of a gate protecting the cavity interior may result in cavity penetration becoming the rate-limiting event in a bimolecular metal-based reaction.^29^ While ligand-binding events at unsaturated metal centers often have small activation barriers associated with coordination sphere rearrangements,^69^ the rate-limiting event for intracavity coordination could plausibly be penetration of the void by the incoming ligand. To explore this notion, relaxed surface scans were performed to investigate the energetics of MeCN and HCN ingress and egress through the narrow void (Figure 8, bottom). In both cases, a significant increase in Δ*E* (i.e., Δ*H* at 0 K) occurs as the nitrile progresses through the void, with maxima for both ligands occurring at Co–N_nitrile_ distances outside of the van der Waals contact. Indeed, it seems intuitive that these distances would maximize steric pressures given both the slightly convex profile of the arene-defined void and the potential for clashing of the nitrile with the methylene groups lining the cavity crest. The methyl group of an intracavity-ligated MeCN must reside near the narrowest region of the void, and the attendant steric clashing offsets the stabilization afforded by a weak Co–N_nitrile_ coordinative bond. However, HCN is apparently small enough that steric pressures subside at shorter Co–N_nitrile_ distances to yield a net energetically favorable binding event. Note that attempts were made to synthesize **[Co(NCH)L**^**OCH2O**^**]**^**–**^ by exposing **[CoL**^**OCH2O**^**]**^**–**^ to HCN (***Caution!**Hydrogen cyanide is a highly toxic gas*) but were hampered by competitive demetallation to yield **H**_**3**_**L**^**OCH2O**^ along with unidentified cobalt-containing species.

The inaccessibility of the void within **[CoL**^**OCH2O**^**]**^**–**^ to MeCN likely reflects a high degree of rigidity and a low proclivity for the cavity to undergo guest-responsive conformational changes. In this way, the [L^OCH2O^]^3–^ ligand differs from the calix[6]arene-bearing “funnel complexes” championed by Reinaud, which generally display guest-adaptive cavities.^29,70^ Indeed, it is well known that calix[6]arenes display conformational flexibility enabled by rotations about the C_sp^3^_–C_arene_ bonds.^71,72^ In contrast, the macrocyclic motif within the [L^OCH2O^]^3–^ ligand more closely approximates the structure of Cram’s cavitands,^44^ wherein each arene is linked to its neighboring rings through two points of attachment, imbuing the macrocycle with greatly enhanced rigidity and less conformational adaptability.

## Conclusions

We have shown that macrocyclization of a TREN-derived proligand can engender a rigid and narrow void that is oriented on top of the metal binding pocket. Despite its rigidity, **H**_**3**_**L**^**OCH2O**^ can undergo deprotonative metallation to yield four-coordinate complexes that localize an open coordination site within the ligand cavity. The cavity’s narrow profile tightly regulates the arrival of substrates at the open coordination site and precludes the binding of acetonitrile on the basis of size, as clashing of the nitrile with atoms near the cavity rim thermodynamically outweighs the formation of a weak M–N_nitrile_ bond. Additionally, calculations demonstrate that the entry of even small-profile species into the void space must overcome a significant kinetic barrier that is likely to slow substrate ingress. We envision that the localization of reactive metal-bound motifs within the cavity of complexes bearing the [L^OCH2O^]^3–^ ligand will allow for kinetic stabilization and characterization of metal-bound species that have traditionally proven elusive.

## Experimental Section

### General Considerations

All manipulations were carried out under an atmosphere of purified dinitrogen using standard Schlenk and glovebox techniques. Unless otherwise stated, reagent-grade starting materials were purchased from commercial sources and either used as received or purified by standard procedures.^73^ Unless otherwise stated, organic solvents were deoxygenated and dried using a Pure Process Technologies solvent purification system. *N*,*N*-Dimethylacetamide (DMA) and dimethyl sulfoxide-*d*_6_ were stored over activated 3 Å molecular sieves, transferred via cannula into a separate flask, sparged with N_2_ for 1 h, and stored over fresh 3 Å molecular sieves in the glovebox prior to use. Chloroform-*d* was stirred with CaH_2_, distilled into a separate flask, degassed via freeze–pump–thaw cycles, and stored over activated 3 Å molecular sieves in the glovebox prior to use. Methanol was neither deoxygenated nor dried and was used as received. Molecular sieves (3 Å) and Celite were separately preactivated in a 180 °C oven overnight, transferred into a round-bottomed flask and heated under vacuum (*P* < 100 mTorr) at a temperature in excess of 200 °C for at least 12 h, and then stored in the glovebox. Tetra(*n*-butylammonium) hexafluorophosphate for electrochemical measurements was recrystallized three times from ethanol and then dried under vacuum with P_2_O_5_ at 120 °C until the pressure reached 50 mTorr.

Solution ^1^H and ^13^C{^1^H} nuclear magnetic resonance (NMR) spectra were recorded on a Bruker DPX-400 or DPX-500 spectrometer locked on the signal of deuterated solvents. ^1^H and ^13^C{^1^H} chemical shifts are reported in parts per million relative to SiMe_4_ (^1^H and ^13^C δ = 0.0 ppm) with reference to residual solvent resonances. Electronic absorption measurements were recorded using either a PerkinElmer Lambda 35 UV–vis spectrometer (ambient-temperature measurements) or an Agilent Cary 6000i UV–vis–NIR spectrometer (variable-temperature measurements). Samples were prepared in the glovebox and sealed in a quartz cuvette (1 cm or 1 mm path length). X-band electron paramagnetic resonance (EPR) measurements were carried out on a Bruker EMXplus spectrometer (microwave frequency of 9.382 GHz). Samples were prepared as solutions in CH_2_Cl_2_ in a glovebox, glassed via flash-cooling in liquid nitrogen, and loaded into the spectrometer. Electrochemical measurements were performed using a CH Instruments 620 D potentiostat with a three-electrode setup, including a Ag/AgNO_3_ (1 M) reference electrode (CHI111), a Pt wire counter electrode (CHI115, surface area in solution of 0.14 cm^2^), and a glassy carbon working electrode (CHI104, 3 mm diameter). Solutions were prepared using dry and degassed CH_2_Cl_2_ at a concentration of 1 mM metal complex. The [NBu_4_]PF_6_ electrolyte concentration was 0.1 M. Voltammograms were referenced to the Cp_2_Fe^+/0^ couple by using ferrocene as an internal standard. Solution-phase effective magnetic moments were determined using the Evans method. A dried solid analyte sample was dissolved in 0.800 mL of 9:1 v/v CH_2_Cl_2_/PhCF_3_. This solution was placed in a borosilicate NMR tube along with a flame-sealed glass capillary containing a 4:1 v/v CH_2_Cl_2_/PhCF_3_ internal standard. Elemental analyses were performed on a PerkinElmer 2400 Series II Analyzer at the CENTC Elemental Analysis Facility, University of Rochester.

### Preparation of Anhydrous Cobalt(II) Acetate

A flame-dried three-necked round-bottomed flask outfitted with a reflux condenser was evacuated and then backfilled with nitrogen a total of three times. The flask was charged with acetic anhydride (76.0 mL, 805 mmol) and sparged with N_2_ for 1 h. Cobalt(II) acetate tetrahydrate (20.0 g, 79.6 mmol) was then added to the flask under a N_2_ purge, and the solution was heated to reflux for 12 h. After cooling, the solution was filtered using a Schlenk frit, and while an inert atmosphere was maintained, the light-pink solid was transferred into the glovebox and washed with 5 × 50 mL portions of Et_2_O. The solid was then dried under vacuum and was confirmed to be anhydrous via FT-IR spectroscopy (KBr pellet). Yield = 13.7 g, 77.4 mmol, 96%.

### Preparation of Anhydrous Nickel(II) Acetate

A flame-dried three-necked round-bottomed flask outfitted with a reflux condenser was evacuated and then backfilled with nitrogen a total of three times. The flask was charged with acetic anhydride (76.0 mL, 805 mmol) and sparged with N_2_ for 1 h. Nickel(II) acetate tetrahydrate (20.0 g, 79.7 mmol) was then added to the flask under a N_2_ purge, and the solution was heated to reflux for 12 h. After cooling, the solution was filtered using a Schlenk frit, and while an inert atmosphere was maintained, the light-green solid was transferred into the glovebox and washed with 5 × 50 mL portions of Et_2_O. The solid was then dried under vacuum and was confirmed to be anhydrous via FT-IR spectroscopy (KBr pellet). Yield = 13.8 g, 78.1 mmol, 97%.

### Synthesis of **H_3_L^OBn^**

*Note: All water used in this preparation was 18.2 MΩ nanopure water. All glassware used in this preparation was soaked in a KOH/iPrOH bath, rinsed with water three times, and dried prior to use. Neglecting these precautions led to reaction mixtures that were darkened in color and resulted in difficulties purifying the subsequently synthesized****H*_*3*_*L*^*OCH2O*^***. We attribute these observations to the proclivity of TREN-derived tris-amides to act as very strong chelators of halides and small inorganic anions.*(74) A 1 L three-necked round-bottom flask was charged with 3,5-di(benzyloxy)benzoic acid (39.0 g, 117 mmol, 3.3 equiv) and 300 mL of CH_2_Cl_2_. Solid 1,1′-carbonyldiimidazole (18.9 g, 117 mmol, 3.3 equiv) was added slowly, and the reaction was then stirred for 1 h. After purging the headspace with N_2_ to ensure the liberation of evolved CO_2_, tris(2-aminoethyl)amine (5.30 mL, 35.3 mmol) was then added in a single portion via syringe, and the reaction was allowed to stir for 12 h. The reaction mixture was then washed with water five times in a separatory funnel. The organic layer was dried under vacuum (*P* = 30 mTorr) to afford a colorless powder. Yield = 31.6 g, 29.0 mmol, 82%. ^1^H NMR (400.1 MHz, DMSO-*d*_6_): δ = 8.41 (br s, 3H, −N*H*), 7.39 (br s, 30H, −Ph), 7.10 (br s, 6H, *o*-Ar), 6.78 (br s, 3H, *p*-Ar), 5.07 (br s, 12H, −C*H*_2_Ph), 3.35 (br s, 6H, −NHC*H*_2_CH_2_N), 2.71 (br s, 6H, −NHCH_2_C*H*_2_N) ppm. ^13^C{^1^H} NMR (125.8 MHz, DMSO-*d*_6_) δ = 165.74, 159.33, 136.77, 136.64, 128.44, 127.89, 127.71, 106.19, 104.48, 69.46, 37.93 ppm. Calculated elemental analysis for C_69_H_66_H_4_O_9_: C, 75.66%; H, 6.07%; N, 5.11%. Found: C, 74.82%; H, 6.17%; N, 5.26%.

### Synthesis of **H_3_L^OH^**

*Note: All water used in this preparation was 18.2 MΩ nanopure water. All glassware used in this preparation was soaked in a KOH/iPrOH bath, rinsed with water three times, and dried prior to use. Neglecting these precautions led to reaction mixtures that were darkened in color and resulted in difficulties in purifying the subsequently synthesized****H*_*3*_*L*^*OCH2O*^***. We attribute these observations to the proclivity of TREN-derived tris-amides to act as very strong chelators of halides and small inorganic anions.*(74) In a three-necked round-bottomed flask equipped with a reflux condenser and under an N_2_ atmosphere, a solution of **H**_**3**_**L**^**OBn**^ (22.6 g, 20.6 mmol) in methanol (550 mL) was added under an N_2_ purge. Subsequently added under an N_2_ purge were Pd/C (10 wt % Pd; 2.20 g, 2.07 mmol Pd, 0.1 equiv) and then 1,4-cyclohexadiene (19.6 mL, 207 mmol, 10 equiv). The reaction was then heated to 60 °C for 16 h. The resulting black suspension was filtered, and the filtrate was then dried *in vacuo* to yield a hygroscopic colorless solid. Yield: 9.79 g, 17.6 mmol, 85%. ^1^H NMR (400.1 MHz, DMSO-*d*_6_) δ = 9.40 (s, 6H, −O*H*), 8.19, (t, *J* = 3H, −N*H*), 6.65 (d, *J* = 2 Hz, 6H, *o*-Ar), 6.33 (t, *J* = 2 Hz, 3H, *p*-Ar), 3.28 (q, *J* = 7 Hz, 6H, −NHC*H*_2_CH_2_N), 2.65 (t, *J* = 7 Hz, 6H, −NHCH_2_C*H*_2_N) ppm. ^13^C{^1^H} NMR (125.8 MHz, DMSO-*d*_6_) δ = 166.54, 158.31, 136.94, 105.45, 105.04, 53.47, 37.79 ppm. Calculated analysis for C_27_H_31_N_4_O_9.5_ (**H_3_L^OH^**·0.5 H_2_O): C, 57.54%; H, 5.54%; N, 9.78%. Found: C, 57.45%; H, 5.74%; N, 9.95%.

### Synthesis of **H_3_L^OCH2O^**

A three-necked 2 L round-bottomed flask (which had been previously soaked in a KOH/*i*PrOH bath, washed with nanopure water, and dried) was equipped with a 500 mL addition funnel, purged with N_2_ for 30 min, and then charged with **H**_**3**_**L**^**OH**^ (9.79 g, 17.7 mmol) and K_2_CO_3_ (43.9 g, 318 mmol, 18 equiv). To the flask was added 1 L of dry and deoxygenated DMF via cannula transfer. The addition funnel was charged with a solution of CH_2_BrI (5.32 mL, 70.6 mmol, 4 equiv) in dry and deoxygenated DMF (500 mL) using a cannula. The CH_2_BrI solution was then added dropwise to the reaction mixture with stirring over the course of 13 h at 23 °C and then stirred for an additional 9 h. Subsequently, the temperature was increased to 60 °C and the reaction mixture was stirred for another 24 h. Upon cooling, the reaction mixture was concentrated under reduced pressure. The resulting pink/red residue was placed in a glass coarse fritted filter thimble and extracted with chloroform using a Soxhlet extractor over the course of 2 weeks. The resulting suspension was then dried *in vacuo*, and the off-white solid was serially triturated at 50 °C with water, methanol, and hexanes, yielding **H**_**3**_**L**^**OCH2O**^ as a colorless solid after drying *in vacuo*. Yield: 0.880 g, 1.49 mmol, 8.4%. ^1^H NMR (400.1 MHz, DMSO-*d*_6_): δ = 7.25 (d, *J* = 2 Hz, 6H, *o*-Ar), 7.23 (br s, 3H, −N*H*), 6.61 (t, *J* = 2 Hz, 3H, *p*-Ar), 6.18 (d, *J* = 7.7 Hz, 3H, −OC*H*_*2*_O−), 5.45 (d, *J* = 7.7 Hz, 3H, −OC*H*_*2*_O−), 3.47 (m, 6H, −NHC*H*_2_CH_2_N), 2.60 (t, *J* = 6 Hz, 6H, −NHCH_2_C*H*_2_N) ppm. ^13^C{^1^H} NMR (125.8 MHz, DMSO-*d*_6_): δ = 165.39, 157.39, 135.85, 112.55, 112.01, 92.71, 50.23, 35.51 ppm. Calculated elemental analysis for C_30_H_30_N_4_O_9_: C, 61.01%; H, 5.12%; N, 9.49%. Found: C, 59.55%; H, 5.08%; N, 9.32%.

### Synthesis of **H_3_L^OMe^**

A 1 L Schlenk flask was charged with a 500 mL of a CH_2_Cl_2_ solution of 3,5-dimethoxybenzoic acid (82.3 g, 452 mmol, 3.3 equiv) and then purged with N_2_. While under the N_2_ purge, 1,1′-carbonyldiimidazole (77.7 g, 475 mmol, 3.5 equiv) was added as a solid. The reaction mixture was then allowed to stir for 1 h under N_2_. After the headspace was purged with N_2_ to ensure the liberation of evolved CO_2_, tris(2-aminoethyl)amine (20.5 mL, 137 mmol) was then added in a single portion via syringe, and the reaction was allowed to stir for 12 h. After the reaction mixture was washed five times with H_2_O using a separatory funnel, the organic layer was dried *in vacuo*. The resulting pale-yellow residue was recrystallized from THF to yield **H**_**3**_**L**^**OMe**^ as colorless crystals. Yield: 81.4 g, 93%. The ^1^H NMR spectrum of this compound matched that reported previously.^57^

### Synthesis of **[K(18-crown-6)][CoL^OCH2O^]**

In the glovebox, a scintillation vial was charged with **H**_**3**_**L**^**OCH2O**^ (0.100 g, 0.169 mmol) and KH (0.027 g, 0.68 mmol, 4 equiv). DMA (2 mL) was added, and the effervescent solution was stirred for 1 h, during which time bubbling ceased. This solution was filtered through Celite to remove residual KH, added to solid Co(OAc)_2_ (0.046 g, 0.25 mmol, 1.5 equiv), and stirred for 12 h. After filtering through Celite and washing the filter cake with additional DMA (4 mL), Et_2_O was added to the filtrate to precipitate the desired complex. This suspension was stirred for 30 min and then filtered through a fine-porosity fritted funnel. The crude complex remaining on the funnel was washed five times with a 5:1 Et_2_O/DMA solution. The resulting greenish residue was then dissolved in a solution of 18-crown-6 (0.054 g, 0.20 mmol, 1.2 equiv) in CH_2_Cl_2_ (2 mL) and stirred for several minutes before being filtered through Celite. The filtrate was dried *in vacuo* and then taken up in a minimum amount of MeCN. Storage in a glovebox freezer at −35 °C for 3 days yielded turquoise crystals, which were harvested and washed with Et_2_O to remove a small amount of cocrystallized 18-crown-6. The crystals were redissolved in CH_2_Cl_2_ and then dried *in vacuo* at 60 °C to yield **[K(18-crown-6)][CoL**^**OCH2O**^**]** that is free from residual MeCN. Yield: 0.063 g, 0.066 mmol, 39%. ^1^H NMR (400.1 MHz, DMSO-*d*_6_): δ = 75.4, 3.6 (18-c-6), −0.2, −10.3 ppm. μ_eff_ = 4.2 μ_B_ (Evans method, ^19^F NMR, CH_2_Cl_2_/PhCF_3_, 23 °C). Material for elemental analysis was prepared by the recrystallization of crude and crown ether-free material from a DMA solution upon vapor diffusion of Et_2_O. Calculated analysis for C_30_H_27_N_4_O_9_CoK: C, 52.56%; H, 3.97%; N, 8.17%. Found: C, 52.77%; H, 4.12%; N, 8.56%.

### Synthesis of **[K(18-crown-6)][NiL^OCH2O^]**

In the glovebox, a scintillation vial was charged with **H**_**3**_**L**^**OCH2O**^ (0.100 g, 0.169 mmol) and KH (0.027 g, 0.68 mmol, 4 equiv). DMA (2 mL) was added, and the effervescent solution was stirred for 1 h, during which time bubbling ceased. This solution was filtered through Celite to remove residual KH and then added to solid Ni(OAc)_2_ (0.045 g, 0.25 mmol, 1.5 equiv) and stirred for 12 h. After filtering through Celite and washing the filter cake with additional DMA (4 mL), Et_2_O was added to the filtrate to precipitate the desired complex. This suspension was stirred for 30 min and then filtered through a fine-porosity fritted funnel. The crude complex remaining on the funnel was washed five times with a 5:1 Et_2_O/DMA solution. The resulting light-orange residue was then dissolved in a solution of 18-crown-6 (0.054 g, 0.20 mmol, 1.2 equiv) in CH_2_Cl_2_ (2 mL) and stirred for several minutes before being filtered through Celite. The filtrate was dried *in vacuo* and then taken up in a minimum amount of MeCN. Storage in a glovebox freezer at −35 °C for 3 days yielded orange/pink crystals, which were harvested and washed with Et_2_O to remove a small amount of cocrystallized 18-crown-6. The crystals were redissolved in CH_2_Cl_2_ and then dried *in vacuo* at 60 °C to yield **[K(18-crown-6)][NiL**^**OCH2O**^**]** that is free from residual MeCN. Yield: 0.082 g, 0.086 mmol, 51%. ^1^H NMR (400.1 MHz, DMSO-*d*_6_): δ = 27.6, 11.4, 11.2, 5.31, 3.5 (18-c-6) ppm. μ_eff_ = 3.7 μ_B_ (Evans method, ^19^F NMR, CH_2_Cl_2_/PhCF_3_, 23 °C). Material for elemental analysis was prepared by the recrystallization of crude and crown ether-free material from a DMA solution upon vapor diffusion of Et_2_O. Calculated analysis for C_34_H_36_N_5_O_10_NiK (K[NiL^OCH2O^]·DMA): C, 52.86%; H, 4.70%; N, 9.07%. Found: C, 51.74%; H, 5.09%; N, 9.49%.

### Synthesis of **[K(18-crown-6)][ZnL^OCH2O^]**

In the glovebox, a scintillation vial was charged with **H**_**3**_**L**^**OCH2O**^ (0.100 g, 0.169 mmol) and KH (0.027 g, 0.68 mmol, 4 equiv). DMA (2 mL) was added, and the effervescent solution was stirred for 1 h, during which time bubbling ceased. This solution was filtered through Celite to remove residual KH and then added to solid Zn(OAc)_2_ (0.047 g, 0.25 mmol, 1.5 equiv) and stirred for 12 h. After filtering through Celite and washing the filter cake with additional DMA (4 mL), Et_2_O was added to the filtrate to precipitate the desired complex. This suspension was stirred for 30 min and then filtered through a fine-porosity fritted funnel. The crude complex remaining on the funnel was washed five times with a 5:1 Et_2_O/DMA solution. The resulting colorless residue was then dissolved in a solution of 18-crown-6 (0.054 g, 0.20 mmol, 1.2 equiv) in CH_2_Cl_2_ (2 mL) and stirred for several minutes before filtering through Celite. The filtrate was dried *in vacuo* and then taken up in a minimum amount of MeCN. Storage in a glovebox freezer at −35 °C for 3 days yielded colorless crystals, which were harvested and washed with Et_2_O to remove a small amount of cocrystallized 18-crown-6. The crystals were redissolved in CH_2_Cl_2_ and then dried *in vacuo* at 60 °C to yield **[K(18-crown-6)][ZnL**^**OCH2O**^**]** that is free from residual MeCN. Yield: 0.063 g, 0.066 mmol, 39%. ^1^H NMR (400.1 MHz, DMSO-*d*_6_): δ = 6.62 (d, *J* = 2 Hz, 6H, *o*-Ar), 6.33 (t, *J* = 2 Hz, 3H, *p*-Ar), 6.04 (d, *J* = 7 Hz, 3H, −OC*H*_2_O−), 5.36 (d, *J* = 7 Hz, 3H, −OC*H*_2_O−), 3.53 (s, 24H, 18-c-6), 3.39 (t, *J* = 7 Hz, 6H, Zn–NC*H*_2_CH_2_N), 2.68 (t, *J* = 7 Hz, 6H, Zn–NCH_2_C*H*_2_N) ppm. ^13^C{^1^H} NMR (125.8 MHz, DMSO-*d*_6_): δ = 174.26, 156.27, 145.52, 111.09, 108.96, 91.95, 69.38, 49.99, 42.97 ppm. Material for elemental analysis was prepared by recrystallization of crude and crown ether-free material from a DMA solution upon vapor diffusion of Et_2_O. C_34_H_36_N_5_O_10_ZnK (K[ZnL^OCH2O^]·DMA): C, 52.49%; H, 4.66%; N, 8.99%. Found: C, 51.73%; H, 4.94%; N, 8.99%.

### Synthesis of **[K(18-crown-6)][CoL^OMe^]**

In the glovebox, a scintillation vial was charged with **H**_**3**_**L**^**OMe**^ (1.00 g, 1.57 mmol) and KH (0.201 g, 5.01 mmol, 4 equiv). DMA (8 mL) was added, and the effervescent solution was stirred for 1 h, during which time bubbling ceased. This solution was filtered through Celite to remove residual KH and then added to solid Co(OAc)_2_ (0.305 g, 1.72 mmol, 1.5 equiv) and stirred for 3 h. After filtering through Celite, 18-crown-6 (0.455 g, 1.72 mmol, 1.2 equiv) was added to the solution, which was then layered with Et_2_O and allowed to stand overnight, resulting in crystallization of the desired complex. The green crystals were harvested and washed with a 5:1 Et_2_O:DMA solution. Two additional rounds of crystallization from a DMA solution layered with Et_2_O were carried out with the obtained crystals being washed with 5:1 Et_2_O:DMA each time. After the third recrystallization, the green crystals were washed with Et_2_O to remove residual DMA and then dried *in vacuo* to yield analytically pure **[K(18-crown-6)][CoL**^**OMe**^**]**. Yield: 0.777 g, 0.780 mmol, 50%. ^1^H NMR (400.1 MHz, DMSO-*d*_6_): δ = 13.5, 3.6 (18-c-6), 0.0, −4.3 ppm. μ_eff_ = 4.2 μ_B_ (Evans method, ^19^F NMR, CH_2_Cl_2_/PhCF_3_, 23 °C). Calculated combustion analysis for C_45_H_63_N_4_O_15_CoK: C, 54.15%; H, 6.36%; N, 5.61%. Found: C, 54.36%; H, 6.47%; N, 5.59%.

### Synthesis of **[K(18-crown-6)][NiL^OMe^]**

In the glovebox, a scintillation vial was charged with **H**_**3**_**L**^**OMe**^ (1.0 g, 1.57 mmol) and KH (0.201 g, 5.01 mmol, 4 equiv). DMA (8 mL) was added, and the effervescent solution was stirred for 1 h, during which time bubbling ceased. This solution was filtered through Celite to remove residual KH, and then added to solid Ni(OAc)_2_ (0.304 g, 1.72 mmol, 1.5 equiv) and stirred for 3 h. After filtering through Celite, 18-crown-6 (0.455 g, 1.72 mmol, 1.2 equiv) was added to the solution, which was then layered with Et_2_O and allowed to stand overnight, resulting in the crystallization of the desired complex. The pink-orange crystals were harvested and washed with a 5:1 Et_2_O:DMA solution. An additional round of crystallization from a DMA solution layered with Et_2_O, followed by washing the crystals with 5:1 Et_2_O:DMA, was carried out. The obtained crystals were washed with Et_2_O to remove residual DMA and then dried *in vacuo* to yield **[K(18-crown-6)][NiL**^**OMe**^**]**. Yield: 0.656 g, 0.659 mmol, 42%. ^1^H NMR (400.1 MHz, DMSO-*d*_6_): δ = 21.4, 9.2, 6.4, 3.5 (18-c-6) ppm. μ_eff_ = 3.3 μ_B_ (Evans method, ^19^F NMR, CH_2_Cl_2_/PhCF_3_, 23 °C). Calculated combustion analysis for C_45_H_63_N_4_O_15_NiK: C, 54.17%; H, 6.36%; N, 5.62%. Found: C, 53.42%; H, 6.25%; N, 5.50%.

### Synthesis of **[K(18-crown-6)][ZnL^OMe^]**

In the glovebox, a scintillation vial was charged with **H**_**3**_**L**^**OMe**^ (0.200 g, 0.314 mmol) and KH (0.050 g, 1.2 mmol, 4 equiv). DMA (2 mL) was added, and the effervescent solution was stirred for 1 h, during which time bubbling ceased. This solution was filtered through Celite to remove residual KH and then added to solid Zn(OAc)_2_ (0.086 g, 0.47 mmol, 1.5 equiv) and stirred for 3 h. After the mixture was filtered through Celite, Et_2_O was added to precipitate the desired complex. This suspension was stirred for 30 min and then filtered through a fine-porosity fritted funnel. The crude complex remaining on the funnel was washed five times with a 5:1 Et_2_O/DMA solution. The resulting colorless residue was then dissolved in a solution of 18-crown-6 (0.099 g, 0.38 mmol, 1.2 equiv) in CH_2_Cl_2_ (2 mL) and stirred for several minutes before filtering through Celite. The filtrate was dried *in vacuo* and then taken up in a minimum amount of MeCN. Storage in a glovebox freezer at −35 °C for 3 days yielded colorless crystals which were harvested together and washed with Et_2_O to remove a small amount of cocrystallized 18-crown-6. The crystals were redissolved in CH_2_Cl_2_ and then dried *in vacuo* at 60 °C to yield **[K(18-crown-6)][ZnL**^**OMe**^**]** that contains no acetonitrile. Yield: 0.107 g, 0.107 mmol, 34%. ^1^H NMR (500 MHz, DMSO-*d*_6_): δ = 6.42 (s, 6H, *o*-Ar), 6.16 (s, 3H, *p*-Ar), 3.54 (s, 24H, 18-c-6), 3.42 (s, 18H, −OC*H*_3_), 3.30 (br s, 6H, Zn–NC*H*_2_CH_2_N), 2.61 (br s, 6H, Zn–NCH_2_C*H*_2_N) ppm. ^13^C{^1^H} NMR (128.7 MHz, DMSO-*d*_6_): δ = 171.84, 159.40, 144.46, 104.12, 101.05, 69.48, 55.12, 54.43, 42.13 ppm. Calculated combustion analysis: calcd for C_45_H_63_N_4_O_15_ZnK: C, 53.80%; H, 6.32%; N, 5.58%. Found: C, 53.24%; H, 6.41%; N, 5.42%.

## Supporting Information Available

Nuclear magnetic resonance data for all newly reported compounds; additional UV–visible absorption spectroscopy data; fitting of all electron paramagnetic resonance spectra; additional cyclic voltammetry data; details of crystallographic data collection and refinement; and details of density functional theory calculations (PDF)
Cartesian coordinates for all geometrically relaxed structures obtained from density functional theory calculations (XYZ)
Co(HCN)L^OCH2O^ relaxed surface scan (XYZ)
Co(MeCN)L^OCH2O^ relaxed surface scan (XYZ)

## Supplementary Material

ic3c01335_si_001.pdf

ic3c01335_si_002.xyz

ic3c01335_si_003.xyz

ic3c01335_si_004.xyz

## References

[ref1] Cook S. A.; Borovik A. S. Molecular Designs for Controlling the Local Environments around Metal Ions. Acc. Chem. Res. 2015, 48 (8), 2407–2414. 10.1021/acs.accounts.5b00212. 26181849 PMC5097670

[ref2] Drover M. W. A Guide to Secondary Coordination Sphere Editing. Chem. Soc. Rev. 2022, 51 (6), 1861–1880. 10.1039/D2CS00022A. 35188514

[ref3] Van Stappen C.; Deng Y.; Liu Y.; Heidari H.; Wang J.-X.; Zhou Y.; Ledray A. P.; Lu Y. Designing Artificial Metalloenzymes by Tuning of the Environment beyond the Primary Coordination Sphere. Chem. Rev. 2022, 122 (14), 11974–12045. 10.1021/acs.chemrev.2c00106. 35816578 PMC10199331

[ref4] Bouhadir G.; Bourissou D. Complexes of Ambiphilic Ligands: Reactivity and Catalytic Applications. Chem. Soc. Rev. 2016, 45 (4), 1065–1079. 10.1039/C5CS00697J. 26567634

[ref5] Khusnutdinova J. R.; Milstein D. Metal–Ligand Cooperation. Angew. Chem., Int. Ed. 2015, 54 (42), 12236–12273. 10.1002/anie.201503873. 26436516

[ref6] Cook S. A.; Hill E. A.; Borovik A. S. Lessons from Nature: A Bio-Inspired Approach to Molecular Design. Biochemistry 2015, 54 (27), 4167–4180. 10.1021/acs.biochem.5b00249. 26079379 PMC5097671

[ref7] MacBeth C. E.; Golombek A. P.; Young J.; Yang C.; Kuczera K.; Hendrich M. P.; Borovik A. S. O_2_ Activation by Nonheme Iron Complexes: A Monomeric Fe(III)-Oxo Complex Derived from O_2_. Science 2000, 289 (5481), 938–941. 10.1126/science.289.5481.938. 10937994

[ref8] Park Y. J.; Matson E. M.; Nilges M. J.; Fout A. R. Exploring Mn–O Bonding in the Context of an Electronically Flexible Secondary Coordination Sphere: Synthesis of a Mn(III)–Oxo. Chem. Commun. 2015, 51 (25), 5310–5313. 10.1039/C4CC08603A. 25745671

[ref9] Nichols A. W.; Hooe S. L.; Kuehner J. S.; Dickie D. A.; Machan C. W. Electrocatalytic CO_2_ Reduction to Formate with Molecular Fe(III) Complexes Containing Pendent Proton Relays. Inorg. Chem. 2020, 59 (9), 5854–5864. 10.1021/acs.inorgchem.9b03341. 32324404

[ref10] Wilson A. D.; Shoemaker R. K.; Miedaner A.; Muckerman J. T.; DuBois D. L.; DuBois M. R. Nature of Hydrogen Interactions with Ni(II) Complexes Containing Cyclic Phosphine Ligands with Pendant Nitrogen Bases. Proc. Natl. Acad. Sci. U. S. A. 2007, 104 (17), 6951–6956. 10.1073/pnas.0608928104. 17360385 PMC1855379

[ref11] Lucas R. L.; Zart M. K.; Mukherjee J.; Sorrell T. N.; Powell D. R.; Borovik A. S. A Modular Approach toward Regulating the Secondary Coordination Sphere of Metal Ions: Differential Dioxygen Activation Assisted by Intramolecular Hydrogen Bonds. J. Am. Chem. Soc. 2006, 128 (48), 15476–15489. 10.1021/ja063935+. 17132015

[ref12] Lacy D. C.; Park Y. J.; Ziller J. W.; Yano J.; Borovik A. S. Assembly and Properties of Heterobimetallic Co^II/III^/Ca^II^ Complexes with Aquo and Hydroxo Ligands. J. Am. Chem. Soc. 2012, 134 (42), 17526–17535. 10.1021/ja304525n. 22998407 PMC3638877

[ref13] Blacquiere J. M.; Pegis M. L.; Raugei S.; Kaminsky W.; Forget A.; Cook S. A.; Taguchi T.; Mayer J. M. Synthesis and Reactivity of Tripodal Complexes Containing Pendant Bases. Inorg. Chem. 2014, 53 (17), 9242–9253. 10.1021/ic5013389. 25105991

[ref14] Yeh C.-Y.; Chang C. J.; Nocera D. G. Hangman” Porphyrins for the Assembly of a Model Heme Water Channel. J. Am. Chem. Soc. 2001, 123 (7), 1513–1514. 10.1021/ja003245k. 11456732

[ref15] Creutz S. E.; Peters J. C. Exploring Secondary-Sphere Interactions in Fe–N_x_H_y_ Complexes Relevant to N_2_ Fixation. Chem. Sci. 2017, 8 (3), 2321–2328. 10.1039/C6SC04805F. 28451336 PMC5363375

[ref16] West N. M.; Miller A. J. M.; Labinger J. A.; Bercaw J. E. Homogeneous Syngas Conversion. Coord. Chem. Rev. 2011, 255 (7), 881–898. 10.1016/j.ccr.2010.08.019.

[ref17] Kita M. R.; Miller A. J. M. Cation-Modulated Reactivity of Iridium Hydride Pincer-Crown Ether Complexes. J. Am. Chem. Soc. 2014, 136 (41), 14519–14529. 10.1021/ja507324s. 25275727

[ref18] Figueroa J. S.; Melnick J. G.; Parkin G. Reactivity of the Metal→BX_3_ Dative σ-Bond: 1,2-Addition Reactions of the Fe→BX_3_ Moiety of the Ferraboratrane Complex [κ^4^-B(mim^Bu-*t*^)_3_]Fe(CO)_2_. Inorg. Chem. 2006, 45 (18), 7056–7058. 10.1021/ic061353n. 16933903

[ref19] Cowie B. E.; Emslie D. J. H. Bis-Hydrocarbyl Platinum(II) Ambiphilic Ligand Complexes: Alkyl–Aryl Exchange between Platinum and Boron. Organometallics 2015, 34 (12), 2737–2746. 10.1021/om501269x.

[ref20] Drover M. W.; Bowes E. G.; Dufour M. C.; Lesperance-Nantau L. A. Platinum Complexes of a Boron-Rich Diphosphine Ligand. Dalton. Trans. 2020, 49 (45), 16312–16318. 10.1039/D0DT00963F. 32432301

[ref21] Kiernicki J. J.; Zeller M.; Szymczak N. K. Hydrazine Capture and N–N Bond Cleavage at Iron Enabled by Flexible Appended Lewis Acids. J. Am. Chem. Soc. 2017, 139 (50), 18194–18197. 10.1021/jacs.7b11465. 29227655 PMC5955611

[ref22] Zhang R.; Warren J. J. Controlling the Oxygen Reduction Selectivity of Asymmetric Cobalt Porphyrins by Using Local Electrostatic Interactions. J. Am. Chem. Soc. 2020, 142 (31), 13426–13434. 10.1021/jacs.0c03861. 32706247

[ref23] Azcarate I.; Costentin C.; Robert M.; Savéant J.-M. Through-Space Charge Interaction Substituent Effects in Molecular Catalysis Leading to the Design of the Most Efficient Catalyst of CO_2_-to-CO Electrochemical Conversion. J. Am. Chem. Soc. 2016, 138 (51), 16639–16644. 10.1021/jacs.6b07014. 27976580

[ref24] Martin D. J.; Mayer J. M. Oriented Electrostatic Effects on O_2_ and CO_2_ Reduction by a Polycationic Iron Porphyrin. J. Am. Chem. Soc. 2021, 143 (30), 11423–11434. 10.1021/jacs.1c03132. 34292718

[ref25] Burns K. T.; Marks W. R.; Cheung P. M.; Seda T.; Zakharov L. N.; Gilbertson J. D. Uncoupled Redox-Inactive Lewis Acids in the Secondary Coordination Sphere Entice Ligand-Based Nitrite Reduction. Inorg. Chem. 2018, 57 (16), 9601–9610. 10.1021/acs.inorgchem.8b00032. 29608297 PMC6102076

[ref26] Breslow R.; Dong S. D. Biomimetic Reactions Catalyzed by Cyclodextrins and Their Derivatives. Chem. Rev. 1998, 98 (5), 1997–2011. 10.1021/cr970011j. 11848956

[ref27] Wieser C.; Dieleman C. B.; Matt D. Calixarene and Resorcinarene Ligands in Transition Metal Chemistry. Coord. Chem. Rev. 1997, 165, 93–161. 10.1016/S0010-8545(97)90153-3.

[ref28] Gramage-Doria R.; Armspach D.; Matt D. Metallated Cavitands (Calixarenes, Resorcinarenes, Cyclodextrins) with Internal Coordination Sites. Coord. Chem. Rev. 2013, 257, 776–816. 10.1016/j.ccr.2012.10.006.

[ref29] Le Poul N.; Le Mest Y.; Jabin I.; Reinaud O. Supramolecular Modeling of Mono-Copper Enzyme Active Sites with Calix[6]Arene-Based Funnel Complexes. Acc. Chem. Res. 2015, 48 (7), 2097–2106. 10.1021/acs.accounts.5b00152. 26103534

[ref30] Csicsery S. M. Shape-Selective Catalysis in Zeolites. Zeolites 1984, 4 (3), 202–213. 10.1016/0144-2449(84)90024-1.

[ref31] Wei J. Nonlinear Phenomena in Zeolite Diffusion and Reaction. Ind. Eng. Chem. Res. 1994, 33 (10), 2467–2472. 10.1021/ie00034a031.

[ref32] Kulprathipanja S., Ed.; Zeolites in Industrial Separation and Catalysis; Wiley: 2010 10.1002/9783527629565.

[ref33] Sanders J. K. M. Supramolecular Catalysis in Transition. Chem.—Eur. J. 1998, 4 (8), 1378–1383. 10.1002/(SICI)1521-3765(19980807)4:8<1378::AID-CHEM1378>3.0.CO;2-3.

[ref34] Breslow R.; Overman L. E. Artificial Enzyme” Combining a Metal Catalytic Group and a Hydrophobic Binding Cavity. J. Am. Chem. Soc. 1970, 92 (4), 1075–1077. 10.1021/ja00707a062. 5451011

[ref35] Hofmeister G. E.; Hahn F. E.; Pedersen S. F. Chiral Recognition in the Synthesis of Dimetalla-4-*tert*-butylcalix[8]arene Complexes. The Incorporation of a Metal-Alkoxide Ligand into a Molecular Cavity. J. Am. Chem. Soc. 1989, 111 (6), 2318–2319. 10.1021/ja00188a064.

[ref36] Zanotti-Gerosa A.; Solari E.; Giannini L.; Floriani C.; Re N.; Chiesi-Villa A.; Rizzoli C. Titanium-Carbon Functionalities on an Oxo Surface Defined by a Calix[4]Arene Moiety and Its Redox Chemistry. Inorg. Chim. Acta 1998, 270 (1), 298–311. 10.1016/S0020-1693(97)05863-5.

[ref37] Iwamoto H.; Yukimasa Y.; Fukazawa Y. Synthesis and Binding Behavior of a Zn(II)-Porphyrin Having Calix[5]Arene Cap. Tetrahedron Lett. 2002, 43 (45), 8191–8194. 10.1016/S0040-4039(02)01655-6.

[ref38] Loeber C.; Matt D.; De Cian A.; Fischer J. Multifunctional Phosphane and Phosphane Oxide Ligands Derived from *p*-*tert*-butylcalix[4]arene. Synthesis of a Large Diphosphane with C_2_ Symmetry and Behaving as a *Cis* or *Trans* Binding Ligand. J. Organomet. Chem. 1994, 475 (1), 297–305. 10.1016/0022-328X(94)84035-0.

[ref39] Zeng X.; Coquière D.; Alenda A.; Garrier E.; Prangé T.; Li Y.; Reinaud O.; Jabin I. Efficient Synthesis of Calix[6]Tmpa: A New Calix[6]Azacryptand with Unique Conformational and Host–Guest Properties. Chem.—Eur. J. 2006, 12 (24), 6393–6402. 10.1002/chem.200600278. 16823788

[ref40] Izzet G.; Zeitouny J.; Akdas-Killig H.; Frapart Y.; Ménage S.; Douziech B.; Jabin I.; Le Mest Y.; Reinaud O. Dioxygen Activation at a Mononuclear Cu(I) Center Embedded in the Calix[6]Arene-Tren Core. J. Am. Chem. Soc. 2008, 130 (29), 9514–9523. 10.1021/ja8019406. 18576623

[ref41] Zahim S.; Wickramasinghe L. A.; Evano G.; Jabin I.; Schrock R. R.; Müller P. Calix[6]Azacryptand Ligand with a Sterically Protected Tren-Based Coordination Site for Metal Ions. Org. Lett. 2016, 18 (7), 1570–1573. 10.1021/acs.orglett.6b00410. 26999005

[ref42] Miller D. L.; Lu C. C. Encapsulating Zinc(II) within a Hydrophobic Cavity. Dalton. Trans. 2012, 41, 7464–7466. 10.1039/c2dt30529a. 22628009

[ref43] Cain M. F.; Forrest W. P.; Peryshkov D. V.; Schrock R. R.; Müller P. Synthesis of a TREN in Which the Aryl Substituents Are Part of a 45 Atom Macrocycle. J. Am. Chem. Soc. 2013, 135 (41), 15338–15341. 10.1021/ja408964g. 24074292

[ref44] Moran J. R.; Karbach S.; Cram D. J. Cavitands: Synthetic Molecular Vessels. J. Am. Chem. Soc. 1982, 104 (21), 5826–5828. 10.1021/ja00385a064.

[ref45] Tunstad L. M.; Tucker J. A.; Dalcanale E.; Weiser J.; Bryant J. A.; Sherman J. C.; Helgeson R. C.; Knobler C. B.; Cram D. J. Host-Guest Complexation. 48. Octol Building Blocks for Cavitands and Carcerands. J. Org. Chem. 1989, 54 (6), 1305–1312. 10.1021/jo00267a015.

[ref46] Attempts to directly form **H**_**3**_**L**^**OH**^ from 3 equiv of 3,5-dihydroxybenzoic acid with TREN using various amide bond-forming coupling reagents consistently led to either incomplete conversion or intractable mixtures.

[ref47] Martí-Centelles V.; Pandey M. D.; Burguete M. I.; Luis S. V. Macrocyclization Reactions: The Importance of Conformational, Configurational, and Template-Induced Preorganization. Chem. Rev. 2015, 115 (16), 8736–8834. 10.1021/acs.chemrev.5b00056. 26248133

[ref48] Cram D. J.; Karbach S.; Kim H. E.; Knobler C. B.; Maverick E. F.; Ericson J. L.; Helgeson R. C. Host-Guest Complexation. 46. Cavitands as Open Molecular Vessels Form Solvates. J. Am. Chem. Soc. 1988, 110 (7), 2229–2237. 10.1021/ja00215a037.

[ref49] Kane C. M.; Ugono O.; Barbour L. J.; Holman T. K. Many Simple Molecular Cavitands Are Intrinsically Porous (Zero-Dimensional Pore) Materials. Chem. Mater. 2015, 27 (21), 7337–7354. 10.1021/acs.chemmater.5b02972.

[ref50] Yang L.; Powell D. R.; Houser R. P. Structural Variation in Copper(I) Complexes with Pyridylmethylamide Ligands: Structural Analysis with a New Four-Coordinate Geometry Index, τ_4_. J. Chem. Soc. Dalton. Trans. 2007, (9), 955–964. 10.1039/B617136B. 17308676

[ref51] The crystallographically independent nickel complex bearing the smaller N–Ni–N angle exhibits disorder of one equatorial N and the axial N atoms, and accordingly the angle of 130.09(12)° should be interpreted with caution.

[ref52] Schulte K. A.; Vignesh K. R.; Dunbar K. R. Effects of Coordination Sphere on Unusually Large Zero Field Splitting and Slow Magnetic Relaxation in Trigonally Symmetric Molecules. Chem. Sci. 2018, 9 (48), 9018–9026. 10.1039/C8SC02820F. 30647894 PMC6301199

[ref53] As ascertained from a search of the Cambridge Structural Database, version 5.43 (updated September 2022).

[ref54] Alliger G. E.; Müller P.; Do L. H.; Cummins C. C.; Nocera D. G. Family of Cofacial Bimetallic Complexes of a Hexaanionic Carboxamide Cryptand. Inorg. Chem. 2011, 50 (9), 4107–4115. 10.1021/ic200143b. 21446665

[ref55] Pinkowicz D.; Birk F. J.; Magott M.; Schulte K.; Dunbar K. R. Systematic Study of Open-Shell Trigonal Pyramidal Transition-Metal Complexes with a Rigid-Ligand Scaffold. Chem.—Eur. J. 2017, 23 (15), 3548–3552. 10.1002/chem.201605528. 28055144

[ref56] Ray M.; Yap G. P. A.; Rheingold A. L.; Borovik A. S. Synthesis and Characterization of a Trigonal Monopyramidal Nickel(II) Complex. J. Chem. Soc. Chem. Commun. 1995, 1777–1778. 10.1039/c39950001777.

[ref57] Warr R. J.; Westra A. N.; Bell K. J.; Chartres J.; Ellis R.; Tong C.; Simmance T. G.; Gadzhieva A.; Blake A. J.; Tasker P. A.; Schröder M. Selective Extraction and Transport of the [PtCl_6_]^2–^ Anion through Outer-Sphere Coordination Chemistry. Chem.—Eur. J. 2009, 15 (19), 4836–4850. 10.1002/chem.200802377. 19370745

[ref58] Ray M.; Hammes B. S.; Yap G. P. A.; Rheingold A. L.; Borovik A. S. Structure and Physical Properties of Trigonal Monopyramidal Iron(II), Cobalt(II), Nickel(II), and Zinc(II) Complexes. Inorg. Chem. 1998, 37 (7), 1527–1532. 10.1021/ic970831e.

[ref59] Cummins C. C.; Lee J.; Schrock R. R.; Davis W. D. Trigonal-Monopyramidal M^III^ Complexes of the Type [M(N_3_N)] (M = Ti, V, Cr, Mn, Fe; N_3_N = [(*t*BuMe_2_Si)NCH_2_CH_2_]_3_N). Angew. Chem., Int. Ed. 1992, 31 (11), 1501–1503. 10.1002/anie.199215011.

[ref60] Alliger G. E.; Müller P.; Cummins C. C.; Nocera D. G. Cofacial Dicobalt Complex of a Binucleating Hexacarboxamide Cryptand Ligand. Inorg. Chem. 2010, 49 (8), 3697–3699. 10.1021/ic100395a. 20337489

[ref61] Weberg A. B.; McCollom S. P.; Thierer L. M.; Gau M. R.; Carroll P. J.; Tomson N. C. Using Internal Electrostatic Fields to Manipulate the Valence Manifolds of Copper Complexes. Chem. Sci. 2021, 12 (12), 4395–4404. 10.1039/D0SC06364A. 34163703 PMC8179517

[ref62] Stauber J. M.; Alliger G. E.; Nocera D. G.; Cummins C. C. Second-Coordination-Sphere Assisted Selective Colorimetric Turn-on Fluoride Sensing by a Mono-Metallic Co(II) Hexacarboxamide Cryptand Complex. Inorg. Chem. 2017, 56 (14), 7615–7619. 10.1021/acs.inorgchem.7b01335. 28665117

[ref63] Jones M. B.; MacBeth C. E. Tripodal Phenylamine-Based Ligands and Their Co^II^ Complexes. Inorg. Chem. 2007, 46 (20), 8117–8119. 10.1021/ic701289y. 17764176

[ref64] Kalra A.; Bagchi V.; Paraskevopoulou P.; Das P.; Ai L.; Sanakis Y.; Raptopoulos G.; Mohapatra S.; Choudhury A.; Sun Z.; Cundari T. R.; Stavropoulos P. Is the Electrophilicity of the Metal Nitrene the Sole Predictor of Metal-Mediated Nitrene Transfer to Olefins? Secondary Contributing Factors as Revealed by a Library of High-Spin Co(II) Reagents. Organometallics 2021, 40 (12), 1974–1996. 10.1021/acs.organomet.1c00267. 35095166 PMC8797515

[ref65] West R. J.; Lincoln S. F. Exchange of Acetonitrile on Complexes of Nickel(II) and Cobalt(II) Formed with 2,2’,2”-Triaminotriethylamine, and 2,2’,2”-Tri(N,N-Dimethylamino)Triethylamine. Inorg. Chem. 1973, 12 (2), 494–497. 10.1021/ic50120a059.

[ref66] Handy N. C.; Cohen A. J. Left-Right Correlation Energy. Mol. Phys. 2001, 99 (5), 403–412. 10.1080/00268970010018431.

[ref67] The OLYP functional was chosen given its typically good ability to properly determine the relative energies of different electronic spin states for open-shell transition-metal complexes, coupled with its lower computational expense in comparison to hybrid DFT functionals. For example, see Verma P.; Varga Z.; Klein J. E. M. N.; Cramer C. J.; Que L.; Truhlar D. G. Assessment of Electronic Structure Methods for the Determination of the Ground Spin States of Fe(II), Fe(III) and Fe(IV) Complexes. Phys. Chem. Chem. Phys. 2017, 19 (20), 13049–13069. 10.1039/C7CP01263B. 28484765

[ref68] A previous study found that the tripodal vanadium complexes [(Me_3_Si)NCH_2_CH_2_]_3_N)V and [(Me_3_Si)NCH_2_CH_2_]_3_N)VO similarly have shallow potential energy surfaces along the axial V–N_amine_ coordinate. See Majumdar S.; Stauber J. M.; Palluccio T. D.; Cai X.; Velian A.; Rybak-Akimova E. V.; Temprado M.; Captain B.; Cummins C. C.; Hoff C. D. Inorg. Chem. 2014, 53 (20), 11185–11196. 10.1021/ic5017005. 25280113

[ref69] Cammarota R. C.; Xie J.; Burgess S. A.; Vollmer M. V.; Vogiatzis K. D.; Ye J.; Linehan J. C.; Appel A. M.; Hoffmann C.; Wang X.; Young V. G.; Lu C. C. Thermodynamic and Kinetic Studies of H_2_ and N_2_ Binding to Bimetallic Nickel-Group 13 Complexes and Neutron Structure of a Ni(Η_2_) Adduct. Chem. Sci. 2019, 10 (29), 7029–7042. 10.1039/C9SC02018G. 31588270 PMC6676469

[ref70] Rondelez Y.; Duprat A.; Reinaud O. Calix[6]Arene-Based Cuprous “Funnel Complexes”: A Mimic for the Substrate Access Channel to Metalloenzyme Active Sites. J. Am. Chem. Soc. 2002, 124 (7), 1334–1340. 10.1021/ja0161958. 11841303

[ref71] Gutsche C. D. Calixarenes. Acc. Chem. Res. 1983, 16 (5), 161–170. 10.1021/ar00089a003.

[ref72] Gutsche C. D.; Bauer L. J. Calixarenes. 13. The Conformational Properties of Calix[4]Arenes, Calix[6]Arenes, Calix[8]Arenes, and Oxacalixarenes. J. Am. Chem. Soc. 1985, 107 (21), 6052–6059. 10.1021/ja00307a038.

[ref73] Armarego W. L. F.; Chai C. L. L. Purification of Laboratory Chemicals, 5th ed.; Elsevier, 2003.

[ref74] Dey S. K.; Das G. Fluoride Selectivity Induced Transformation of Charged Anion Complexes into Unimolecular Capsule of a π-Acidic Triamide Receptor Stabilized by Strong N–H···F– and C–H···F– Hydrogen Bonds. Cryst. Growth Des. 2011, 11 (10), 4463–4473. 10.1021/cg200679x.

