# Supplementary material for: Coordinatively Unsaturated Metallates of Cobalt(II), Nickel(II), and Zinc(II) Guarded by a Rigid and Narrow Void
Source: Inorg Chem. 2023 Jul 18;62(30):11920–31. doi: 10.1021/acs.inorgchem.3c01335 (PMC10394664; doi:10.1021/acs.inorgchem.3c01335)
Supplement: Supplementary file 1 — ic3c01335_si_001.pdf [file ic3c01335_si_001.pdf]

Supporting Information for the Paper Entitled:

Coordinatively Unsaturated Metallates of Cobalt(II), Nickel(II), and Zinc(II) Guarded by a Rigid and Narrow Void

Christopher D. Hastings, Lucy S. X. Huffman, Chandan Kumar Tiwari, Jolaine Galindo Betancourth, William W. Brennessel, and Brandon R. Barnett*

Department of Chemistry, University of Rochester, Rochester, NY 14627

Email: brandon.barnett@rochester.edu

Contents

S1. Nuclear Magnetic Resonance Spectra – S-2

S2. UV-Visible Absorption Spectroscopy Data – S-10

S3. Electron Paramagnetic Resonance Spectra – S-14!

S4. Additional Cyclic Voltammetry Data – S-16

S5. Details of Crystallographic Structure Determinations – S-18

S6 – Details of Density Functional Theory Calculations – S-37

S7. References – S-70

S1. Nuclear Magnetic Resonance Spectra

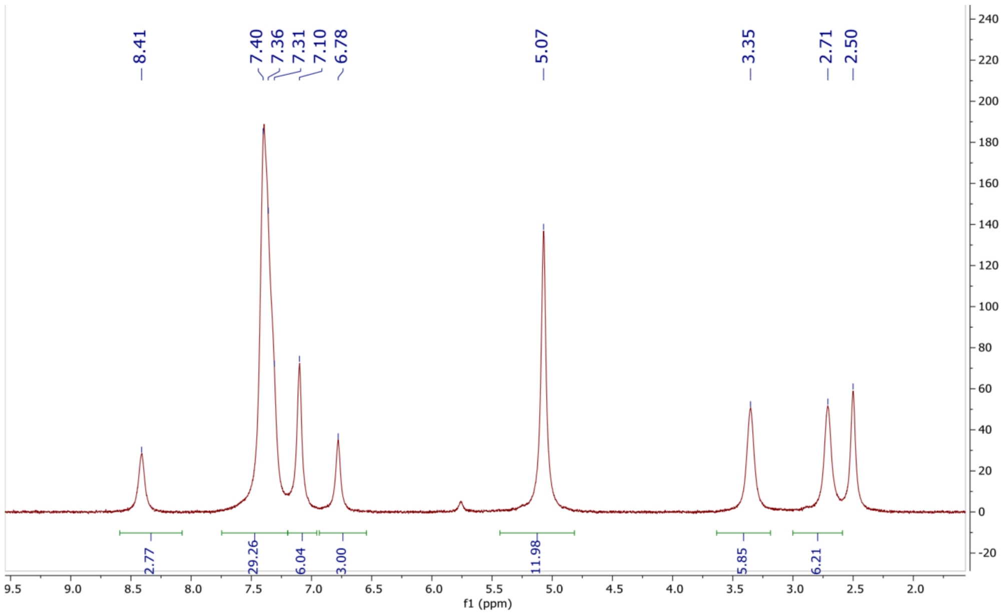

Figure S1. ^{1}H NMR spectrum (400.1 MHz, 23 ºC) of $\mathbf{H_3L^{OBn}}$. The peak at 2.50 ppm corresponds to the DMSO-d_6 solvent residual.

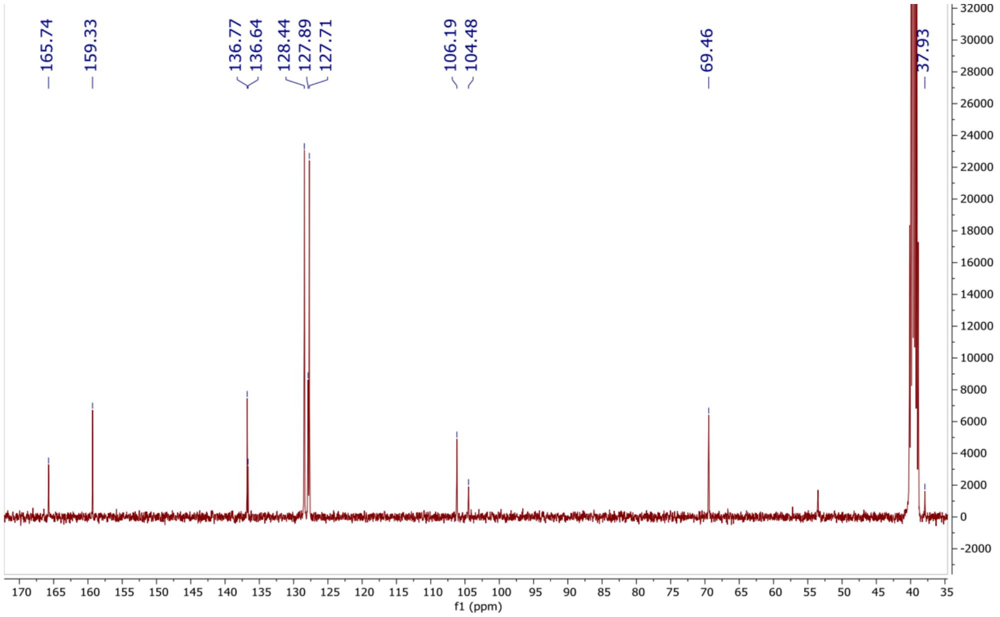

Figure S2. $^{13}C\{^{1}H\}$ NMR spectrum (125.8 MHz, 23 ºC) of $\mathbf{H_3L^{OBn}}$. The unpicked multiplet centered at 39.5 ppm corresponds to the DMSO-d_6 solvent.

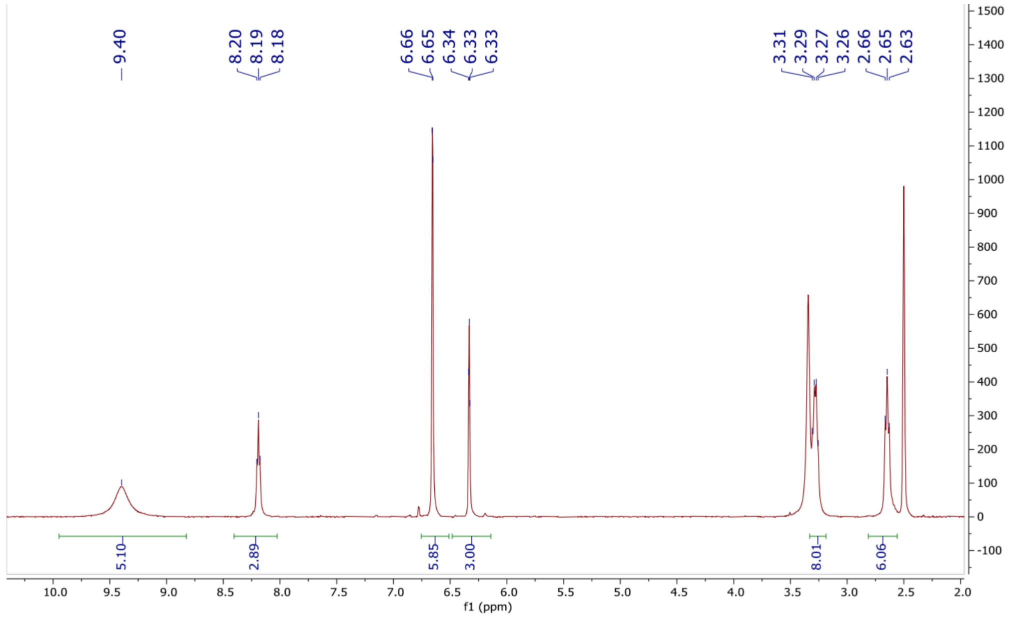

Figure S3. ^{1}H NMR spectrum (400.1 MHz, 23 ºC) of $\mathbf{H_3L^{OH}}$. The unpicked peaks at 2.50 and 3.33 ppm correspond to the DMSO-d_6 solvent residual and water, respectively.

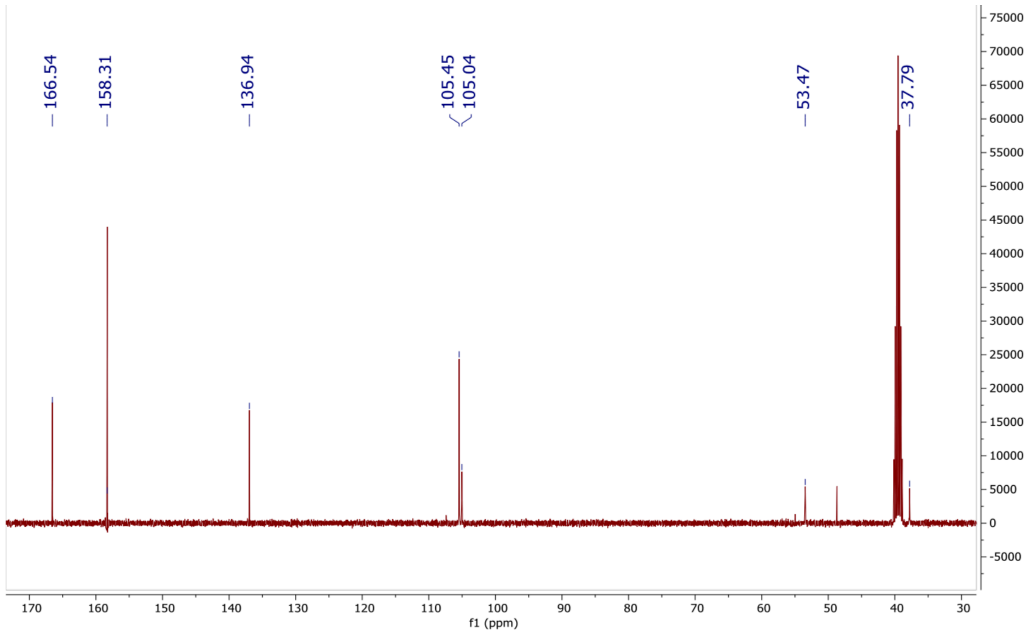

Figure S4. $^{13}C\{^{1}H\}$ NMR spectrum (125.8 MHz, 23 ºC) of $\mathbf{H_3L^{OH}}$. The unpicked multiplet centered at 39.5 ppm corresponds to the DMSO-d_6 solvent.

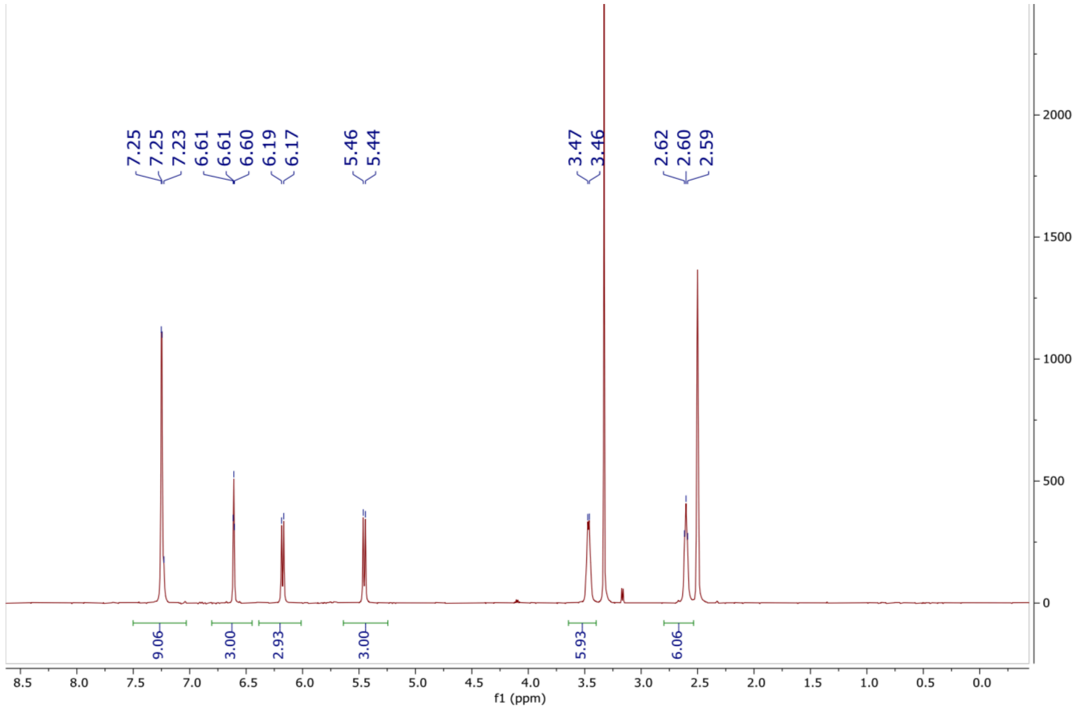

Figure S5. ^{1}H NMR spectrum (400.1 MHz, 23 ºC) of $\mathbf{H_3L^{OCH2O}}$. The unpicked peaks at 2.50 and 3.33 ppm correspond to the DMSO-d_6 solvent residual and water, respectively.

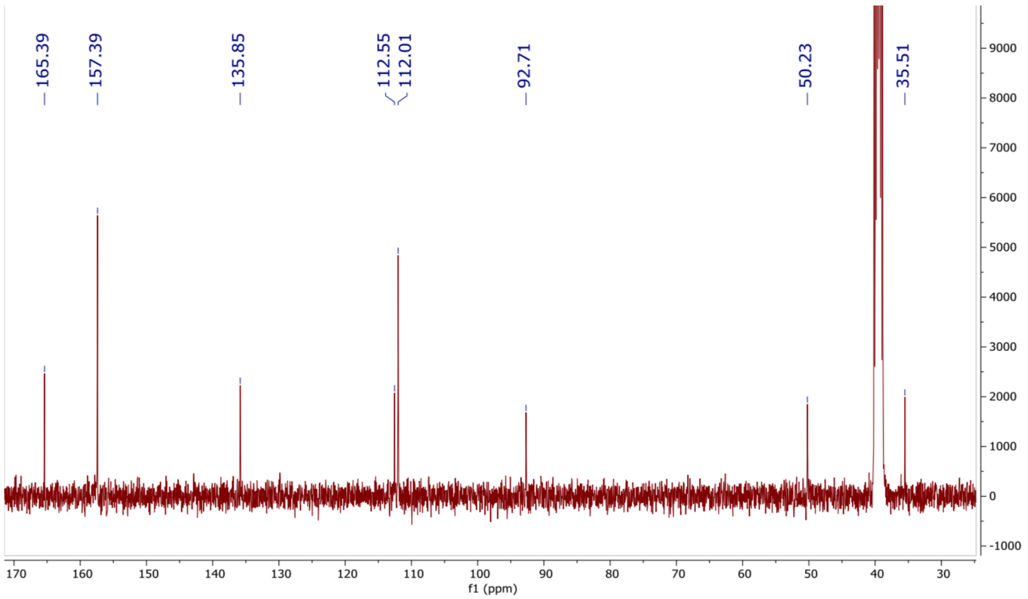

Figure S6. $^{13}C\{^{1}H\}$ NMR spectrum (125.8 MHz, 23 ºC) of $\mathbf{H_3L^{OCH2O}}$. The unpicked multiplet centered at 39.5 ppm corresponds to the DMSO-d_6 solvent.

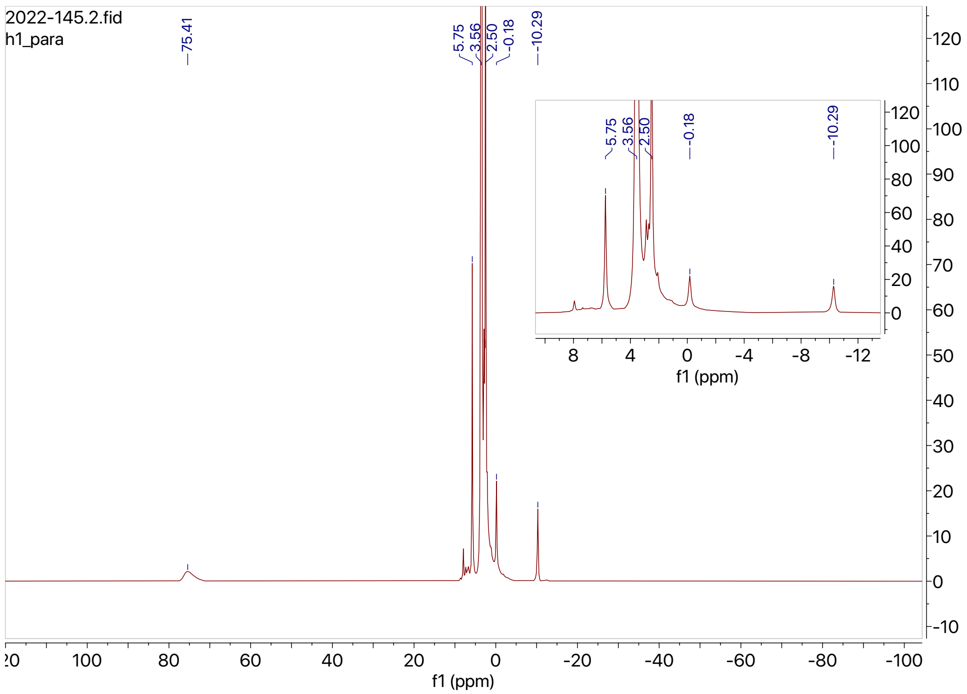

Figure S7. ^{1}H NMR spectrum (400.1 MHz, 23 ºC) of **[K(18-crown-6)][CoL^{OCH2O}]**. The unpicked peak at 2.50 ppm corresponds to the DMSO-d_6 solvent residual. The singlet at 5.75 ppm corresponds to CH_2Cl_2.

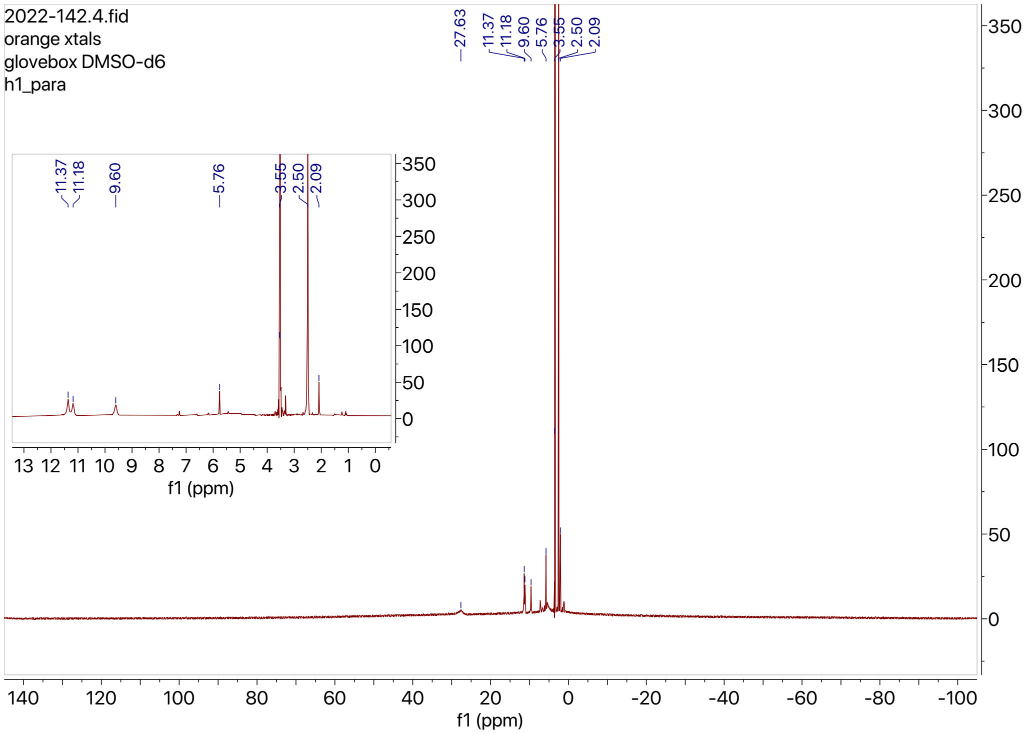

Figure S8. ^{1}H NMR spectrum (400.1 MHz, 23 ºC) of **[K(18-crown-6)][NiL^{OCH2O}]**. The unpicked peak at 2.50 ppm corresponds to the DMSO-d_6 solvent residual. The singlet at 5.76 ppm corresponds to CH_2Cl_2, while the singlet at 2.09 ppm corresponds to acetonitrile.

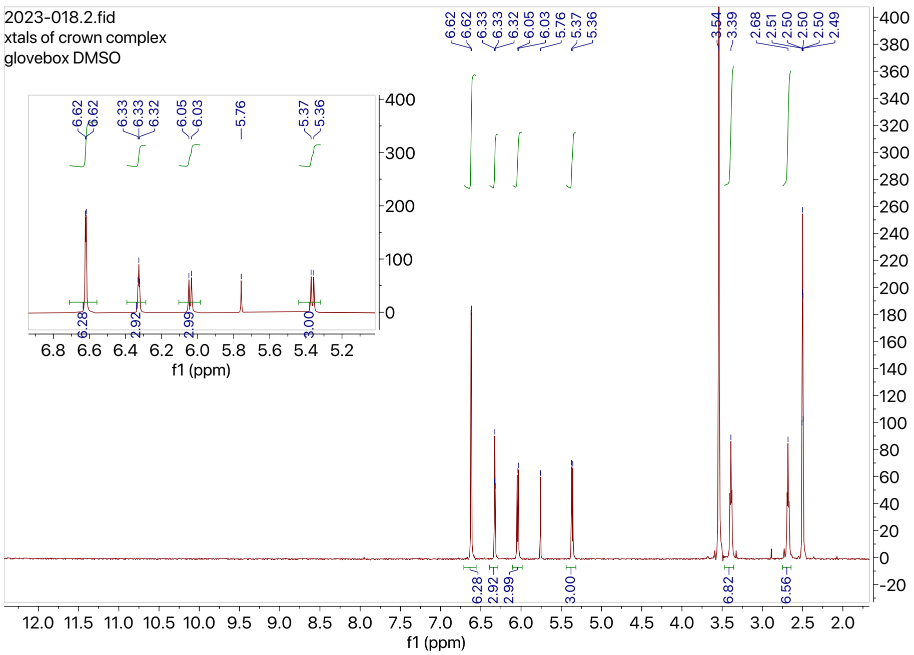

Figure S9. ^{1}H NMR spectrum (400.1 MHz, 23 ºC) of **[K(18-crown-6)][ZnL^{OCH2O}]**. The unpicked peak at 2.50 ppm corresponds to the DMSO-d_6 solvent residual. The singlet at 5.76 ppm corresponds to CH_2Cl_2.

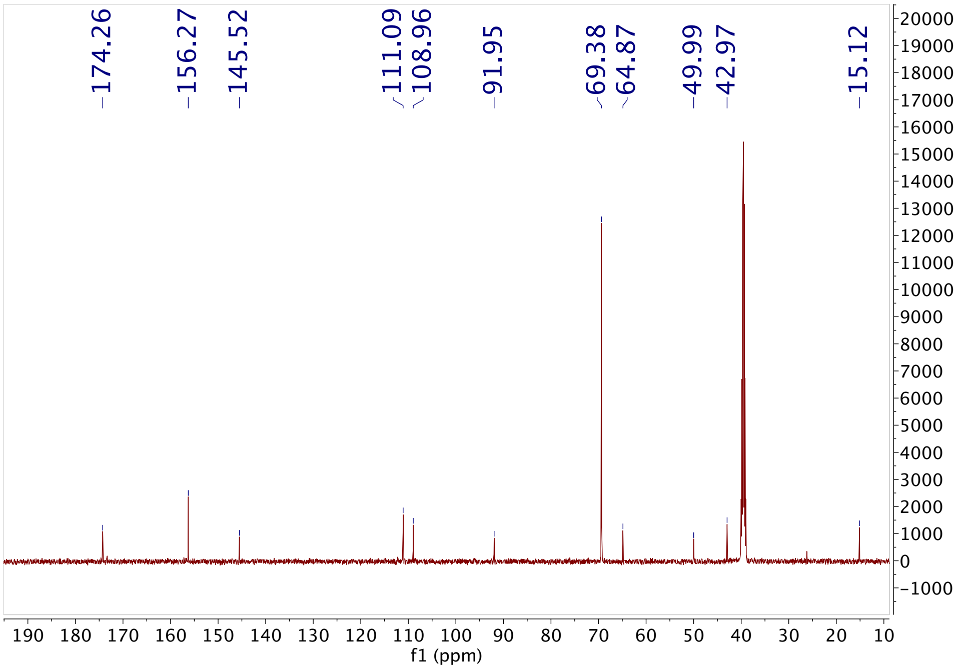

Figure S10. $^{13}C\{^{1}H\}$ NMR spectrum (125.8 MHz, 23 ºC) of **[K(18-crown-6)][ZnL^{OCH2O}]**. The unpicked multiplet centered at 39.5 ppm corresponds to the DMSO-d_6 solvent residual. The peaks at 64.9 and 15.1 ppm correspond to Et_2O.

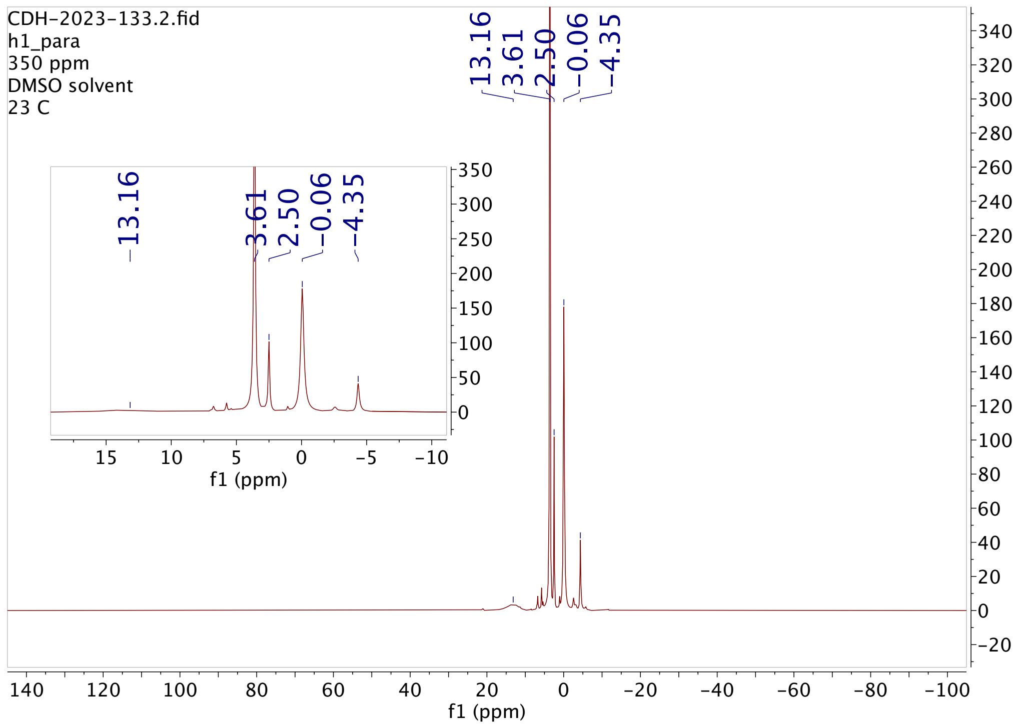

Figure S11. ^{1}H NMR spectrum (400.1 MHz, 23 ºC) of **[K(18-crown-6)][CoL^{OMe}]**. The unpicked peak at 2.50 ppm corresponds to the DMSO-d_6 solvent residual.

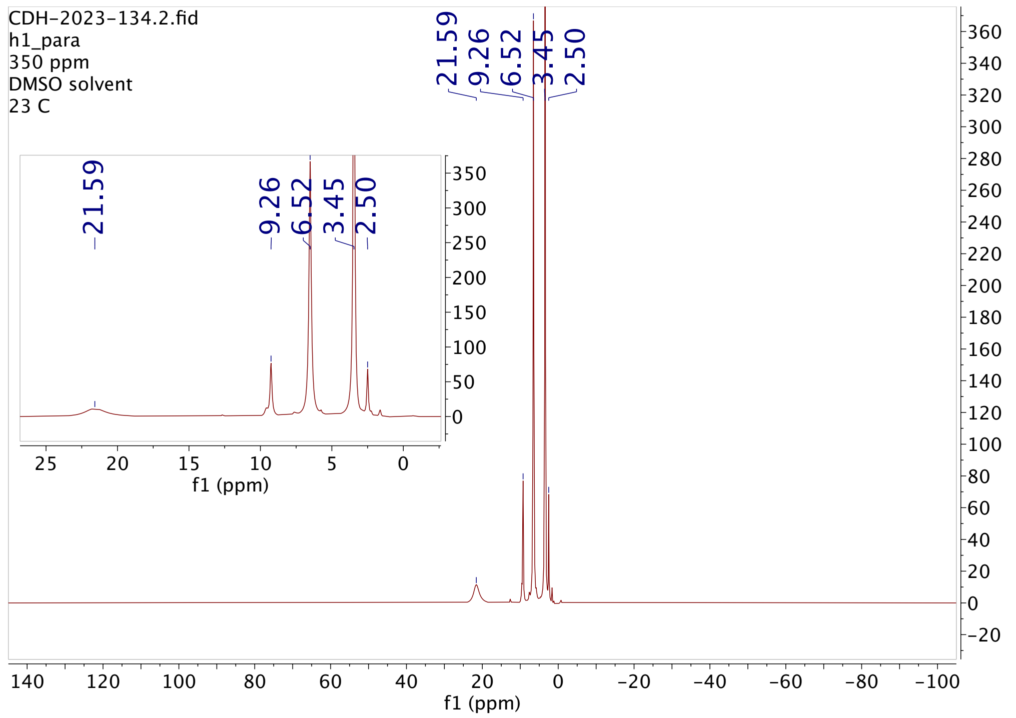

Figure S12. ^{1}H NMR spectrum (400.1 MHz, 23 ºC) of **[K(18-crown-6)][NiL^{OMe}]**. The peak at 2.50 ppm corresponds to the DMSO-d_6 solvent residual.

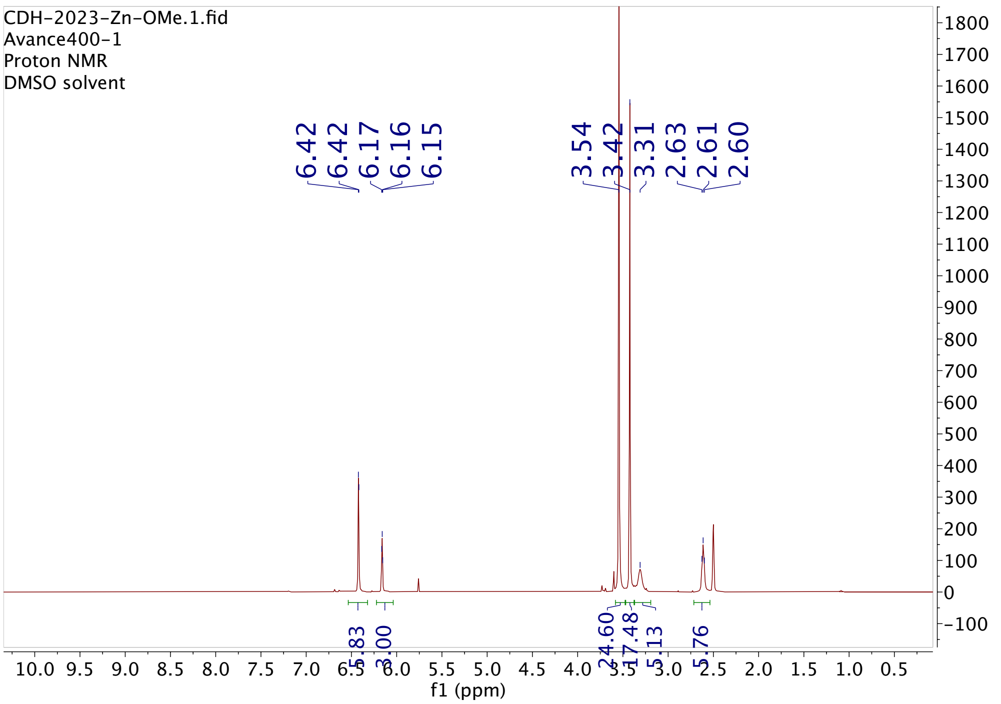

Figure S13. ^{1}H NMR spectrum (500 MHz, 23 ºC) of **[K(18-crown-6)][ZnL^{OMe}]**. The unpicked peaks at 5.74 and 2.50 ppm correspond to CH_2Cl_2 and the DMSO-d_6 solvent residual.

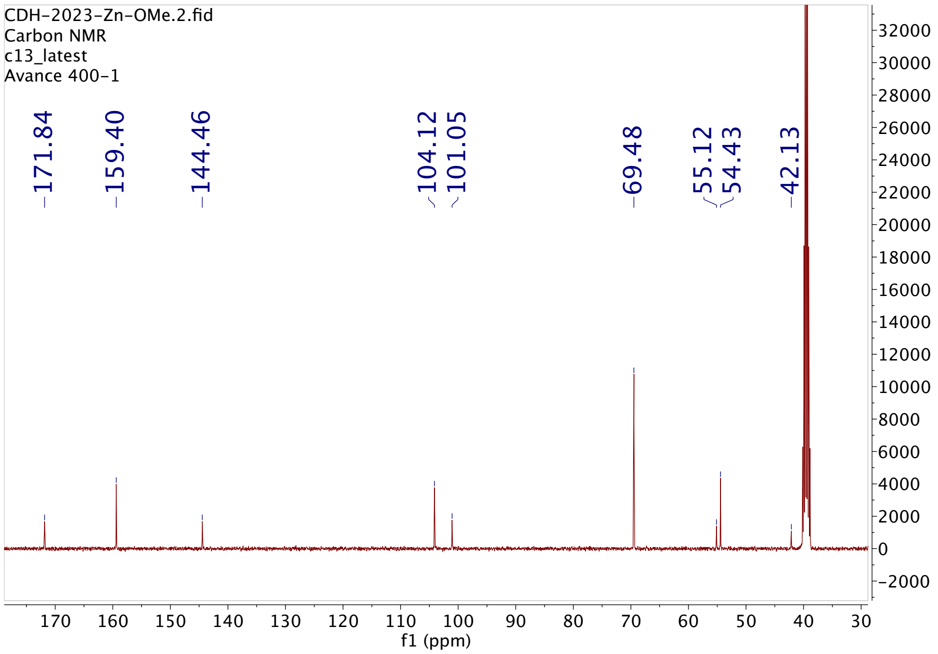

Figure S14. $^{13}C\{^{1}H\}$ NMR spectrum (125.8 MHz, 23 ºC) of **[K(18-crown-6)][ZnL^{OMe}]**. The unpicked multiplet at 39.5 ppm corresponds to the DMSO-d_6 solvent residual.

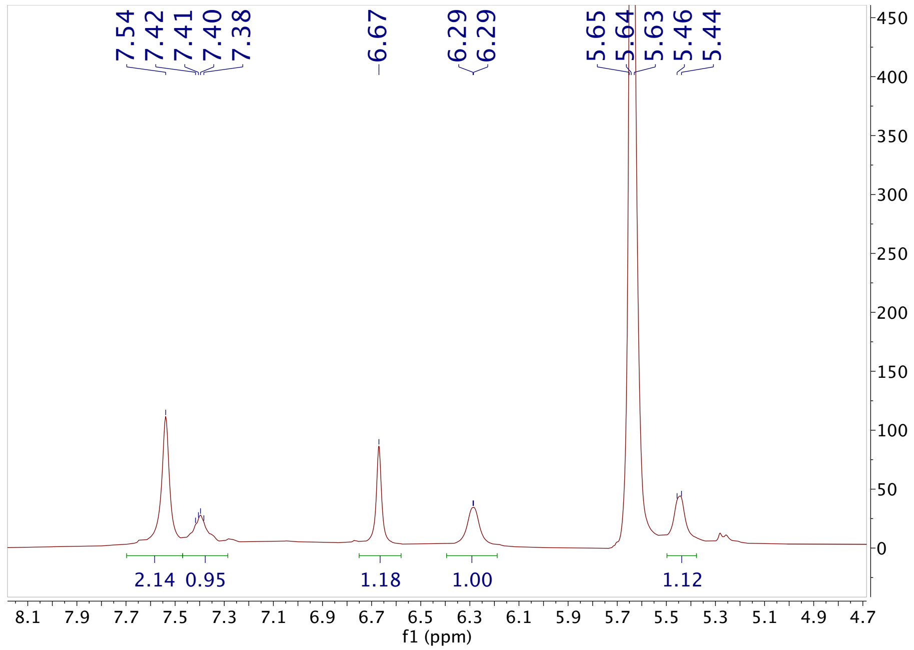

Figure S15. Partial ^{1}H NMR spectrum (500 MHz) of $\mathbf{H_3L^{OCH2O}}$ in CD_3OD at -80 ºC showing that the -OCH_2O- methylene protons (corresponding to the broad peaks at 6.29 and 5.45 ppm) do not display lowered symmetry on the NMR timescale compared to spectra collected at ambient temperature. The broadness is a result of difficulties in properly shimming at these low temperatures.

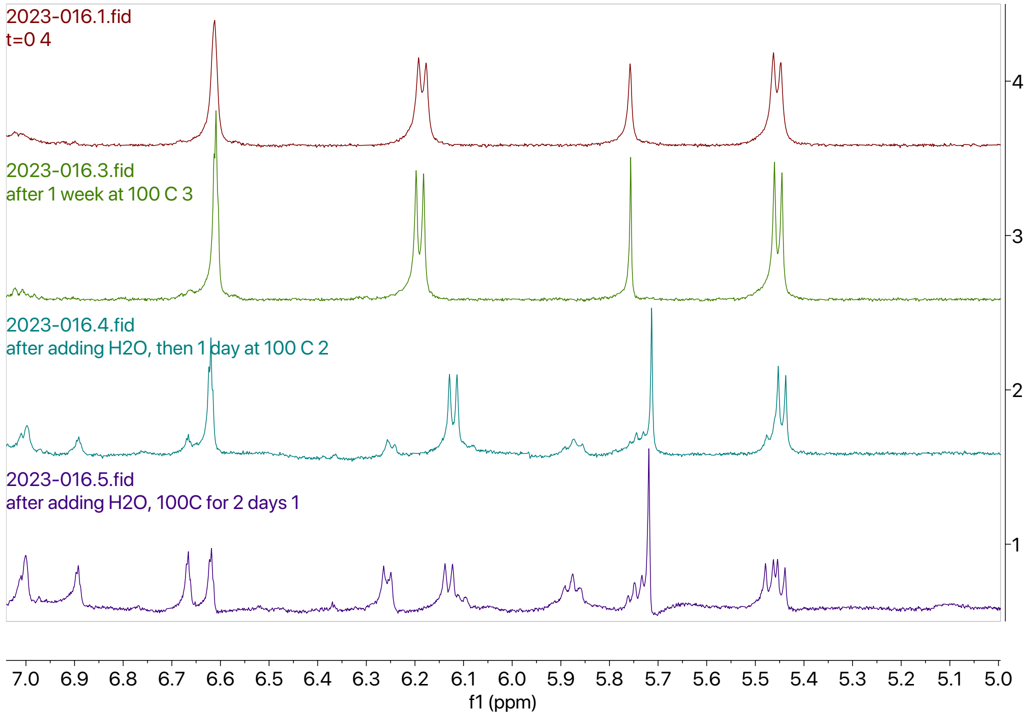

Figure S16. ^{1}H NMR spectra (500 MHz, 23 ºC) of $\mathbf{H_3L^{OCH2O}}$ in DMSO-d_6 as prepared in dried solvent (purple); after heating this solution in a J-Young NMR tube at 100 ºC for 7 days (cyan); after adding one drop of deionized water to this solution and then heating to 100 ºC for one day (green) and two days (red).

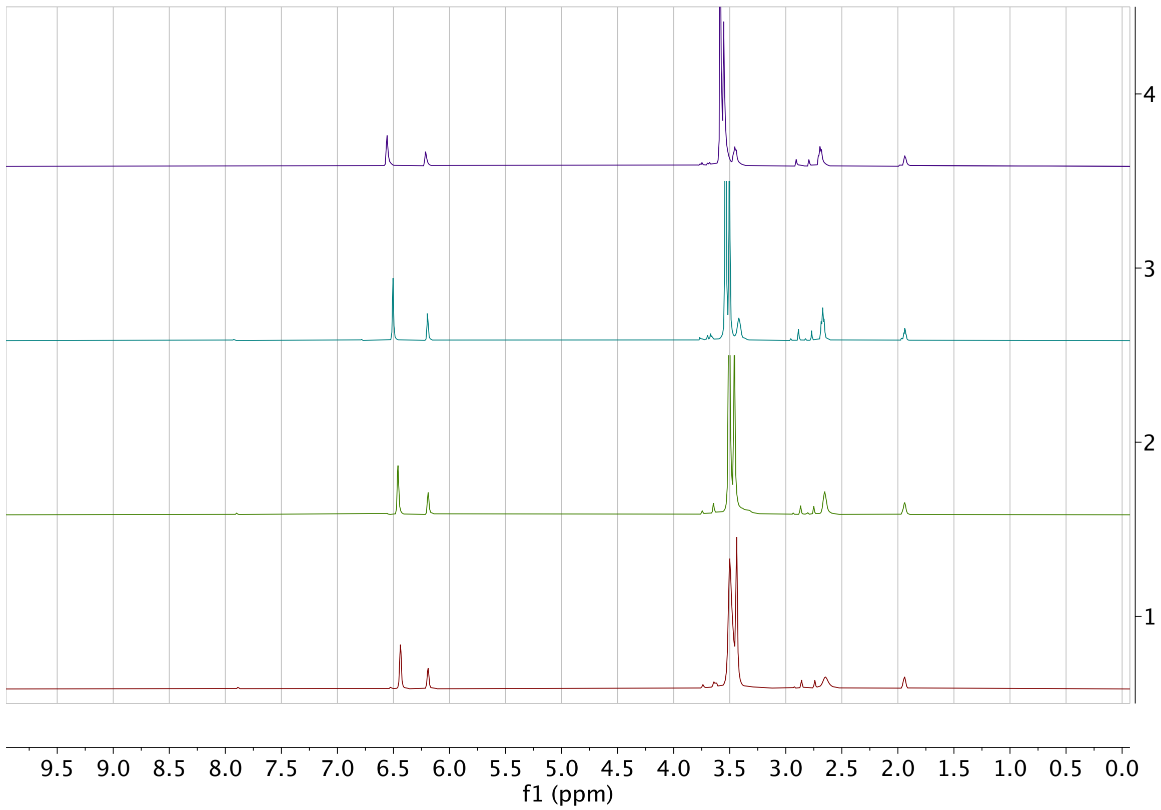

Figure S17. Variable-temperature ^{1}H NMR spectra (500 MHz) of **[K(18-crown-6)][ZnL^{OMe}]** in CD_3CN. Spectra were recorded at 75 ºC (purple), 20 ºC (blue), -20 ºC (green), and -40 ºC (red). The absence of any spectral broadening as the temperature is lowered, coupled with minimal changes in chemical shifts, suggest that acetonitrile coordination is not occurring to any appreciable extent.

S2. UV-Visible Absorption Spectroscopy Data

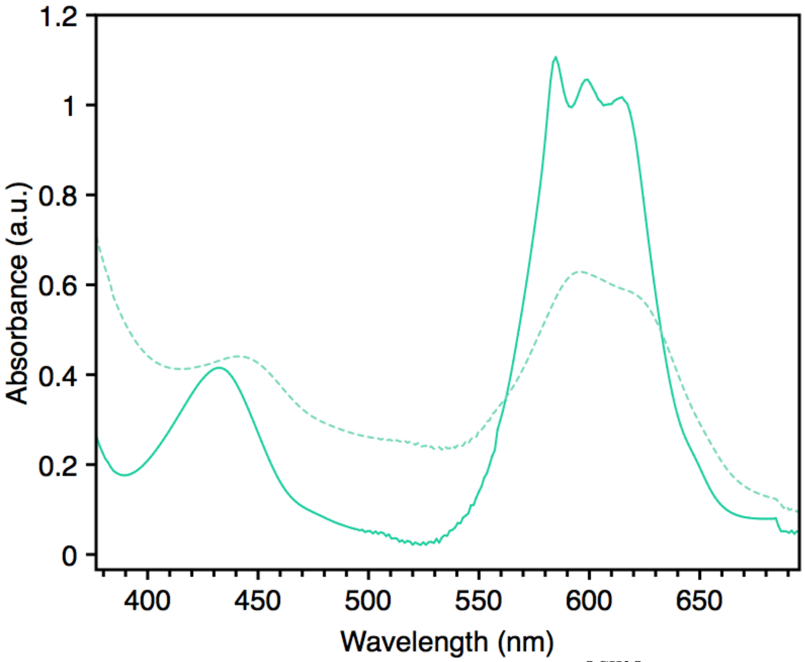

Figure S18. UV-Visible absorption spectrum of **[K(18-crown-6)][CoL^{OCH2O}]** (solid line) in DMF and **[K(18-crown-6)][CoL^{OMe}]** (dashed line) at 23 ºC in CH_2Cl_2. The sharp feature at approximately 690 nm is an instrumental artifact.

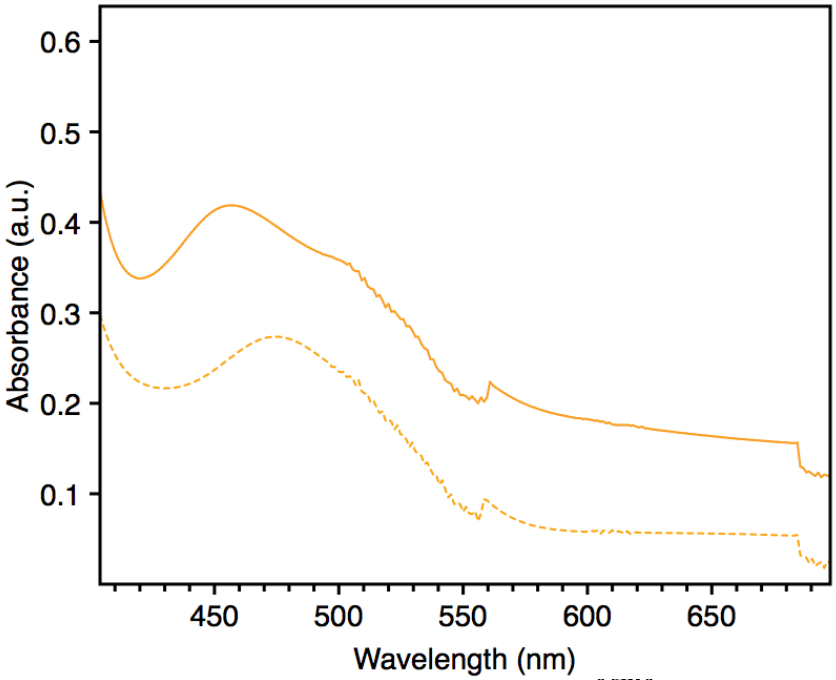

Figure S19. UV-Visible absorption spectrum of **[K(18-crown-6)][NiL^{OCH2O}]** (solid line) in DMF and **[K(18-crown-6)][NiL^{OMe}]** (dashed line) in CH_2Cl_2 at 23 ºC. The sharp features at approximately 560 and 690 nm are instrumental artifacts.

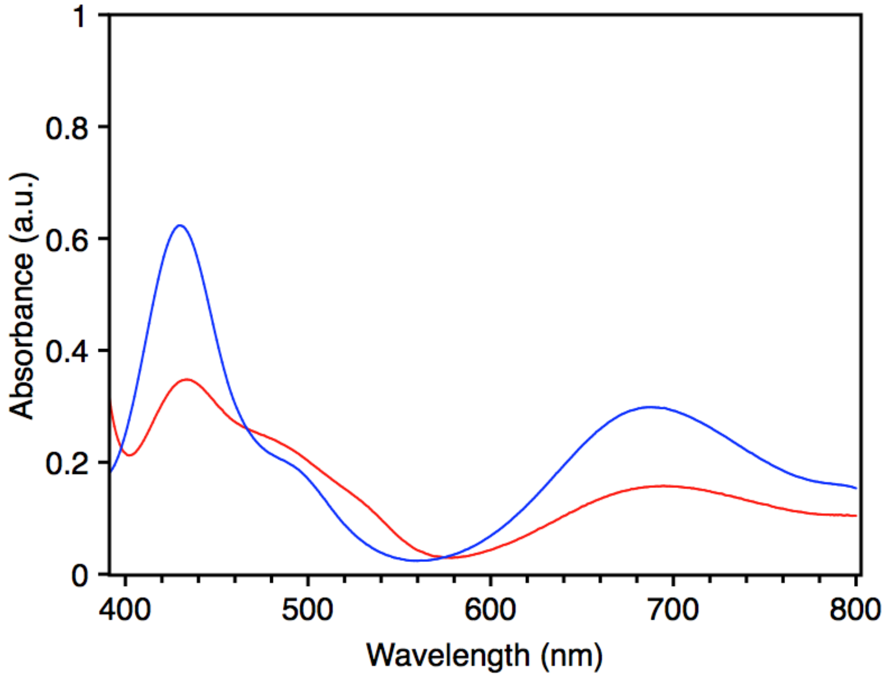

Figure S20. UV-visible spectra of **[K(18-crown-6)][NiL^{OMe}]** in acetonitrile at 23 ºC (red) and -40 ºC (blue).

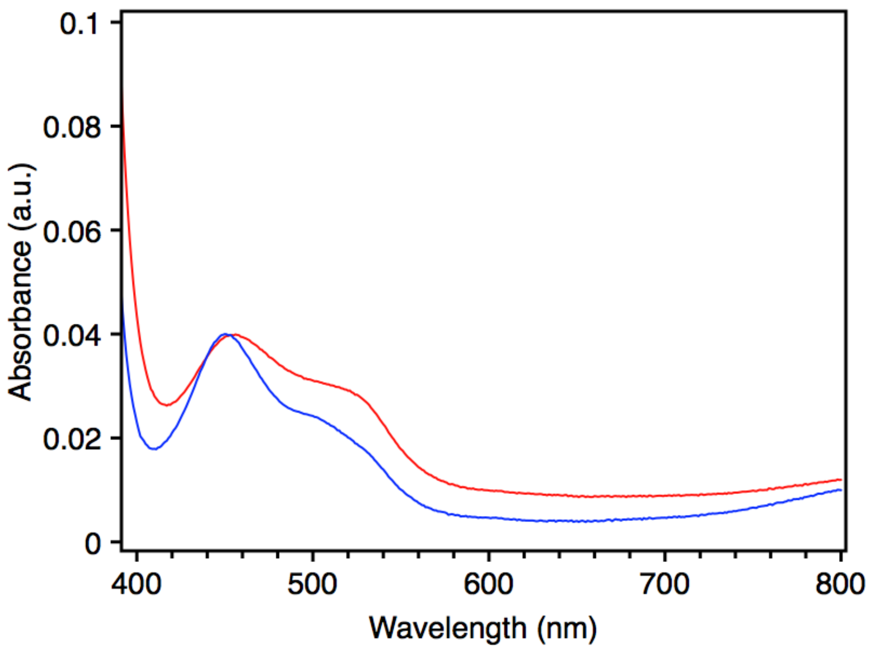

Figure S21. UV-visible spectra of **[K(18-crown-6)][NiL^{OCH2O}]** in acetonitrile at 23 ºC (red) and -40 ºC (blue).

S2.1. Van't Hoff Analysis of MeCN Binding to [CoL^{OMe}]$^{-}$. The variable-temperature spectra shown in Figure 6d were used to estimate the enthalpy change associated with this event. Given that both **[CoL^{OMe}]$^{-}$** and **[Co(NCMe)L^{OMe}]$^{-}$** are present at all accessible temperatures, the determination of extinction coefficients for UV-vis peaks is not straightforward. We decided to

use *N,N*-dimethylacetamide (DMA) to make a Beer's Law calibration curve (Figure S22), given that it does not appear to coordinate to **[CoL^{OMe}]$^-$**, as well as the fact that it has a similar dielectric constant to that of acetonitrile (ε(DMA) = 37.8 vs. ε(MeCN) = 37.5). For the feature with λ_{max} at 600 nm, this gives an extinction coefficient of 5.82•10^{-2} mM^{-1} cm^{-1}.

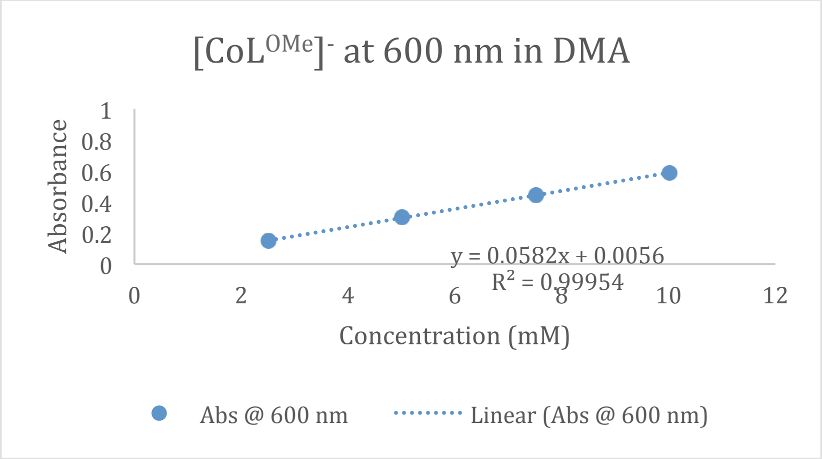

Figure S22. Beer's Law calibration plot for **[CoL^{OMe}]$^-$** (DMA, 23 ºC).

Under the assumption that the extinction coefficient for this feature is identical in MeCN, we can calculate the equilibrium constant K_{eq} for spectra at a given temperature according to Eq. 1.

$$K_{eq} = \frac{[[Co(NCMe)L^{OMe}]^-]}{[[CoL^{OMe}]^-]} \qquad (1)$$

So as to minimize the temperature range from which thermodynamic parameters (which are assumed to be independent of temperature) were extracted, we selected the temperature points between -10 ºC and 60 ºC to calculate the reaction enthalpy. Using the van't Hoff equation (Eq. 2 and Figure S23) gives Δ*H* = -5.7 kcal/mol and Δ*S* = -22.2 cal/(mol•K). We note that the enthalpy value measured here is less exothermic than the value calculated using Density Functional Theory. We posit that some of this discrepancy is attributable to the inherent error associated with assuming that the extinction coefficient is solvent independent.

$$\ln K_{eq} = \frac{-\Delta H}{RT} + \frac{\Delta S}{R} \qquad (2)$$

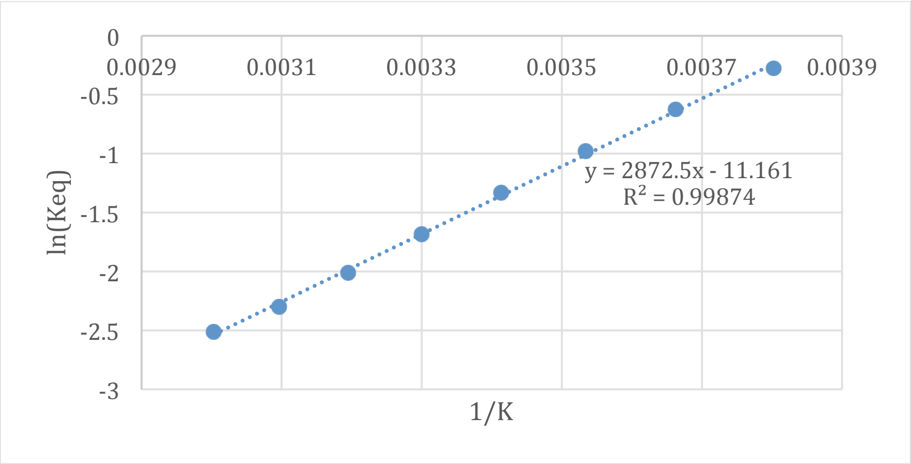

Figure S23. Van't Hoff plot for acetonitrile binding to **[CoL^{OMe}]$^-$**.

S3. Electron Paramagnetic Resonance Spectra

S3.1. General Information. Fitting of EPR spectral parameters was performed using the EasySpin[1] program.

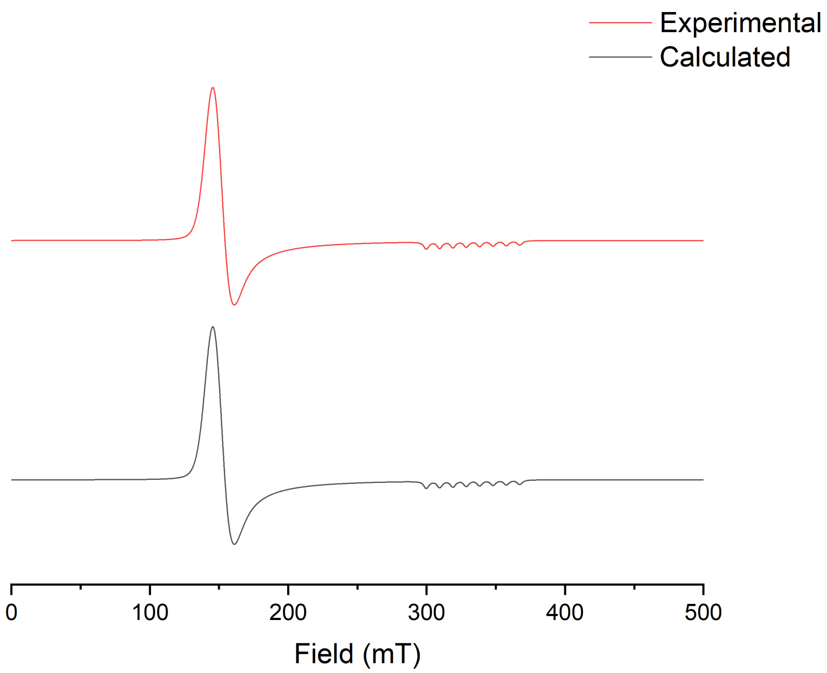

Figure S24. Comparison of experimental (red) and simulated (grey) X-band EPR spectra for **[K(18-crown-6)][CoL^{OCH2O}]** in frozen CH_2Cl_2 solution at 10 K. Simulation parameters: g_1 = 4.49; g_2 = 2.01; ^{59Co}A = 271.4 MHz; lwpp$_1$ = 1.59 mT; lwpp$_2$ = 2.00 mT; gStrain = 0.41.

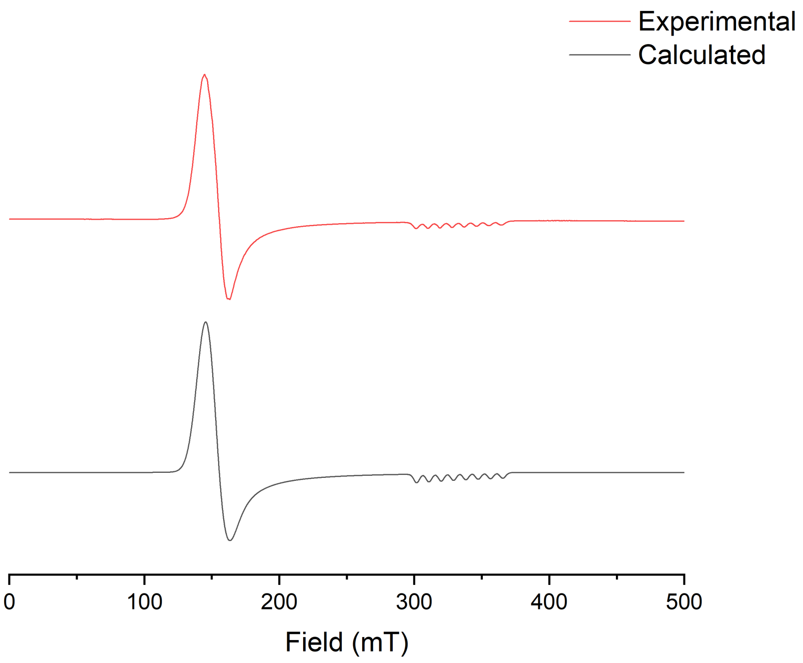

Figure S25. Comparison of experimental (red) and simulated (grey) X-band EPR spectra for **[K(18-crown-6)][CoL^{OMe}]** in frozen CH_2Cl_2 solution at 10 K. Simulation parameters: g_1 = 4.48; g_2 = 2.01; ^{59Co}A = 257.0 MHz; $lwpp_1$ = 4.00 mT; $lwpp_2$ = 0.27 mT; gStrain = 0.41.

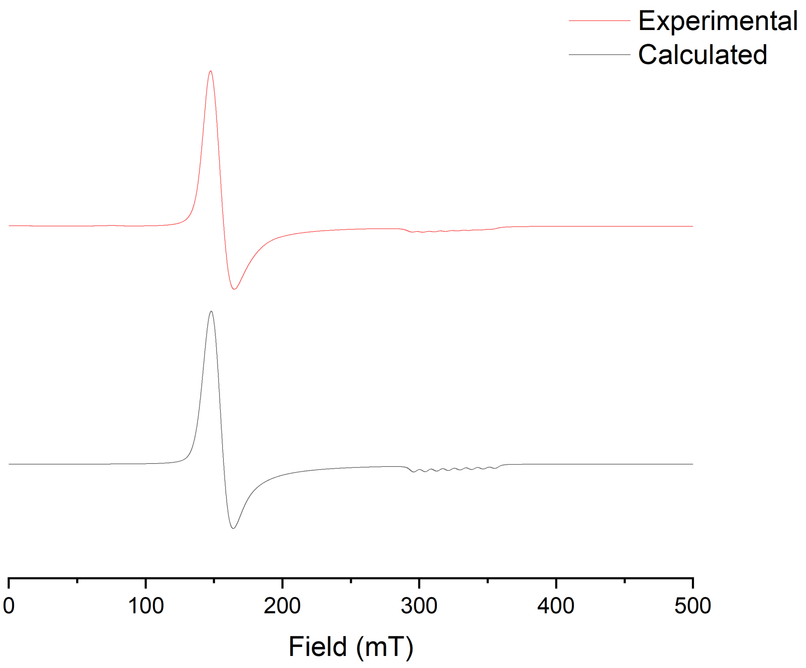

Figure S26. Comparison of experimental (red) and simulated (grey) X-band EPR spectra for **[K(18-crown-6)][CoL^{OMe}]** in frozen MeCN solution at 10 K. Simulation parameters: g_1 = 4.42; g_2 = 2.06; ^{59Co}A = 245.0 MHz; $lwpp_1$ = 3.47 mT; $lwpp_2$ = 2.17 mT; gStrain = 0.37.

S4. Additional Cyclic Voltammetry Data

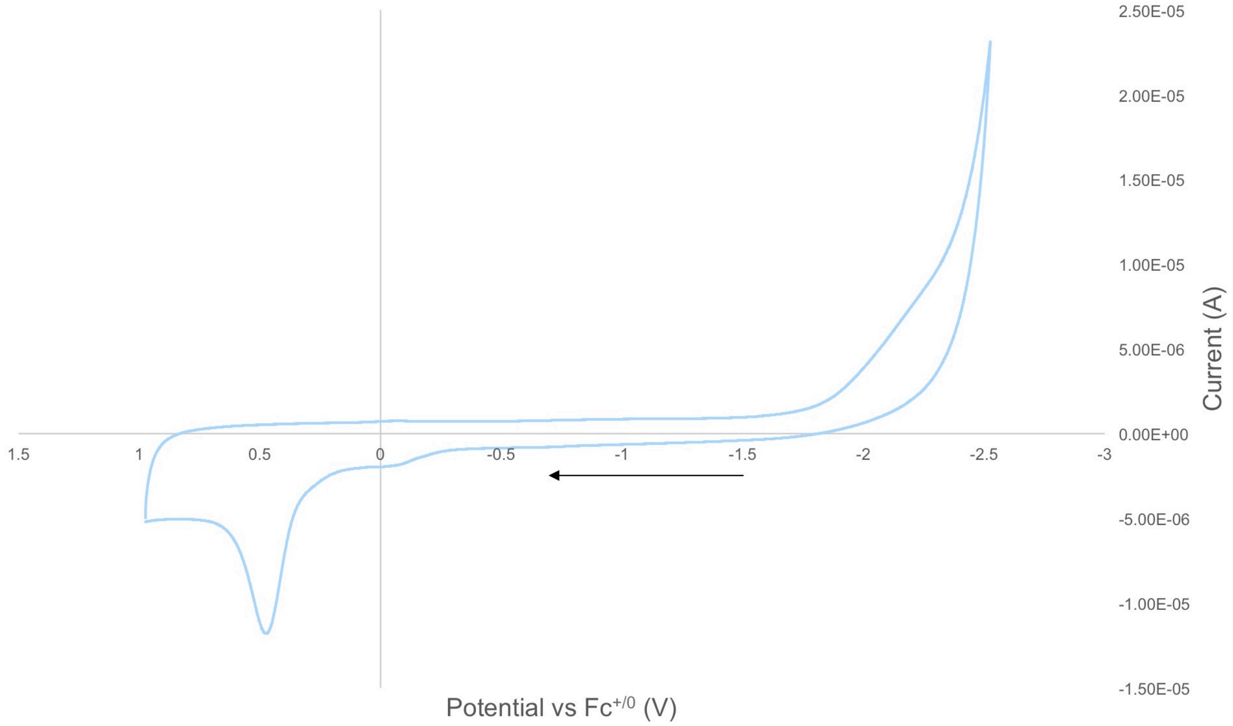

Figure S27. Voltammogram of **[K(18-crown-6)][CoL^{OCH2O}]** scanning across the entire CH_2Cl_2 solvent window. A freshly polished glassy carbon working electrode was used. Two identical scans were performed immediately preceding that shown here. Conditions: 0.1 V/s; 1 mM analyte; 1 M [(nBu)$_4$N]PF_6; anhydrous CH_2Cl_2 solvent; ambient temperature; N_2 atmosphere. Arrow denotes sweep direction. The peak seen on the anodic sweep centered at approximately -0.1 V is believed to originate from a trace, unidentified impurity.

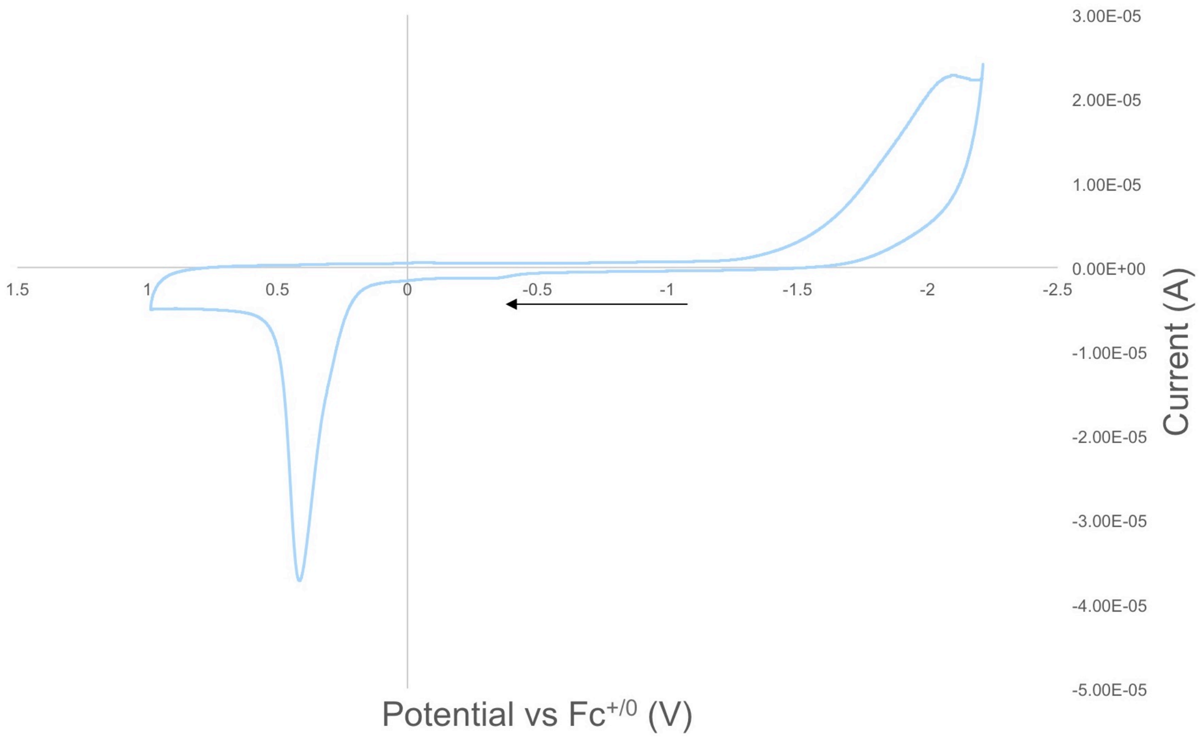

Figure S28. Voltammogram of **[K(18-crown-6)][NiL^{OCH2O}]** scanning across the entire CH_2Cl_2 solvent window. A freshly polished glassy carbon working electrode was used. Two identical scans were performed immediately preceding that shown here. Conditions: 0.1 V/s; 1 mM analyte; 1 M [(nBu)$_4$N]PF_6; anhydrous CH_2Cl_2 solvent; ambient temperature; N_2 atmosphere. Arrow denotes sweep direction.

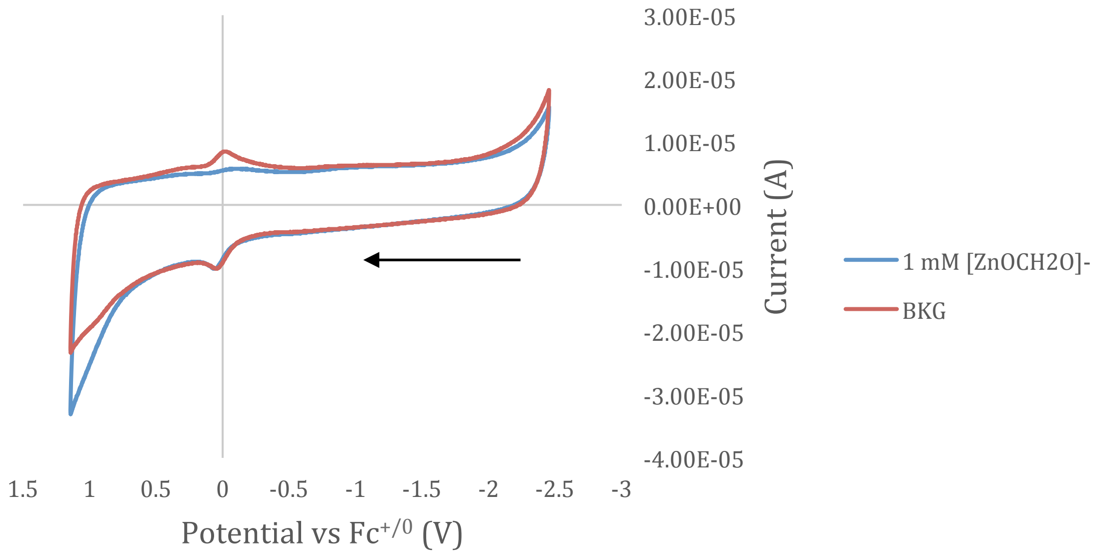

Figure S29. Voltammogram of **[K(18-crown-6)][ZnL^{OCH2O}]** scanning across the entire CH_2Cl_2 solvent window (blue line) overlaid with an identical scan of a solution without the zinc complex (red line). Both voltammograms were obtained with freshly polished glassy carbon working electrodes. Two identical scans were performed immediately preceding that shown here. Conditions: 1 V/s; 1 mM analyte; 1 M [(nBu)$_4$N]PF_6; anhydrous CH_2Cl_2 solvent; ambient temperature; N_2 atmosphere. Arrow denotes sweep direction.

S5. Details of Crystallographic Structure Determinations

S5.1. CCDC Deposition. All eight crystal structures reported herein have been deposited with the Cambridge Crystallographic Data Center (CCDC) and have been assigned the CCDC deposition numbers **2256935–2256942**.

S5.2. Details of data collection, structure solution, and refinement.

H_3L^{OCH2O}. A crystal (0.12 x 0.107 x 0.018 mm^3) was placed onto a nylon loop and mounted on a Rigaku XtaLAB Synergy-S Dualflex diffractometer equipped with a HyPix-6000HE HPC area detector for data collection at 100.01(10) K. A preliminary set of cell constants and an orientation matrix were calculated from a small sampling of reflections.[2] A short pre-experiment was run, from which an optimal data collection strategy was determined. The full data collection was carried out using a PhotonJet (Cu) X-ray source with frame times of 6.03 and 26.53 seconds and a detector distance of 34.0 mm. Series of frames were collected in 0.50º steps in ω at different 2θ, κ, and ϕ settings. After the intensity data were corrected for absorption, the final cell constants were calculated from the xyz centroids of 13026 strong reflections from the actual data collection after integration.[2] See Table S5.1 for additional crystal and refinement information.

The structure was solved using SHELXT[3] and refined using SHELXL.[4] The space group $P2_1/c$ was determined based on systematic absences. Most or all non-hydrogen atoms were assigned from the solution. Full-matrix least squares / difference Fourier cycles were performed which located any remaining non-hydrogen atoms. All non-hydrogen atoms were refined with anisotropic displacement parameters. The N-H hydrogen atoms were found from the difference Fourier map and refined freely. All other hydrogen atoms were placed in ideal positions and refined as riding atoms with relative isotropic displacement parameters. The final full matrix least squares refinement converged to $R1 = 0.0499$ (F^2, $I > 2\sigma(I)$) and $wR2 = 0.1490$ (F^2, all data). Structure solution and refinement were performed within the Olex2 program.[5]

[K(18-crown-6)][CoL^{OCH2O}]•MeCN. A crystal (0.162 x 0.145 x 0.102 mm^3) was placed onto a nylon loop and mounted on a Rigaku XtaLAB Synergy-S Dualflex diffractometer equipped with a HyPix-6000HE HPC area detector for data collection at 100.00(10) K. A preliminary set of cell constants and an orientation matrix were calculated from a small sampling of reflections.[2] A short pre-experiment was run, from which an optimal data collection strategy was determined. The full data collection was carried out using a PhotonJet (Cu) X-ray source with frame times of 1.84 and 7.36 seconds and a detector distance of 34.0 mm. Series of frames were collected in 0.50º steps in ω at different 2θ, κ, and ϕ settings. After the intensity data were corrected for absorption, the final cell constants were calculated from the xyz centroids of 32005 strong reflections from the actual data collection after integration.[2] See Table S5.2 for additional crystal and refinement information.

The structure was solved using SHELXT[3] and refined using SHELXL.[4] The space group $P2_{1/}c$ was determined based on systematic absences. Most or all non-hydrogen atoms were assigned from the solution. Full-matrix least squares / difference Fourier cycles were performed which located any remaining non-hydrogen atoms. All non-hydrogen atoms were refined with

anisotropic displacement parameters. All hydrogen atoms were placed in ideal positions and refined as riding atoms with relative isotropic displacement parameters. The final full matrix least squares refinement converged to $R1 = 0.0405$ (F^2, $I > 2\sigma(I)$) and $wR2 = 0.1014$ (F^2, all data). Structure solution and refinement were performed within the Olex2 program.[5]

The structure is the one suggested. The asymmetric unit contains one monocationic potassium complex, one monoanionic cobalt complex, and one acetonitrile solvent molecule of crystallization, all in general positions. The cations and anions are in contact and form a one-dimensional chain along [001]. The cobalt atom is out of the N2-N3-N4 plane (away from atom N1) by 0.198(1) degrees.

[K(18-crown-6)][NiL^{OCH2O}]• 0.15 MeCN. A crystal (0.223 x 0.151 x 0.126 mm^3) was placed onto a nylon loop and mounted on a Rigaku XtaLAB Synergy-S Dualflex diffractometer equipped with a HyPix-6000HE HPC area detector for data collection at 100.00(10) K. A preliminary set of cell constants and an orientation matrix were calculated from a small sampling of reflections.[2] A short pre-experiment was run, from which an optimal data collection strategy was determined. The full data collection was carried out using a PhotonJet (Cu) X-ray source with frame times of 0.21 and 0.83 seconds and a detector distance of 34.0 mm. Series of frames were collected in 0.50º steps in ω at different 2θ, κ, and ϕ settings. After the intensity data were corrected for absorption, the final cell constants were calculated from the xyz centroids of 64411 strong reflections from the actual data collection after integration.[2] See Table S5.3 for additional crystal and refinement information.

The structure was solved using SHELXT[3] and refined using SHELXL.[4] The space group *P*-1 was determined based on intensity statistics. Most or all non-hydrogen atoms were assigned from the solution. Full-matrix least squares / difference Fourier cycles were performed which located any remaining non-hydrogen atoms. All non-hydrogen atoms were refined with anisotropic displacement parameters. All hydrogen atoms were placed in ideal positions and refined as riding atoms with relative isotropic displacement parameters. The final full matrix least squares refinement converged to $R1 = 0.0442$ (F^2, $I > 2\sigma(I)$) and $wR2 = 0.1081$ (F^2, all data). Structure solution and refinement were performed within the Olex2 program.[5]

The asymmetric unit contains two [K(18-crown-6)]$^+$ cations, two monoanionic Ni complexes, and a partially occupied acetonitrile solvent molecule, all in general positions. The cations and anions alternate one-dimensionally along [1 1 -1]. The $N(CH_2CH_2N)_3$ part and one arm of the ligand coordinating Ni2 is modeled as disordered over two positions (0.73:0.27). The acetonitrile solvent molecule is of partial occupancy, disordered over two positions (0.195:0.112).

[K(18-crown-6)][ZnL^{OCH2O}]• MeCN. A crystal (0.34 x 0.128 x 0.046 mm^3) was placed onto a nylon loop and mounted on a Rigaku XtaLAB Synergy-S Dualflex diffractometer equipped with a HyPix-6000HE HPC area detector for data collection at 100.00(10) K. A preliminary set of cell constants and an orientation matrix were calculated from a small sampling of reflections.[2] A short pre-experiment was run, from which an optimal data collection strategy was determined. The full data collection was carried out using a PhotonJet (Cu) X-ray source with frame times of 0.33 and 2.00 seconds and a detector distance of 34.0 mm. Series of frames were collected in 0.50º steps in ω at different 2θ, κ, and ϕ settings. After the intensity data were corrected for absorption, the final cell constants were calculated from the xyz centroids of 45930 strong

reflections from the actual data collection after integration.[2] See Table S5.4 for additional crystal and refinement information.

The structure was solved using SHELXT[3] and refined using SHELXL.[4] The space group *Pbca* was determined based on systematic absences. Most or all non-hydrogen atoms were assigned from the solution. Full-matrix least squares / difference Fourier cycles were performed which located any remaining non-hydrogen atoms. All non-hydrogen atoms were refined with anisotropic displacement parameters. All hydrogen atoms were placed in ideal positions and refined as riding atoms with relative isotropic displacement parameters. The final full matrix least squares refinement converged to $R1 = 0.0348$ (F^2, $I > 2\sigma(I)$) and $wR2 = 0.0921$ (F^2, all data). Structure solution and refinement were performed within the Olex2 program.[5]

The asymmetric unit contains one contact cation-anion pairing and one acetonitrile solvent molecule in general positions. The crown ether and the solvent molecule are modeled as disordered over two positions each (0.76:0.24 and 0.70:0.30, respectively). Molecules are linked one-dimensionally via O…K contacts along [001].

[K(18-crown-6)][CoL$^{\mathbf{OMe}}$]. A crystal (0.484 x 0.049 x 0.029 mm^3) was placed onto a nylon loop and mounted on a Rigaku XtaLAB Synergy-S Dualflex diffractometer equipped with a HyPix-6000HE HPC area detector for data collection at 100.01(10) K. A preliminary set of cell constants and an orientation matrix were calculated from a small sampling of reflections.[2] A short pre-experiment was run, from which an optimal data collection strategy was determined. The full data collection was carried out using a PhotonJet (Cu) X-ray source with frame times of 6.40 and 25.61 seconds and a detector distance of 34.0 mm. Series of frames were collected in 0.50° steps in ω at different 2θ, κ, and ϕ settings. After the intensity data were corrected for absorption, the final cell constants were calculated from the xyz centroids of 19059 strong reflections from the actual data collection after integration.[2] See Table S5.5 for additional crystal and refinement information.

The structure was solved using SHELXT[3] and refined using SHELXL.[4] The space group $P2_1/c$ was determined based on systematic absences. Most or all non-hydrogen atoms were assigned from the solution. Full-matrix least squares / difference Fourier cycles were performed which located any remaining non-hydrogen atoms. All non-hydrogen atoms were refined with anisotropic displacement parameters. All hydrogen atoms were placed in ideal positions and refined as riding atoms with relative isotropic displacement parameters. The final full matrix least squares refinement converged to $R1 = 0.0516$ (F^2, $I > 2\sigma(I)$) and $wR2 = 0.1518$ (F^2, all data). Structure solution and refinement were performed within the Olex2 program.[5]

The asymmetric unit contains one cation-anion pairing in a general position. The alternating cations and anions are linked one-dimensionally via K…O intercations.

[K(18-crown-6)][NiL$^{\mathbf{OMe}}$]. A crystal (0.357 x 0.188 x 0.185 mm^3) was placed onto a nylon loop and mounted on a Rigaku XtaLAB Synergy-S Dualflex diffractometer equipped with a HyPix-6000HE HPC area detector for data collection at 99.99(10) K. A preliminary set of cell constants and an orientation matrix were calculated from a small sampling of reflections.[2] A short pre-experiment was run, from which an optimal data collection strategy was determined. The full data collection was carried out using a PhotonJet (Cu) X-ray source with frame times of 0.13 and 0.51 seconds and a detector distance of 34.0 mm. Series of frames were collected in

0.50° steps in ω at different 2θ, κ, and ϕ settings. After the intensity data were corrected for absorption, the final cell constants were calculated from the xyz centroids of 55085 strong reflections from the actual data collection after integration.[2] See Table S5.6 for additional crystal and refinement information.

The structure was solved using SHELXT[3] and refined using SHELXL.[4] The space group $P2_1/c$ was determined based on systematic absences. Most or all non-hydrogen atoms were assigned from the solution. Full-matrix least squares / difference Fourier cycles were performed which located any remaining non-hydrogen atoms. All non-hydrogen atoms were refined with anisotropic displacement parameters. All hydrogen atoms were placed in ideal positions and refined as riding atoms with relative isotropic displacement parameters. The final full matrix least squares refinement converged to $R1 = 0.0310$ (F^2, $I > 2\sigma(I)$) and $wR2 = 0.0787$ (F^2, all data). Structure solution and refinement were performed within the Olex2 program.[5]

The asymmetric unit contains one cation-anion pairing in a general position. Part of the 18-crown-6 ether is modeled as disordered over two positions (0.60:0.40). The alternating cations and anions are linked one-dimensionally via K…O intercations.

[K(18-crown-6)][ZnL^{OMe}]. A crystal (0.349 x 0.147 x 0.043 mm^3) was placed onto a nylon loop and mounted on a Rigaku XtaLAB Synergy-S Dualflex diffractometer equipped with a HyPix-6000HE HPC area detector for data collection at 100.00(10) K. A preliminary set of cell constants and an orientation matrix were calculated from a small sampling of reflections.[2] A short pre-experiment was run, from which an optimal data collection strategy was determined. The full data collection was carried out using a PhotonJet (Cu) X-ray source with frame times of 0.15 and 0.59 seconds and a detector distance of 34.0 mm. Series of frames were collected in 0.50° steps in ω at different 2θ, κ, and ϕ settings. After the intensity data were corrected for absorption, the final cell constants were calculated from the xyz centroids of 51880 strong reflections from the actual data collection after integration.[2] See Table S5.7 for additional crystal and refinement information.

The structure was solved using SHELXT[3] and refined using SHELXL.[4] The space group $P2_1/c$ was determined based on systematic absences. Most or all non-hydrogen atoms were assigned from the solution. Full-matrix least squares / difference Fourier cycles were performed which located any remaining non-hydrogen atoms. All non-hydrogen atoms were refined with anisotropic displacement parameters. All hydrogen atoms were placed in ideal positions and refined as riding atoms with relative isotropic displacement parameters. The final full matrix least squares refinement converged to $R1 = 0.0329$ (F^2, $I > 2\sigma(I)$) and $wR2 = 0.0861$ (F^2, all data). Structure solution and refinement were performed within the Olex2 program.[5]

The asymmetric unit contains one cation-anion pairing in a general position. One segment of the crown ether, O13-O15, is modeled as disordered over two positions (0.57:0.43). The alternating cations and anions are linked one-dimensionally via K…O intercations.

[K(18-crown-6)][Co(NCMe)L^{OMe}]• 1.50 MeCN. A crystal (0.152 x 0.12 x 0.044 mm^3) was placed onto a nylon loop and mounted on a Rigaku XtaLAB Synergy-S Dualflex diffractometer equipped with a HyPix-6000HE HPC area detector for data collection at 100.00(10) K. A preliminary set of cell constants and an orientation matrix were calculated from a small sampling of reflections.[2] A short pre-experiment was run, from which an optimal data collection strategy

was determined. The full data collection was carried out using a PhotonJet (Cu) X-ray source with frame times of 1.94 and 7.75 seconds and a detector distance of 34.0 mm. Series of frames were collected in 0.50° steps in ω at different 2θ, κ, and ϕ settings. After the intensity data were corrected for absorption, the final cell constants were calculated from the xyz centroids of 20177 strong reflections from the actual data collection after integration.[2] See Table S5.8 for additional crystal and refinement information.

The structure was solved using SHELXT[3] and refined using SHELXL.[4] The space group *P*-1 was determined based on intensity statistics. Most or all non-hydrogen atoms were assigned from the solution. Full-matrix least squares / difference Fourier cycles were performed which located any remaining non-hydrogen atoms. All non-hydrogen atoms were refined with anisotropic displacement parameters. All hydrogen atoms were placed in ideal positions and refined as riding atoms with relative isotropic displacement parameters. The final full matrix least squares refinement converged to $R1 = 0.0600$ (F^2, $I > 2\sigma(I)$) and $wR2 = 0.1641$ (F^2, all data). Structure solution and refinement were performed within the Olex2 program.[5]

The asymmetric unit contains one monoanionic cobalt complex in contact with one acetonitrile-ligated potassium cation, all in general positions. The structure is one-dimensional polymeric along [100] via the cation-anion contacts. Acetonitrile solvent molecule N7-C38-C39 is modeled as disordered over a crystallographic inversion center (0.50:0.50).

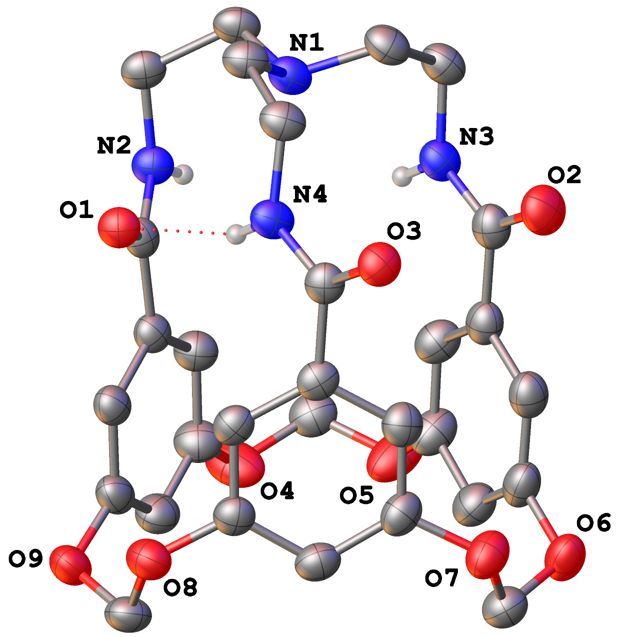

Figure S30. Molecular structure of $\mathbf{H_3L^{OCH2O}}$. Hydrogen atoms (except for N-H) are omitted for clarity. Anisotropic displacement ellipsoids are drawn at the 50% probability level.

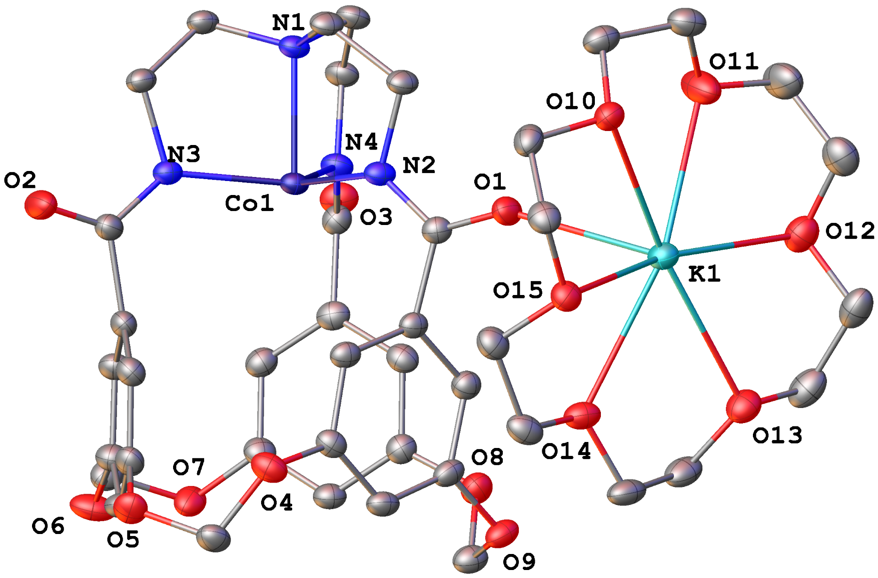

Figure S31. Molecular structure of **[K(18-crown-6)][CoL^{OCH2O}] • MeCN**. Hydrogen atoms and co-crystallized solvent molecules are omitted for clarity. Anisotropic displacement ellipsoids are drawn at the 50% probability level.

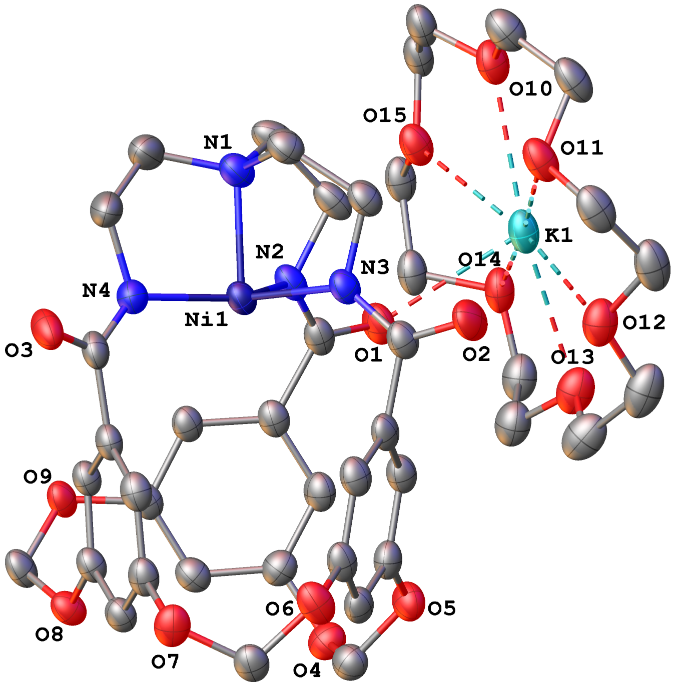

Figure S32. Molecular structure of **[K(18-crown-6)][NiL^{OCH2O}] • 0.15 MeCN**. Hydrogen atoms, co-crystallized solvent molecules, chemically equivalent molecules in asymmetric unit, and disordered components are omitted for clarity. Anisotropic displacement ellipsoids are drawn at the 50% probability level.

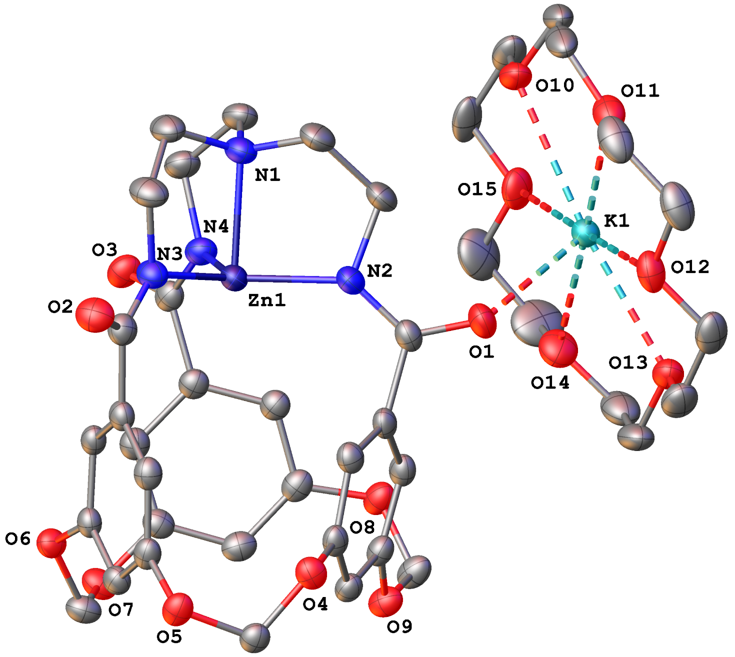

Figure S33. Molecular structure of **[K(18-crown-6)][ZnL$^{\mathbf{OCH2O}}$] • MeCN**. Hydrogen atoms and co-crystallized solvent molecules are omitted for clarity. Anisotropic displacement ellipsoids are drawn at the 50% probability level. Only the major compontent of disordered parts are shown.

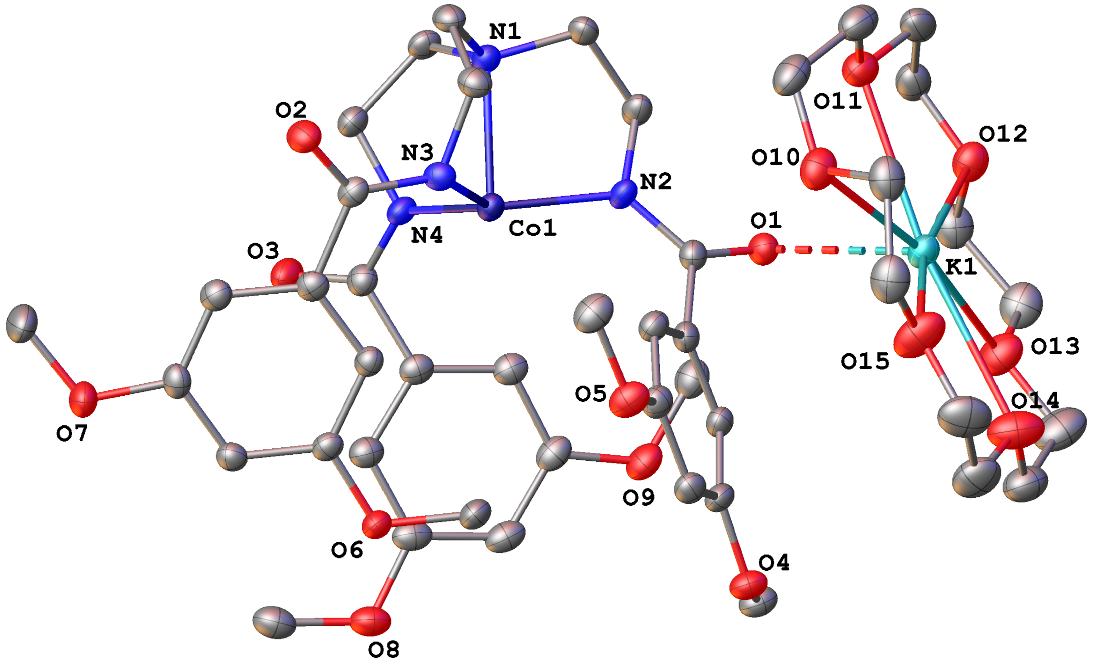

Figure S34. Molecular structure of **[K(18-crown-6)][CoL$^{\mathbf{OMe}}$]**. Hydrogen atoms are omitted for clarity. Anisotropic displacement ellipsoids are drawn at the 50% probability level.

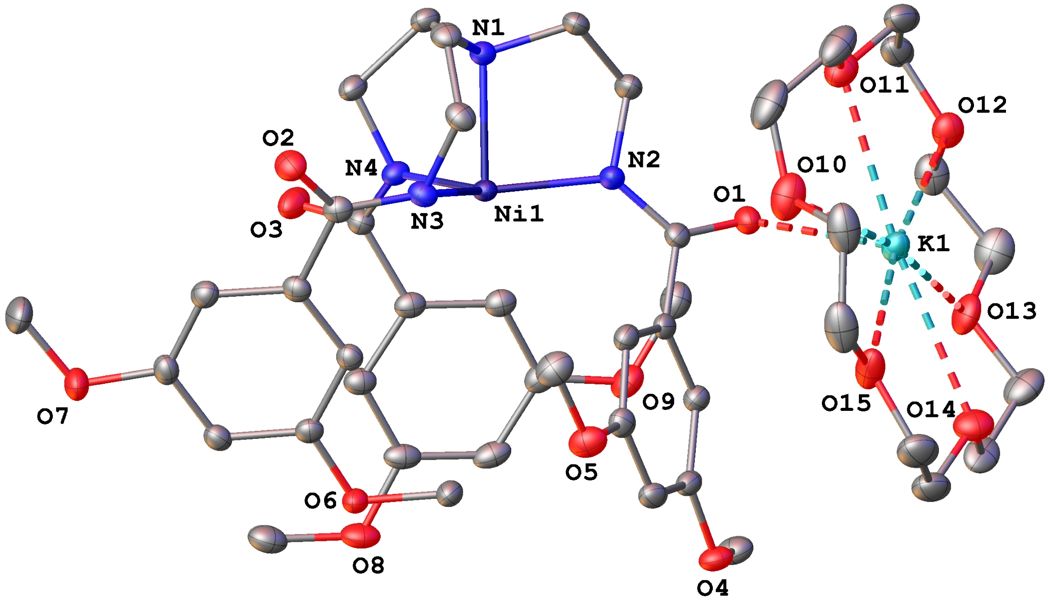

Figure S35. Molecular structure of **[K(18-crown-6)][NiL^{OMe}]**. Hydrogen atoms are omitted for clarity. Anisotropic displacement ellipsoids are drawn at the 50% probability level. Only the major compontent of disordered parts are shown.

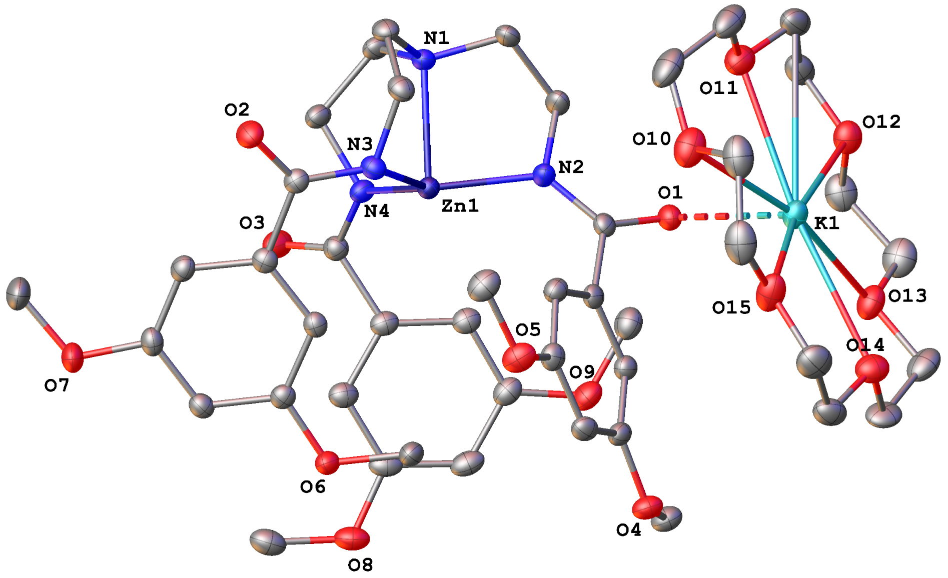

Figure S36. Molecular structure of **[K(18-crown-6)][ZnL^{OMe}]**. Hydrogen atoms are omitted for clarity. Anisotropic displacement ellipsoids are drawn at the 50% probability level. Only the major compontent of disordered parts are shown.

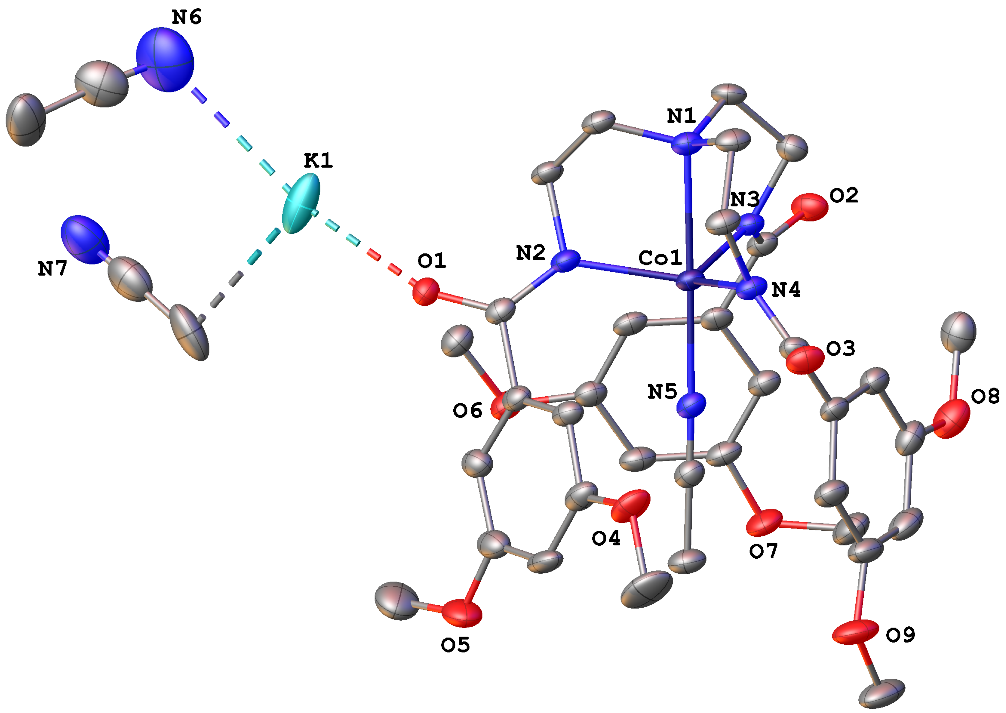

Figure S37. Molecular structure of **[K(18-crown-6)][Co(NCMe)L^{OMe}]• 1.50 MeCN**. Hydrogen atoms are omitted for clarity. Anisotropic displacement ellipsoids are drawn at the 50% probability level.

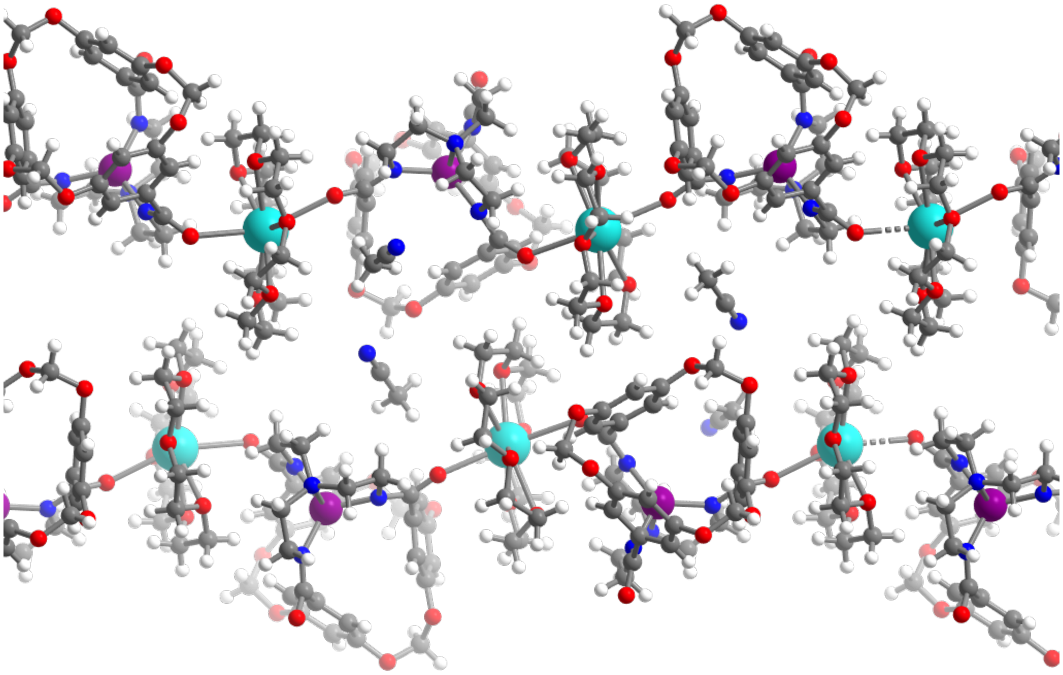

Figure S38. View of the infinite one-dimensional chains formed through cation-anion contacts in **[K(18-crown-6)][CoL^{OCH2O}] • MeCN.**

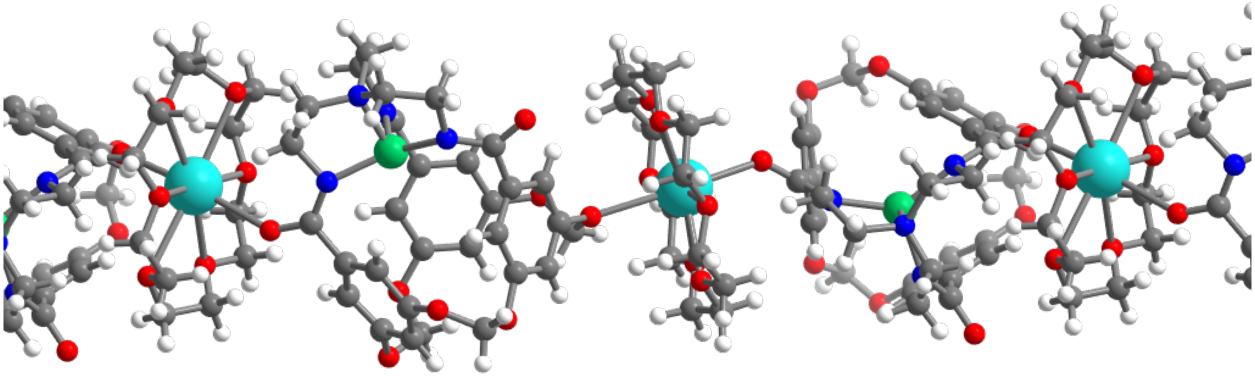

Figure S39. View of the infinite one-dimensional chains formed through cation-anion contacts in **[K(18-crown-6)][NiL^{OCH2O}] • 0.15 MeCN.** Only the major compontent of disordered parts are shown.

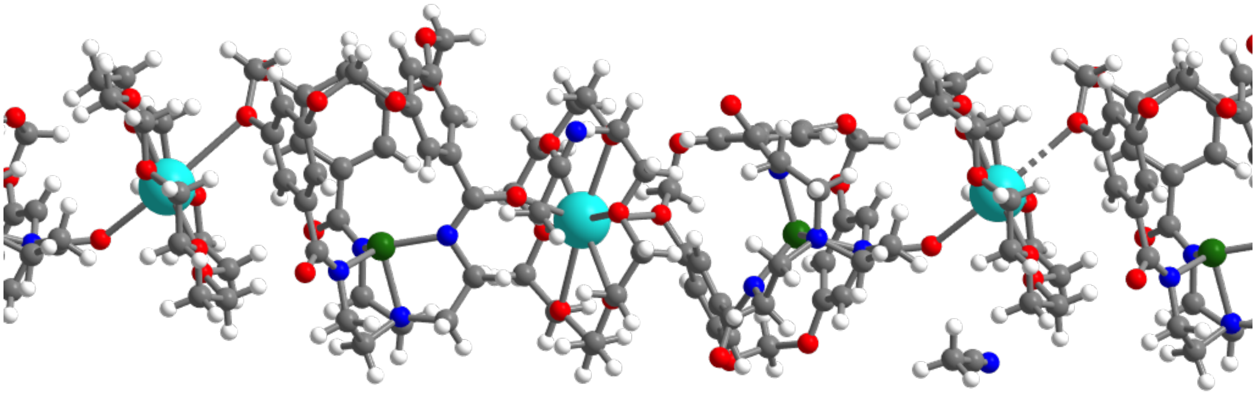

Figure S40. View of the infinite one-dimensional chains formed through cation-anion contacts in **[K(18-crown-6)][ZnL^{OCH2O}] • MeCN.** Only the major compontent of disordered parts are shown.

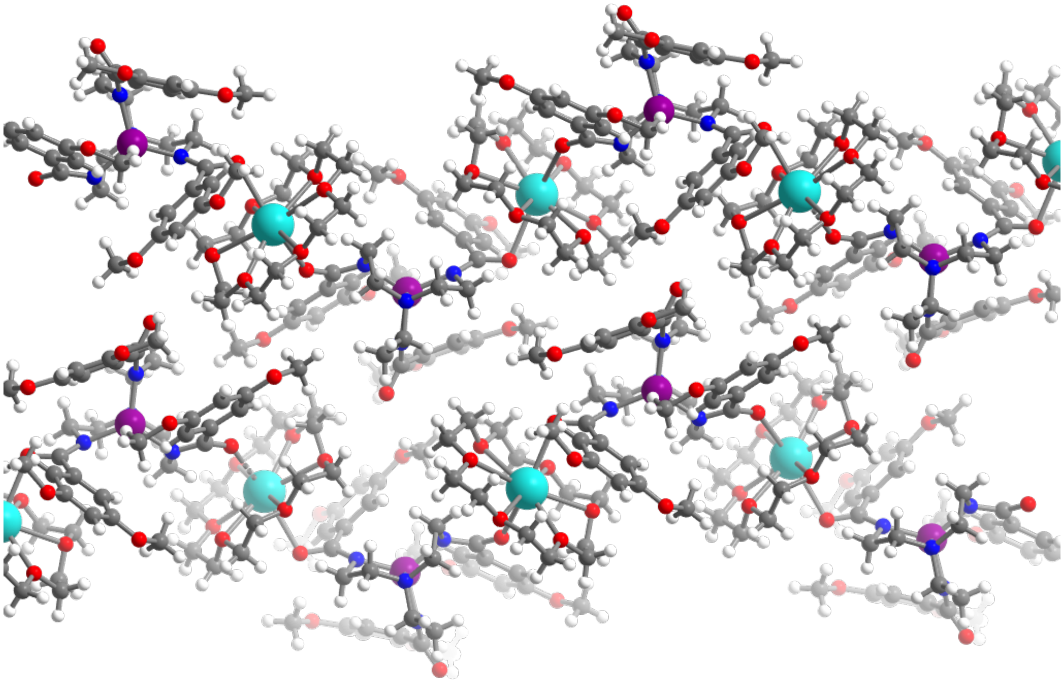

Figure S41. View of the infinite one-dimensional chains formed through cation-anion contacts in **[K(18-crown-6)][CoL^{OMe}].** The nickel and zinc congeners pack in isomorphous configurations.

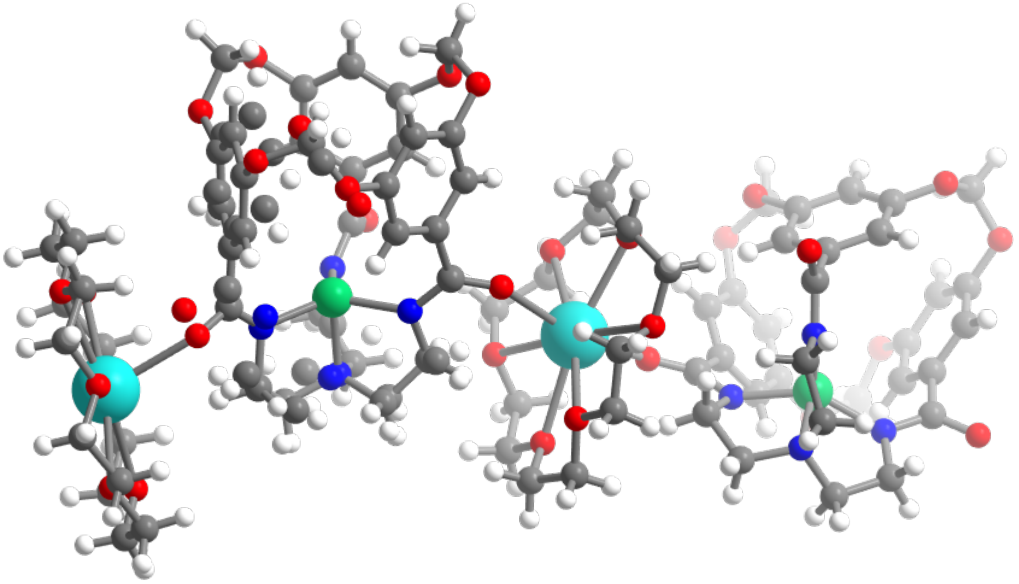

Figure S42. View of asymmetric unit in **[K(18-crown-6)][NiL^{OCH2O}] • 0.15 MeCN.** Note that one crystallographically independent nickel complex contains some minor disorder in the primary coordination sphere (both disordered parts are shown here). Co-crytallized acetonitrile at partial occupancy is omitted for clarity.

Table S1. Crystal data and structure refinement for $\mathbf{H_3L^{OCH2O}}$.

Identification code	brbcdh03	
Empirical formula	C30 H30 N4 O9	
Formula weight	590.58	
Temperature	100.01(10) K	
Wavelength	1.54184 Å	
Crystal system	monoclinic	
Space group $P2_1/c$		
Unit cell dimensions	a = 15.0315(4) Å	α = 90°
	b = 12.1201(3) Å	β = 106.995(2)°
	c = 15.7248(3) Å	γ = 90°
Volume	2739.69(12) $Å^3$	
Z	4	
Density (calculated)	1.432 Mg/m^3	
Absorption coefficient	0.897 mm^{-1}	
$F(000)$	1240	
Crystal color, morphology	colourless, plate	
Crystal size	0.12 x 0.107 x 0.018 mm^3	
Theta range for data collection	3.074 to 80.255°	
Index ranges	$-19 \leq h \leq 19$, $-15 \leq k \leq 14$, $-19 \leq l \leq 11$	
Reflections collected	28201	
Independent reflections	5819 [R(int) = 0.0344]	
Observed reflections	4711	
Completeness to theta = 70.076°	100.0%	
Absorption correction	Multi-scan	
Max. and min. transmission	1.00000 and 0.75174	
Refinement method	Full-matrix least-squares on F^2	
Data / restraints / parameters	5819 / 0 / 400	
Goodness-of-fit on F^2	1.067	
Final R indices [I>2sigma(I)]	$R1$ = 0.0499, $wR2$ = 0.1406	
R indices (all data)	$R1$ = 0.0614, $wR2$ = 0.1490	
Largest diff. peak and hole	0.429 and -0.248 e.$Å^{-3}$	

Table S2. Crystal data and structure refinement for **[K(18-crown-6)][CoL^{OCH2O}]•MeCN**.

Identification code	brbcdh06	
Empirical formula	C44 H54 Co K N5 O15	
Formula weight	990.95	
Temperature	100.00(10) K	
Wavelength	1.54184 Å	
Crystal system	monoclinic	
Space group	$P2_1/c$	
Unit cell dimensions	a = 16.99130(10) Å	α = 90°
	b = 11.24430(10) Å	β = 93.5670(10)°
	c = 23.72370(10) Å	γ = 90°
Volume	4523.76(5) Å^3	
Z	4	
Density (calculated)	1.455 Mg/m^3	
Absorption coefficient	4.440 mm^{-1}	
F(000)	2076	
Crystal color, morphology	blue, block	
Crystal size	0.162 x 0.145 x 0.102 mm^3	
Theta range for data collection	3.734 to 80.245°	
Index ranges	$-21 \leq h \leq 21$, $-14 \leq k \leq 12$, $-30 \leq l \leq 29$	
Reflections collected	59622	
Independent reflections	9761 [R(int) = 0.0339]	
Observed reflections	9114	
Completeness to theta = 74.504°	99.9%	
Absorption correction	Multi-scan	
Max. and min. transmission	1.00000 and 0.80334	
Refinement method	Full-matrix least-squares on F^2	
Data / restraints / parameters	9761 / 0 / 596	
Goodness-of-fit on F^2	1.061	
Final R indices [I>2sigma(I)]	R1 = 0.0405, wR2 = 0.0999	
R indices (all data)	R1 = 0.0433, wR2 = 0.1014	
Largest diff. peak and hole	0.431 and -0.462 e.Å^{-3}	

Table S3. Crystal data and structure refinement for **[K(18-crown-6)][NiL^{OCH2O}]• 0.15 MeCN**.

Identification code	brbcdh16	
Empirical formula	C42.31 H51.46 K N4.15 Ni O15	
Formula weight	955.97	
Temperature	100.00(10) K	
Wavelength	1.54184 Å	
Crystal system	triclinic	
Space group	*P*-1	
Unit cell dimensions	*a* = 12.35190(10) Å	α = 75.9250(10)°
	b = 15.6577(2) Å	β = 78.4200(10)°
	c = 23.4000(2) Å	γ = 89.7380(10)°
Volume	4295.73(8) Å^3	
Z	4	
Density (calculated)	1.478 Mg/m^3	
Absorption coefficient	2.167 mm^{-1}	
F(000)	2005	
Crystal color, morphology	orange, block	
Crystal size	0.223 x 0.151 x 0.126 mm^3	
Theta range for data collection	3.657 to 80.628°	
Index ranges	$-15 \le h \le 15$, $-19 \le k \le 20$, $-29 \le l \le 29$	
Reflections collected	109541	
Independent reflections	18488 [*R*(int) = 0.0421]	
Observed reflections	16783	
Completeness to theta = 74.504°	99.7%	
Absorption correction	Multi-scan	
Max. and min. transmission	1.00000 and 0.79588	
Refinement method	Full-matrix least-squares on F^2	
Data / restraints / parameters	18488 / 257 / 1365	
Goodness-of-fit on F^2	1.039	
Final *R* indices [*I*>2sigma(*I*)]	*R*1 = 0.0442, *wR*2 = 0.1059	
R indices (all data)	*R*1 = 0.0479, *wR*2 = 0.1081	
Largest diff. peak and hole	0.730 and -1.087 e.Å^{-3}	

Table S4. Crystal data and structure refinement for **[K(18-crown-6)][ZnL^{OCH2O}]• MeCN**.

Identification code	brbcdh20	
Empirical formula	C44 H54 K N5 O15 Zn	
Formula weight	997.39	
Temperature	100.00(10) K	
Wavelength	1.54184 Å	
Crystal system	orthorhombic	
Space group	*Pbca*	
Unit cell dimensions	*a* = 21.28610(10) Å	α = 90°
	b = 18.00390(10) Å	β = 90°
	c = 23.98730(10) Å	γ = 90°
Volume	9192.72(8) $Å^3$	
Z	8	
Density (calculated)	1.441 Mg/m^3	
Absorption coefficient	2.185 mm^{-1}	
F(000)	4176	
Crystal color, morphology	colourless, block	
Crystal size	0.34 x 0.128 x 0.046 mm^3	
Theta range for data collection	3.706 to 80.337°	
Index ranges	$-27 \leq h \leq 25$, $-23 \leq k \leq 17$, $-30 \leq l \leq 30$	
Reflections collected	82764	
Independent reflections	9907 [*R*(int) = 0.0460]	
Observed reflections	9188	
Completeness to theta = 74.504°	99.9%	
Absorption correction	Multi-scan	
Max. and min. transmission	1.00000 and 0.57642	
Refinement method	Full-matrix least-squares on F^2	
Data / restraints / parameters	9907 / 310 / 788	
Goodness-of-fit on F^2	1.072	
Final *R* indices [*I*>2sigma(*I*)]	*R*1 = 0.0348, *wR*2 = 0.0905	
R indices (all data)	*R*1 = 0.0374, *wR*2 = 0.0921	
Largest diff. peak and hole	0.314 and -0.463 e.$Å^{-3}$	

Table S5. Crystal data and structure refinement for **[K(18-crown-6)][CoL^{OMe}]**.

Identification code	brbcdh13	
Empirical formula	C45 H63 Co K N4 O15	
Formula weight	998.02	
Temperature	100.01(10) K	
Wavelength	1.54184 Å	
Crystal system	monoclinic	
Space group	$P2_1/c$	
Unit cell dimensions	a = 10.5557(2) Å	α = 90°
	b = 20.3984(4) Å	β = 91.6290(10)°
	c = 21.9563(4) Å	γ = 90°
Volume	4725.71(15) Å^3	
Z	4	
Density (calculated)	1.403 Mg/m^3	
Absorption coefficient	4.244 mm^{-1}	
F(000)	2108	
Crystal color, morphology	light blue-green, needle	
Crystal size	0.484 x 0.049 x 0.029 mm^3	
Theta range for data collection	4.028 to 80.593°	
Index ranges	$-13 \leq h \leq 13$, $-25 \leq k \leq 19$, $-26 \leq l \leq 28$	
Reflections collected	44654	
Independent reflections	10096 [R(int) = 0.0577]	
Observed reflections	8836	
Completeness to theta = 74.504°	99.7%	
Absorption correction	Multi-scan	
Max. and min. transmission	1.00000 and 0.66828	
Refinement method	Full-matrix least-squares on F^2	
Data / restraints / parameters	10096 / 0 / 601	
Goodness-of-fit on F^2	1.070	
Final R indices [I>2sigma(I)]	$R1$ = 0.0516, $wR2$ = 0.1468	
R indices (all data)	$R1$ = 0.0588, $wR2$ = 0.1518	
Largest diff. peak and hole	0.831 and -0.966 e.Å^{-3}	

Table S6. Crystal data and structure refinement for **[K(18-crown-6)][NiL^{OMe}]**.

Identification code	brbcdh18	
Empirical formula	C45 H63 K N4 Ni O15	
Formula weight	997.80	
Temperature	99.99(10) K	
Wavelength	1.54184 Å	
Crystal system	monoclinic	
Space group	$P2_1/c$	
Unit cell dimensions	a = 10.57836(5) Å	α = 90°
	b = 20.59549(10) Å	β = 91.7664(4)°
	c = 21.63162(10) Å	γ = 90°
Volume	4710.57(4) Å^3	
Z	4	
Density (calculated)	1.407 Mg/m^3	
Absorption coefficient	1.995 mm^{-1}	
F(000)	2112	
Crystal color, morphology	orange-pink, block	
Crystal size	0.357 x 0.188 x 0.185 mm^3	
Theta range for data collection	4.089 to 80.518°	
Index ranges	$-10 \le h \le 13$, $-26 \le k \le 25$, $-27 \le l \le 27$	
Reflections collected	81656	
Independent reflections	10176 [R(int) = 0.0360]	
Observed reflections	9466	
Completeness to theta = 74.504°	99.8%	
Absorption correction	Multi-scan	
Max. and min. transmission	1.00000 and 0.68422	
Refinement method	Full-matrix least-squares on F^2	
Data / restraints / parameters	10176 / 104 / 665	
Goodness-of-fit on F^2	1.055	
Final R indices [I>2sigma(I)]	$R1$ = 0.0310, $wR2$ = 0.0775	
R indices (all data)	$R1$ = 0.0332, $wR2$ = 0.0787	
Largest diff. peak and hole	0.580 and -0.347 e.Å^{-3}	

Table S7. Crystal data and structure refinement for **[K(18-crown-6)][ZnL^{OMe}]**.

Identification code	brbcdh14	
Empirical formula	C45 H63 K N4 O15 Zn	
Formula weight	1004.46	
Temperature	100.00(10) K	
Wavelength	1.54184 Å	
Crystal system	monoclinic	
Space group	$P2_1/c$	
Unit cell dimensions	a = 10.59430(10) Å	α = 90°
	b = 20.38260(10) Å	β = 91.7450(10)°
	c = 21.90420(10) Å	γ = 90°
Volume	4727.79(5) Å^3	
Z	4	
Density (calculated)	1.411 Mg/m^3	
Absorption coefficient	2.118 mm^{-1}	
F(000)	2120	
Crystal color, morphology	colourless, plate	
Crystal size	0.349 x 0.147 x 0.043 mm^3	
Theta range for data collection	4.038 to 80.173°	
Index ranges	$-13 \leq h \leq 13$, $-25 \leq k \leq 26$, $-23 \leq l \leq 27$	
Reflections collected	82161	
Independent reflections	10235 [R(int) = 0.0423]	
Observed reflections	9465	
Completeness to theta = 74.504°	100.0%	
Absorption correction	Multi-scan	
Max. and min. transmission	1.00000 and 0.79163	
Refinement method	Full-matrix least-squares on F^2	
Data / restraints / parameters	10235 / 99 / 665	
Goodness-of-fit on F^2	1.075	
Final R indices [I>2sigma(I)]	$R1$ = 0.0329, $wR2$ = 0.0846	
R indices (all data)	$R1$ = 0.0355, $wR2$ = 0.0861	
Largest diff. peak and hole	0.702 and -0.493 e.Å^{-3}	

Table S8. Crystal data and structure refinement for **[K(18-crown-6)][Co(NCMe)L^{OMe}] • 1.5 MeCN**

Identification code	brbbrb17	
Empirical formula	C38 H46.50 Co K N6.50 O9	
Formula weight	836.34	
Temperature	100.00(10) K	
Wavelength	1.54184 Å	
Crystal system	triclinic	
Space group	*P*-1	
Unit cell dimensions	*a* = 12.0132(4) Å	α = 89.936(3)°
	b = 13.1896(4) Å	β = 87.088(3)°
	c = 13.2685(4) Å	γ = 70.141(3)°
Volume	1974.52(12) $Å^3$	
Z	2	
Density (calculated)	1.407 Mg/m^3	
Absorption coefficient	4.873 mm^{-1}	
F(000)	876	
Crystal color, morphology	violet, plate	
Crystal size	0.152 x 0.12 x 0.044 mm^3	
Theta range for data collection	3.563 to 80.118°	
Index ranges	$-15 \le h \le 12$, $-16 \le k \le 16$, $-16 \le l \le 16$	
Reflections collected	45147	
Independent reflections	8411 [*R*(int) = 0.0566]	
Observed reflections	7597	
Completeness to theta = 74.504°	99.5%	
Absorption correction	Multi-scan	
Max. and min. transmission	1.00000 and 0.80836	
Refinement method	Full-matrix least-squares on F^2	
Data / restraints / parameters	8411 / 0 / 523	
Goodness-of-fit on F^2	1.049	
Final *R* indices [*I*>2sigma(*I*)]	*R*1 = 0.0600, *wR*2 = 0.1602	
R indices (all data)	*R*1 = 0.0651, *wR*2 = 0.1641	
Largest diff. peak and hole	1.702 and -1.217 e.$Å^{-3}$	

S6. Details of Density Functional Theory Calculations

S6.1. General Considerations. Calculations were carried out using the ORCA program package.[6] Geometry optimizations and single-point calculations were performed using the OLYP Generalized Gradient Approximation (GGA) functional, which utilizes OPTX GGA exchange and LYP GGA correlation.[7] In all calculations, the functional was appended with the D3 dispersion correction.[8] The Alhrichs all-electron triple-zeta basis set def2-TZVP(-f)[9] was employed for cobalt, while light atoms were assigned the def2-SVP basis set.[10] All atoms were assigned the def2/J auxiliary basis set.

Geometry optimizations utilized the TIGHTOPT and TIGHTSCF criteria. For all Co complexes investigated here, the S=3/2 (high-spin) solution was found to be lower in energy than the corresponding S=1/2 (low-spin) solution. The fully relaxed coordinates were used to perform a numerical frequency calculation using the NUMFREQ command. All frequency calculations on fully relaxed structures were confirmed to be devoid of imaginary vibrational frequencies.

For relaxed surface scans along the one-dimensional [Co---NCR] (R = Me, H) coordinate, NORMALOPT and TIGHTSCF criteria were used. The Co-$N_{nitrile}$ distance was varied in steps of 0.3 Å and was constrained to fixed values while all other coordinates were allowed to relax with the following exceptions:

- For both R = Me and R = H, the N_{axial}-Co-$N_{nitrile}$ angle was held fixed to the value of that from the fully relaxed geometry; this was done to ensure that the nitrile N atom moved along a one-dimensional coordinate.

- For R = H, the Co-$N_{nitrile}$-$C_{nitrile}$ angle was held fixed to the value of that from the fully relaxed geometry. Prior surface scans without this constraint resulted in rotation of the HCN moiety at longer Co-$N_{nitrile}$ distances, which enabled significant H-bonding interactions between *H*CN and an acetal O atom along the cavity crest. As we were interested in a general understanding of the energy barrier required to force a fragment of given size through the aperture, perturbations resulting from H-bonding interactions unique to HCN were undesirable.

A single-point energy calculation was run at the conclusion of each constrained optimization, and the final single-point energy at each step was recorded and used to determine ΔE_{rel} across different Co-$N_{nitrile}$ distances.

S6.2. Constrained optimization and subsequent frequency calculation for [Co(NCMe)L^{OMe}]$^-$. Given the significantly greater C-N_{amine} bond length in the optimized structure of **[Co(NCMe)L^{OMe}]$^-$** compared to that in **[Co(NCMe)L^{OCH2O}]$^-$**, we performed a constrained optimization and frequency calculation meant to interrogate whether this bond length played a significant part in the large differences in calculated thermodynamic parameters of acetonitrile binding. A geometry optimization of **[Co(NCMe)L^{OMe}]$^-$** with a constrained Co-N_{amine} distance of 2.25 Å was performed, as this is the Co-N_{amine} distance for the fully relaxed geometry of **[Co(NCMe)L^{OCH2O}]$^-$**. Selected metrical parameters for this constrained optimization can be found in Table S6.5. A full numerical frequency calculation on this geometry allowed for the thermodynamics of acetonitrile binding to be calculated as $\Delta H_{273.15K}$ = -13.7 kcal/mol and $\Delta G_{273.15K}$ = 0.0 kcal/mol. Compared to the enthalpy and Gibbs free energy

changes for acetonitrile binding calculated without constrained geometries (-14.7 kcal/mol and -1.0 kcal/mol, respectively), it is evident that differences in Co-N_{amine} play only a very minor role in these values. Accordingly, there appears to be a rather shallow potential energy surface over the Co-N_{amine} coordinate in complexes $[Co(NCR)L^{OMe}]^{-}$.

S6.3. Comparison of Metrical Parameters between Experimental and Calculated Structures.

Table S9. Comparison of metrical parameters for $\mathbf{[CoL^{OCH2O}]^{-}}$

**Parameter**	**Experimental (XRD)**	**Calculated**
Co–N_{amine}	2.1015(16) Å	2.145 Å
Co–N_{amide} (mean)	1.992(3) Å	1.980 Å
τ_4	0.86	0.86

Table S10. Comparison of metrical parameters for $\mathbf{[Co(NCMe)L^{OCH2O}]^{-}}$

**Parameter**	**Experimental (XRD)**	**Calculated**
Co–N_{amine}	n/a	2.250 Å
Co–N_{amide} (mean)	n/a	2.055 Å
Co–$N_{nitrile}$	n/a	1.965 Å
τ_5	n/a	0.99

Table S11. Comparison of metrical parameters for $\mathbf{[Co(NCH)L^{OCH2O}]^{-}}$

**Parameter**	**Experimental (XRD)**	**Calculated**
Co–N_{amine}	n/a	2.232 Å
Co–N_{amide} (mean)	n/a	2.047 Å
Co–$N_{nitrile}$	n/a	1.940 Å
τ_5	n/a	1.02

Table S12. Comparison of metrical parameters for $\mathbf{[CoL^{OMe}]^{-}}$

**Parameter**	**Experimental (XRD)**	**Calculated**
Co–N_{amine}	2.1605(19) Å	2.222 Å
Co–N_{amide} (mean)	1.972(3) Å	1.957 Å
τ_4	0.85	0.83

Table S13. Comparison of metrical parameters for **[Co(NCMe)L^{OMe}]$^{-}$**

**Parameter**	**Experimental (XRD)**	**Calculated** **(fully relaxed)**	**Calculated** **(Co–N$_{amine}$ constrained)**
Co–N$_{amine}$	2.299(2) Å	2.435 Å	2.250 Å (fixed)
Co–N$_{amide}$ (mean)	2.037(3) Å	2.009 Å	2.018 Å
Co–N$_{nitrile}$	2.113(2) Å	1.995 Å	2.019 Å
τ_5	1.02	1.00	0.97

Table S14. Comparison of metrical parameters for **[Co(NCH)L^{OMe}]$^{-}$**

**Parameter**	**Experimental (XRD)**	**Calculated**
Co–N$_{amine}$	n/a	2.411 Å
Co–N$_{amide}$ (mean)	n/a	2.005 Å
Co–N$_{nitrile}$	n/a	1.971 Å
τ_5	n/a	1.00

S6.4. Input Files for Geometry Optimizations

[CoL^{OCH2O}]$^{-}$

```
! UKS OLYP Opt RI def2-SVP def2/J D3 TightSCF TightOpt SlowConv
%SCF
 MaxIter 5000
end
%geom
 MaxIter 2000
end
%basis
newgto Co "def2-TZVP(-f)" end
end

* xyz -1 4
 Co  13.28778483227411    8.28780174188942    8.42621460219232
 H   16.98566515165259    2.65051759694158    4.89622217916398
 O   12.37122369074996    8.75349368478547    4.34220005283772
 O   17.25006233004017    7.66562517210762    9.85046987621792
 O   10.17332731461672    7.57727719998737   11.17609780401948
 O   15.87253256632516    4.34890052587317    4.91280714160234
 O   17.11300616478518    3.48080073369211    6.70941296992014
 O   14.60036080258516    2.70401357429350   10.67508709180025
 O   12.26924235565580    2.70537532337299   10.95759867698990
 O   10.00501942816287    3.86094624196339    6.86052139305580
 O   11.07050808829454    3.64558111236513    4.78155772969638
 C   13.52440992267530    2.96291447345549   11.54277675647289
 H   13.57781835581556    3.98005095359900   11.97079598790359
 H   13.62022624130358    2.22097291488543   12.35434119706563
 C   10.63664886312780    2.98952257440669    5.94752474568193
 H   11.47602664519295    2.45648421086764    6.43686331357180
 H    9.87629050651245    2.26670987010041    5.59865215826571
```

N	13.33511724092383	10.42618645658602	8.81819794767767
N	13.24982099088317	8.85760286781619	6.47989838601753
N	15.05995015525588	8.25986950819238	9.41905744038875
N	11.57447033220664	8.36400433703156	9.51100895526210
C	13.79388636786804	11.00227890540296	7.54580231661178
H	14.88402985900314	10.85406480990719	7.48875729968839
H	13.61295286766383	12.10098150367225	7.50589549733151
C	13.14229766133724	10.30833496474937	6.35079374301781
H	12.08402894410895	10.62617699998046	6.23198835973635
H	13.63113864505628	10.66491204864588	5.42171108956848
C	12.85946213455246	8.21136489434203	5.35018446188107
C	13.07589107649900	6.70746907230351	5.28191084232733
C	14.37164670617016	6.16545750575620	5.26340722572048
H	15.24294687357395	6.80780068853334	5.41320376057498
C	14.56945660046204	4.79222021009949	5.03211729598147
C	13.46563616436612	3.93940995519881	4.85909994929268
H	13.58817024218152	2.87291289123566	4.65102692985561
C	12.17016836629408	4.48410643872023	4.92350439402761
C	11.97141843797666	5.85772164611823	5.08521285631375
H	10.95493482159556	6.25802150556613	5.06856373600559
C	14.28677625855895	10.54820848460565	9.93136687718473
H	13.74877506591717	10.27777338243563	10.85357031120602
H	14.63030508784596	11.60004759783862	10.05950233107762
C	15.48468888511833	9.61450097402967	9.77382749130861
H	16.20155029328501	10.01075675176364	9.02282942809382
H	16.06076769235872	9.61931762611131	10.72029626005954
C	16.08619796159694	7.37558480510466	9.51582454063474
C	15.84797327203455	5.90547106546048	9.19045855421620
C	15.15018802912812	5.04769992055055	10.06417286544633
H	14.64274680715381	5.48210431129717	10.92739083731800
C	15.14498297498003	3.65761618246484	9.83285087098075
C	15.79585484202846	3.12784582408415	8.70523927697350
H	15.78160472059363	2.04709301913737	8.53651874872022
C	16.46383829742535	3.99250704944285	7.82642886058153
C	16.52276840112223	5.36604382554325	8.08037829929914
H	17.09619339105605	6.00900536049779	7.40830843249934
C	11.94942870607389	10.76657207178053	9.17087671049306
H	11.37656037570123	10.84021662978350	8.23349837827353
H	11.88992757001011	11.76381037749344	9.66358370100725
C	11.31067016805186	9.69456313493547	10.05034321529728
H	11.66344932824535	9.77730536614227	11.10035579341779
H	10.22161721954151	9.89112366055701	10.12018667716244
C	10.89889346819115	7.39633402933419	10.18093755557710
C	11.04759448616442	5.97037110175184	9.67844472590019
C	10.56082157288604	5.56595725898949	8.42557685459320
H	10.13669209356009	6.29439750623659	7.73129796422459
C	10.59883152348837	4.20804209830973	8.05710079907696
C	11.17670539930086	3.25729962544654	8.91597092290302
H	11.23306407410726	2.19555448361090	8.66201562286231
C	11.69638386068868	3.67884952672893	10.15316751547695
C	11.58030175305192	5.01170769712769	10.55951227157286
H	11.90055228356766	5.33050308768584	11.55423594358253
C	16.31526585222861	3.19197009800947	5.58868400457502
H	15.45678953718916	2.54843595126459	5.86865719466503

*

$[CoL^{OMe}]^{-}$

```
! UKS OLYP Opt RI def2-SVP def2/J D3 TightSCF TightOpt SlowConv
%SCF
 MaxIter 5000
end
%geom
 MaxIter 2000
end
%basis
newgto Co "def2-TZVP(-f)" end
end

* xyz -1 4
Co      4.20775213      5.52113717      8.64449483
H      -0.34848916      4.62388025      7.37616751
O       4.08596024      9.67325504      8.09369221
O       5.09072850      1.82986279      7.50175774
O       3.47521602      3.61720515     12.21124207
O      -1.04208536      8.92480065      7.52880402
O       1.01954821      5.37231035      5.27207557
O      -0.39611034      3.61232364      9.19904711
O       2.11721948      0.33932417     11.39937402
O      -1.36988867      5.47712645     11.95493353
O       1.44836946      9.08673404     11.01540249
H       0.35524294      5.51020886      8.76640394
H      -1.41410097      5.28158375      8.67121111
C       3.39399329      0.03317677     11.92129447
H       3.97951049     -0.61482876     11.23909154
H       3.22266430     -0.51261283     12.86425046
H       3.96069004      0.95969473     12.12356784
C       3.45918721      4.54097696     11.37769147
C       2.27946339      5.49217276     11.33943601
C       1.01413349      4.99713018     11.68063033
H       0.93532205      3.93999102     11.91528699
C      -0.08835318      5.86272326     11.68644331
C       0.08544105      7.22973679     11.42801321
H      -0.77067401      7.90462634     11.47994329
C       1.36523707      7.73193991     11.15688726
C       2.46335827      6.85963170     11.06845102
H       3.45692198      7.22248667     10.81773021
C      -1.59807771      4.11781924     12.25390403
H      -1.34660976      3.47636139     11.39439416
H      -2.67331513      4.02759820     12.47987649
H      -1.01643211      3.78707533     13.13638606
C       2.72541453      9.68997802     11.10772512
H       3.33884791      9.52502685     10.20865459
H       3.26877625      9.32926938     12.00241761
H       2.54522232     10.77328745     11.20620727
N       6.49652867      5.37789011      8.67058207
N       4.65842041      7.44026620      8.31596093
N       4.44868311      4.04422058      7.30281585
N       4.43560909      4.83237159     10.48939187
C       6.95106506      6.72242520      8.32226609
H       6.98903667      6.78454343      7.22528436
```

H	7.97673309	6.90845936	8.70616933
C	5.97108678	7.77874286	8.81360770
H	5.97808034	7.80872086	9.92207466
H	6.27316588	8.78337571	8.46930501
C	6.81781712	4.36670819	7.66193587
H	6.85842102	3.38425244	8.14715662
H	7.81080123	4.56246616	7.20642559
C	5.70521227	4.28633001	6.61728339
H	5.63275283	5.23450504	6.05694974
H	5.94346290	3.46981860	5.91294915
C	6.79436598	4.97446053	10.04583474
H	6.85717499	5.88791923	10.65426862
H	7.77721689	4.46119674	10.10097571
C	5.67934924	4.10540567	10.61647504
H	5.64164907	3.13871637	10.08091006
H	5.89195481	3.86215633	11.67339293
C	3.84684993	8.45660856	7.95920632
C	2.52928265	8.00214118	7.38684779
C	1.38554246	8.74616436	7.69312344
H	1.50455133	9.62327223	8.32312174
C	0.13778142	8.31650764	7.21900083
C	0.04894807	7.19522719	6.38079587
H	-0.92256727	6.87166504	6.00518188
C	1.20305792	6.46700862	6.06134290
C	2.45160765	6.85961437	6.57434952
H	3.36137225	6.31776830	6.32602635
C	-0.99528665	10.01293200	8.42563298
H	-0.45786891	10.87887673	7.98997937
H	-0.50243114	9.74264694	9.37666394
H	-2.04053950	10.30149463	8.61989113
C	2.17290483	4.66625433	4.84711160
H	1.80856381	3.79683624	4.27809603
H	2.79073270	4.32269773	5.69466860
H	2.80099283	5.29807262	4.18835791
C	4.29299712	2.75900439	7.72571955
C	3.07625407	2.51278199	8.57661041
C	1.89873852	3.26648097	8.44098108
H	1.85498281	4.06909094	7.70851363
C	0.79009186	2.95035421	9.24471161
C	0.87674073	1.92367079	10.19513272
H	0.03723063	1.71177115	10.85752996
C	2.08071613	1.23516499	10.37269890
C	3.17076458	1.49756023	9.53277566
H	4.11119256	0.95620552	9.60391543
C	-0.44107603	4.81618323	8.45670864

*

[Co(NCMe)L^{OCH2O}]$^{-}$

```
! UKS OLYP Opt RI def2-SVP def2/J D3 TightSCF TightOpt SlowConv
%SCF
 MaxIter 5000
end
%geom
 MaxIter 2000
```

```
end
%basis
newgto Co "def2-TZVP(-f)" end
end

* xyz -1 4
Co     13.32280347     8.02448403     8.39219576
H      16.74194858     2.59335767     4.93210537
O      12.24456810     8.83387067     4.46117699
O      17.30218856     7.76074661     9.73368804
O      10.13155146     7.61145489     11.12025984
O      15.82084194     4.41448026     4.98445205
O      17.05090451     3.45923165     6.70890163
O      14.72439452     2.80021169     10.79312734
O      12.39900453     2.75502387     10.87221658
O       9.97786046     3.84018203     6.87741309
O      11.04912995     3.68191822     4.81733348
C      13.58125804     3.09784877     11.54714664
H      13.57079917     4.15113091     11.86841559
H      13.62988167     2.43899719     12.42892049
C      10.68160245     3.00555327     5.98763802
H      11.57097946     2.58050478     6.48759492
H       9.99532667     2.20485491     5.66040113
N      13.35452953     10.30864402     8.82965474
N      13.32301459     8.83875567     6.50069367
N      15.06507706     8.22701378     9.46142077
N      11.57423498     8.32109364     9.46661479
C      13.91543280     10.89961459     7.62520157
H      14.98998688     10.67323496     7.61237763
H      13.80015297     12.00510641     7.62610791
C      13.27331880     10.28469622     6.40076184
H      12.23379581     10.63997268     6.26516995
H      13.80004964     10.63204291     5.49207142
C      12.81489780     8.24117174     5.39147294
C      13.02392395     6.75541244     5.26304651
C      14.32170242     6.23477955     5.23245912
H      15.17975095     6.88679544     5.39871960
C      14.52413164     4.86175294     5.04692027
C      13.42711737     4.00261569     4.89430664
H      13.55401567     2.93307019     4.72039646
C      12.13164667     4.53657710     4.95078139
C      11.92045525     5.90450551     5.10500359
H      10.90450343     6.29927461     5.12942276
C      14.19967928     10.43332538     10.00469371
H      13.61830627     10.07913893     10.86619913
H      14.47277653     11.49355519     10.19630601
C      15.43489005     9.57302307     9.86559959
H      16.15629930     10.01281208     9.15059380
H      15.98145699     9.55467716     10.82628749
C      16.14070374     7.40017239     9.47923893
C      15.93508246     5.93218161     9.19782378
C      15.28463081     5.10126848     10.12225049
H      14.81659304     5.55499137     10.99551851
C      15.24190434     3.71675690     9.90199235
C      15.82265289     3.16735403     8.75197465
H      15.77801749     2.08891003     8.58802874
```

C 16.46215346 4.00861020 7.83655472
C 16.53857100 5.38256464 8.05850647
H 17.05695560 6.01826382 7.34031959
C 11.96728377 10.67536099 9.06414069
H 11.46109684 10.67873806 8.09078088
H 11.88907761 11.69881728 9.49052707
C 11.29481623 9.65679298 9.95612238
H 11.61515794 9.76783638 11.01052241
H 10.20585964 9.84627495 9.98175431
C 10.86857954 7.38489326 10.14532465
C 10.99646026 5.96088688 9.67248014
C 10.44877910 5.54678817 8.45497532
H 9.98452446 6.26456636 7.77829418
C 10.55384933 4.20408547 8.07052676
C 11.20868316 3.27784280 8.89401105
H 11.30838861 2.22657776 8.62085885
C 11.77154960 3.71278654 10.10303135
C 11.63441675 5.03921944 10.51248681
H 12.02234577 5.38990486 11.46838222
C 16.17656118 3.22146665 5.64345489
H 15.26921699 2.69552149 5.99411774
N 13.28472318 5.99051728 8.00953512
C 13.26265079 4.81140611 7.78770264
C 13.23505972 3.33751642 7.51041592
H 14.10817279 2.94142423 7.01323935
H 12.34483855 2.97330528 7.01923722
H 13.22843786 2.79552258 8.44454590
*

[Co(NCMe)L^{OMe}]$^{-}$

! UKS OLYP Opt RI def2-SVP def2/J D3 TightSCF TightOpt SlowConv
%SCF
 MaxIter 5000
end
%geom
 MaxIter 2000
end
%basis
newgto Co "def2-TZVP(-f)" end
end

* xyz -1 4
Co 1.53721887 0.17147683 -0.36922191
H -2.89755766 -2.59038360 -2.84930735
O 1.16005459 4.20841322 -1.30691533
O 2.54918325 -3.56516149 -1.21008853
O 1.31592070 -1.08133497 3.65570199
O -3.91430368 2.72199064 -1.95748522
O -1.22893277 -0.28346721 -4.38830116
O -3.26644534 -2.86385024 -0.80236008
O -0.78535312 -4.19885614 2.94618079
O -3.73435367 0.39164538 3.02535525
O -1.02386348 4.10260296 2.05858043
H -2.87268943 -1.09788177 -1.82799058

H	-4.42833021	-1.93230079	-2.17207931
C	0.42513846	-4.19854719	3.67721576
H	1.14053564	-4.94973851	3.28163068
H	0.15796892	-4.48196233	4.71038860
H	0.89820696	-3.19923992	3.67768530
C	1.12300132	-0.35487682	2.65197032
C	-0.07060545	0.56667331	2.64183114
C	-1.32486456	0.03971329	2.96296734
H	-1.40226318	-1.02674934	3.16433990
C	-2.45419027	0.86306886	2.88361510
C	-2.31863926	2.23120493	2.62207091
H	-3.20353758	2.86667651	2.55887990
C	-1.05191218	2.76160993	2.33792759
C	0.07404881	1.92599699	2.31631535
H	1.05056410	2.29221129	2.01125657
C	-3.93360005	-0.94968227	2.61647021
H	-3.46421905	-1.13096069	1.63555268
H	-5.02597655	-1.09533480	2.54155091
H	-3.53229125	-1.67897573	3.34880117
C	0.23186839	4.70180600	1.81914201
H	0.68821742	4.37124475	0.87071340
H	0.93662149	4.51118124	2.65453464
H	0.04502273	5.78772943	1.74952594
N	3.90136937	0.12556932	-0.44231586
N	1.76289303	2.00420448	-0.85924001
N	1.73998512	-1.41373940	-1.56514872
N	1.88558162	-0.28654910	1.54468344
C	4.12583451	1.48698160	-0.85299812
H	4.09970403	1.52275692	-1.95337065
H	5.13376890	1.85669378	-0.53572110
C	3.03745146	2.42388341	-0.33646477
H	3.04071511	2.39557344	0.77112539
H	3.25439743	3.46666406	-0.63453724
C	4.13901939	-0.90710751	-1.41828585
H	4.29666081	-1.86177142	-0.89989263
H	5.05484508	-0.70329689	-2.02501958
C	2.92823674	-1.11178866	-2.32996388
H	2.74893524	-0.19161607	-2.91278128
H	3.16661081	-1.93248353	-3.03481232
C	4.22441713	-0.19712490	0.92544780
H	4.32531103	0.74332031	1.48858996
H	5.20432613	-0.72887329	0.99912904
C	3.12743382	-1.02280497	1.58494618
H	3.04274655	-1.98787260	1.05451490
H	3.40452248	-1.25950839	2.62865998
C	0.97528387	2.96961425	-1.36732795
C	-0.24451468	2.43270885	-2.07292333
C	-1.50193042	2.95957224	-1.77371942
H	-1.57455693	3.74701391	-1.02470161
C	-2.64285717	2.36270931	-2.32678419
C	-2.52459463	1.32148441	-3.25450022
H	-3.42090102	0.83579610	-3.64335413
C	-1.25677064	0.80828065	-3.55917431
C	-0.11397430	1.36095980	-2.96809055
H	0.87588204	0.94157509	-3.12445760
C	-4.06963937	3.00621515	-0.57509529

H -3.68065804 4.00697926 -0.30213269
H -3.55477267 2.24381797 0.03434981
H -5.15490847 2.98558530 -0.37041262
C 0.00669864 -0.95835134 -4.50430759
H -0.19346237 -1.88185004 -5.07293503
H 0.42989388 -1.21332249 -3.51644422
H 0.75465819 -0.35233931 -5.05590053
C 1.67961077 -2.67677164 -1.08564567
C 0.39972744 -2.98625323 -0.34239535
C -0.83299053 -2.75870141 -0.96375665
H -0.84841798 -2.28064332 -1.93741414
C -2.02128769 -3.06520773 -0.28327320
C -1.96938893 -3.58244682 1.01709027
H -2.89200954 -3.77400160 1.56654343
C -0.73009187 -3.77175607 1.64711963
C 0.46166452 -3.50810840 0.95760914
H 1.43486857 -3.64054310 1.42254597
C -3.35257024 -2.07778157 -1.97838435
N -0.55814719 0.15913553 -0.23033799
C -1.73666612 0.19488421 -0.15193361
C -3.27256596 0.24147760 -0.04975373
H -3.68972491 1.13717313 0.38625995
H -3.74296671 -0.62565209 0.38980200
H -3.69919882 0.25236956 -1.04185541
*

[Co(NCH)L^{OCH2O}]$^{-}$

```
! UKS OLYP Opt RI def2-SVP def2/J D3 TightSCF TightOpt SlowConv
%SCF
 MaxIter 5000
end
%geom
 MaxIter 2000
end
%basis
newgto Co "def2-TZVP(-f)" end
end

* xyz -1 4
Co 13.29108661 8.04080322 8.48650268
H 16.91888467 2.63503534 4.87418325
O 12.16230559 8.68511407 4.56572430
O 17.36708478 7.83126557 9.45775862
O 9.99011967 7.65070431 11.10938523
O 15.85870615 4.36978703 4.96958535
O 17.18757016 3.43837930 6.67630016
O 14.72547394 2.83316579 10.71124221
O 12.37847791 2.79391760 10.68748862
O 9.83622978 3.97100659 6.78445059
O 11.04924436 3.57710221 4.79019704
C 13.54401545 3.21142686 11.37899387
H 13.51830788 4.29215976 11.56896118
H 13.55377645 2.65592971 12.33065326
C 10.44832058 3.02798020 5.93850009
```

H	11.16959594	2.41367232	6.50811514
H	9.65270859	2.37579017	5.53954586
N	13.35687072	10.25707900	8.87044899
N	13.31356379	8.73530875	6.56946746
N	15.08460396	8.17782852	9.50439307
N	11.52785220	8.31152947	9.51411584
C	13.91023144	10.81936617	7.64846750
H	14.98347441	10.58847844	7.62958987
H	13.79872823	11.92533205	7.62521247
C	13.25276834	10.17918859	6.44462389
H	12.20891755	10.52656118	6.32027058
H	13.76274685	10.51604788	5.52175876
C	12.77765736	8.12165131	5.48968752
C	13.01266777	6.64118271	5.34341303
C	14.31493775	6.13467167	5.29596255
H	15.16111386	6.78744006	5.51092202
C	14.53876133	4.77885562	5.02399640
C	13.45059120	3.91853267	4.79359935
H	13.59547496	2.85738432	4.58259806
C	12.14274066	4.42424945	4.93422137
C	11.92260489	5.78099603	5.15976138
H	10.90312226	6.16146482	5.21549032
C	14.21396290	10.38949782	10.03553838
H	13.63923242	10.04182007	10.90452227
H	14.48872129	11.45157075	10.21824138
C	15.44946033	9.53177759	9.88778257
H	16.15802813	9.96875407	9.15890536
H	16.01137542	9.53282706	10.84170187
C	16.19454097	7.41768602	9.36590627
C	16.04870833	5.93530718	9.13267208
C	15.40786938	5.11270265	10.07215401
H	14.86848114	5.59387256	10.88739291
C	15.41321624	3.72399399	9.89961337
C	16.09911674	3.15046329	8.81563457
H	16.06051237	2.06992649	8.66755145
C	16.65517376	3.98608306	7.83749042
C	16.66447585	5.37056367	8.00908673
H	17.13232645	6.00645425	7.25773194
C	11.98098733	10.65661528	9.11611065
H	11.46652629	10.67435158	8.14713169
H	11.93153834	11.68111418	9.54510180
C	11.28945159	9.65403371	10.01091409
H	11.62371789	9.75401671	11.06258626
H	10.20672049	9.87435921	10.04880475
C	10.75269996	7.41106194	10.15428334
C	10.82067442	5.98870406	9.66852719
C	10.28908285	5.61185437	8.43392234
H	9.89259172	6.36189544	7.74977678
C	10.34678770	4.26935086	8.03396849
C	10.92758087	3.31052316	8.87710586
H	11.02451878	2.26587785	8.58211522
C	11.57966961	3.73225250	10.05334972
C	11.44721489	5.04555731	10.49484245
H	11.88124389	5.38984445	11.43287766
C	16.30907153	3.19745577	5.60271526
H	15.44770811	2.57648807	5.91820145

```
N      13.27408041       6.10143717       8.16812899
C      13.35278427       4.95384023       7.98468015
H      13.42575651       3.88981733       7.81459103
*
```

[Co(NCH)L^{OMe}]$^{-}$

```
! UKS OLYP Opt RI def2-SVP def2/J D3 TightSCF TightOpt SlowConv
%SCF
 MaxIter 5000
end
%geom
 MaxIter 2000
end
%basis
newgto Co "def2-TZVP(-f)" end
end

* xyz -1 4
Co      1.47643326       0.17882642      -0.30841452
H      -2.62988911      -3.66207619      -3.33378097
O       1.19432235       4.23314070      -1.15799711
O       2.42235191      -3.54174314      -1.25524021
O       1.47925184      -1.12054342       3.64617481
O      -3.86842368       2.90105890      -1.76265121
O      -1.33680143      -0.24578818      -4.16587473
O      -3.29711757      -3.06112616      -1.44824112
O      -1.26939401      -3.69730287       2.73118875
O      -3.59477840       0.02610431       3.42012553
O      -1.30296160       3.83906502       1.94114936
H      -2.77277871      -1.89084165      -3.10749456
H      -4.25583864      -2.89925775      -3.21970402
C      -0.12694616      -3.92702031       3.54058784
H       0.47806294      -4.76198667       3.13675830
H      -0.51232319      -4.20958385       4.53355508
H       0.50347618      -3.02613953       3.63263759
C       1.24582496      -0.38717759       2.66012710
C      -0.00135497       0.45663295       2.67574229
C      -1.17849445      -0.15254424       3.12820351
H      -1.13362269      -1.19036925       3.44117115
C      -2.37777769       0.56558955       3.09657780
C      -2.39059165       1.90503124       2.68333367
H      -3.32628147       2.46727414       2.68276821
C      -1.19553891       2.52588049       2.29005660
C       0.00548850       1.79622845       2.26559752
H       0.92855694       2.23129578       1.89047571
C      -3.59967716      -1.28013963       3.95810304
H      -3.16630475      -2.02299349       3.26606605
H      -4.65448682      -1.53093105       4.15435262
H      -3.03135937      -1.32698149       4.90703390
C      -0.11099346       4.59563766       1.81758135
H       0.46780101       4.33677146       0.91592107
H       0.52498985       4.47672032       2.71602747
H      -0.42631040       5.64872555       1.73370397
N       3.90721805       0.14701156      -0.45184572
```

N	1.90439828	2.04016075	-0.89734848
N	1.81286757	-1.33173839	-1.61990332
N	2.01695030	-0.27776879	1.56030271
C	4.23210548	1.47072842	-0.95298121
H	4.18595107	1.44040284	-2.05179878
H	5.26572412	1.76932642	-0.66946615
C	3.21002995	2.48937745	-0.47334095
H	3.26618445	2.58207365	0.62969589
H	3.43134757	3.49021979	-0.88361648
C	4.20587246	-0.94469328	-1.36472105
H	4.29030526	-1.87432646	-0.78839583
H	5.17218559	-0.78037211	-1.89019804
C	3.05490239	-1.14145870	-2.34138864
H	2.95498693	-0.25095080	-2.98674253
H	3.28063116	-2.01473608	-2.97966170
C	4.32171382	-0.09992055	0.91882277
H	4.37449721	0.86516672	1.44188027
H	5.33302472	-0.56026906	0.95592689
C	3.29200774	-0.96128359	1.62710898
H	3.23867419	-1.95254157	1.13934327
H	3.58398973	-1.14034045	2.67566806
C	1.04164829	3.00000803	-1.28868054
C	-0.20829009	2.46862720	-1.93557798
C	-1.43775137	3.05004929	-1.60871807
H	-1.44076175	3.88959036	-0.91824771
C	-2.61325192	2.48152921	-2.11356390
C	-2.55662259	1.38511055	-2.98893761
H	-3.47886542	0.94022179	-3.36551465
C	-1.31293599	0.84093069	-3.33938009
C	-0.13346419	1.38471221	-2.81478180
H	0.83911649	0.94262825	-3.00682106
C	-3.95872356	3.99942504	-0.88061459
H	-3.53018189	4.91691065	-1.32931659
H	-3.43791138	3.81067866	0.07578380
H	-5.03195243	4.15767320	-0.68930278
C	-0.09257459	-0.85750804	-4.46120668
H	-0.32575057	-1.76485315	-5.03990115
H	0.45549153	-1.12797713	-3.54131638
H	0.54493995	-0.18935565	-5.07263271
C	1.62334741	-2.59276542	-1.16024111
C	0.27785759	-2.83725810	-0.51336884
C	-0.86874070	-2.81024164	-1.31803660
H	-0.77237433	-2.53462073	-2.36353242
C	-2.11923148	-3.08441765	-0.74839034
C	-2.22495858	-3.37349608	0.61769869
H	-3.20146615	-3.58302722	1.05667045
C	-1.06902943	-3.40715210	1.41399609
C	0.18999312	-3.14936736	0.84827386
H	1.09175223	-3.14085450	1.45548098
C	-3.22085597	-2.86366174	-2.84366704
N	-0.51319264	0.12493292	-0.16995988
C	-1.67276794	0.05240768	-0.09568694
H	-2.74846597	-0.01487149	-0.02678650

*

[Co(NCMe)L^{OMe}]$^{-}$, d(Co-N$_{axial}$) constrained to 2.25 Å

```
! UKS OLYP Opt RI def2-SVP def2/J D3 TightSCF TightOpt SlowConv
%SCF
 MaxIter 5000
end
%geom
        Constraints
        { B 0 35 2.25 C }
        end
end
%basis
newgto Co "def2-TZVP(-f)" end
end

* xyz -1 4
Co   1.53721887    0.17147683   -0.36922191
H   -2.89755766   -2.59038360   -2.84930735
O    1.16005459    4.20841322   -1.30691533
O    2.54918325   -3.56516149   -1.21008853
O    1.31592070   -1.08133497    3.65570199
O   -3.91430368    2.72199064   -1.95748522
O   -1.22893277   -0.28346721   -4.38830116
O   -3.26644534   -2.86385024   -0.80236008
O   -0.78535312   -4.19885614    2.94618079
O   -3.73435367    0.39164538    3.02535525
O   -1.02386348    4.10260296    2.05858043
H   -2.87268943   -1.09788177   -1.82799058
H   -4.42833021   -1.93230079   -2.17207931
C    0.42513846   -4.19854719    3.67721576
H    1.14053564   -4.94973851    3.28163068
H    0.15796892   -4.48196233    4.71038860
H    0.89820696   -3.19923992    3.67768530
C    1.12300132   -0.35487682    2.65197032
C   -0.07060545    0.56667331    2.64183114
C   -1.32486456    0.03971329    2.96296734
H   -1.40226318   -1.02674934    3.16433990
C   -2.45419027    0.86306886    2.88361510
C   -2.31863926    2.23120493    2.62207091
H   -3.20353758    2.86667651    2.55887990
C   -1.05191218    2.76160993    2.33792759
C    0.07404881    1.92599699    2.31631535
H    1.05056410    2.29221129    2.01125657
C   -3.93360005   -0.94968227    2.61647021
H   -3.46421905   -1.13096069    1.63555268
H   -5.02597655   -1.09533480    2.54155091
H   -3.53229125   -1.67897573    3.34880117
C    0.23186839    4.70180600    1.81914201
H    0.68821742    4.37124475    0.87071340
H    0.93662149    4.51118124    2.65453464
H    0.04502273    5.78772943    1.74952594
N    3.90136937    0.12556932   -0.44231586
N    1.76289303    2.00420448   -0.85924001
N    1.73998512   -1.41373940   -1.56514872
N    1.88558162   -0.28654910    1.54468344
C    4.12583451    1.48698160   -0.85299812
```

H	4.09970403	1.52275692	-1.95337065
H	5.13376890	1.85669378	-0.53572110
C	3.03745146	2.42388341	-0.33646477
H	3.04071511	2.39557344	0.77112539
H	3.25439743	3.46666406	-0.63453724
C	4.13901939	-0.90710751	-1.41828585
H	4.29666081	-1.86177142	-0.89989263
H	5.05484508	-0.70329689	-2.02501958
C	2.92823674	-1.11178866	-2.32996388
H	2.74893524	-0.19161607	-2.91278128
H	3.16661081	-1.93248353	-3.03481232
C	4.22441713	-0.19712490	0.92544780
H	4.32531103	0.74332031	1.48858996
H	5.20432613	-0.72887329	0.99912904
C	3.12743382	-1.02280497	1.58494618
H	3.04274655	-1.98787260	1.05451490
H	3.40452248	-1.25950839	2.62865998
C	0.97528387	2.96961425	-1.36732795
C	-0.24451468	2.43270885	-2.07292333
C	-1.50193042	2.95957224	-1.77371942
H	-1.57455693	3.74701391	-1.02470161
C	-2.64285717	2.36270931	-2.32678419
C	-2.52459463	1.32148441	-3.25450022
H	-3.42090102	0.83579610	-3.64335413
C	-1.25677064	0.80828065	-3.55917431
C	-0.11397430	1.36095980	-2.96809055
H	0.87588204	0.94157509	-3.12445760
C	-4.06963937	3.00621515	-0.57509529
H	-3.68065804	4.00697926	-0.30213269
H	-3.55477267	2.24381797	0.03434981
H	-5.15490847	2.98558530	-0.37041262
C	0.00669864	-0.95835134	-4.50430759
H	-0.19346237	-1.88185004	-5.07293503
H	0.42989388	-1.21332249	-3.51644422
H	0.75465819	-0.35233931	-5.05590053
C	1.67961077	-2.67677164	-1.08564567
C	0.39972744	-2.98625323	-0.34239535
C	-0.83299053	-2.75870141	-0.96375665
H	-0.84841798	-2.28064332	-1.93741414
C	-2.02128769	-3.06520773	-0.28327320
C	-1.96938893	-3.58244682	1.01709027
H	-2.89200954	-3.77400160	1.56654343
C	-0.73009187	-3.77175607	1.64711963
C	0.46166452	-3.50810840	0.95760914
H	1.43486857	-3.64054310	1.42254597
C	-3.35257024	-2.07778157	-1.97838435
N	-0.55814719	0.15913553	-0.23033799
C	-1.73666612	0.19488421	-0.15193361
C	-3.27256596	0.24147760	-0.04975373
H	-3.68972491	1.13717313	0.38625995
H	-3.74296671	-0.62565209	0.38980200
H	-3.69919882	0.25236956	-1.04185541
*			

S3.3. Input Files for Frequency Calculations

[CoL^{OCH2O}]$^{–}$

```
! UKS OLYP NumFreq RI def2-SVP def2/J D3 TightSCF SlowConv
%SCF
 MaxIter 5000
end
%geom
 MaxIter 2000
end
%basis
newgto Co "def2-TZVP(-f)" end
end

* xyz -1 4
 Co 13.21263719218008 8.26358075431312 8.43510744014107
 H 17.10867358971305 2.82592854586552 4.76062011551769
 O 12.17628296245105 8.65798022604168 4.52121508772336
 O 17.12971366735734 7.61170469787863 9.58227985291179
 O 10.38123617301136 7.67117748469370 11.31072869647654
 O 15.95227861633151 4.48645126083743 4.96684824005397
 O 17.16279306871823 3.45569304016633 6.66229646342268
 O 14.33853087194043 2.66666130562220 10.36551701978174
 O 12.15354274174289 2.80471445008872 11.13247591877253
 O 10.18996664307026 3.98364874359762 6.89570915029992
 O 11.22402996616377 3.51176358828052 4.87884848342548
 C 13.50832228797759 2.97470686868000 11.45042152718899
 H 13.71513853201388 3.99017604718210 11.83086730158863
 H 13.72118293493453 2.22770675321284 12.23380341476136
 C 10.78962668131761 2.98771460840158 6.10201911845148
 H 11.63325143908105 2.52656646627807 6.65004759187005
 H 10.02814098879987 2.22876864492770 5.84787808018986
 N 13.32248005107758 10.37269328501961 8.80867067254938
 N 13.28757821508910 8.81885866630734 6.53894699516201
 N 14.90758184001234 8.18565576372383 9.45205780430937
 N 11.51406175783266 8.43549949890335 9.44859100983260
 C 13.88107976758225 10.92126264238808 7.57792346241020
 H 14.95463331879730 10.68785274241631 7.57271664778129
 H 13.76872875828600 12.02579111872108 7.54139384641693
 C 13.22942416360702 10.25976973623068 6.37706933469644
 H 12.18659529777052 10.60706091100814 6.24142180563036
 H 13.74867399681866 10.57520101891854 5.45196289613135
 C 12.77775800952262 8.14669814332894 5.48019748374839
 C 13.04582838642445 6.66249314524851 5.43480432239931
 C 14.36665370707860 6.19482300481654 5.42296842278860
 H 15.19301714057675 6.88385403945662 5.59755321893788
 C 14.63608919582860 4.84876594514444 5.13842187175166
 C 13.58090860816580 3.94569976021175 4.95369810971997
 H 13.75006349940396 2.89318470006670 4.72389159966342
 C 12.26393906299913 4.41858879028760 5.02559402864715
 C 11.98693242433620 5.76886686660323 5.22150548041100
 H 10.95533059734747 6.11815256653277 5.21196900678101
 C 14.19392011146611 10.46223076084134 9.97410319509038
```

```
 H   13.59246952071945     10.17749388490840     10.84743514129707
 H   14.54604574931265     11.50361625354791     10.13423058033816
 C   15.36627731025531      9.50926406702295      9.84938763544938
 H   16.11481821859667      9.88978691681160      9.12800732687544
 H   15.90930470786721      9.46994489226491     10.81216006823308
 C   15.93971495224870      7.31438938980478      9.37094614862360
 C   15.67638970681224      5.87059601359176      9.00603596007244
 C   14.87960015853043      5.02276009068087      9.79629913582870
 H   14.27716626247861      5.46773809803527     10.58645671501096
 C   14.92334142287522      3.63305575245471      9.58040969179495
 C   15.67227530188706      3.10986752053608      8.51595373316413
 H   15.69036604207263      2.03071863784615      8.35071119663601
 C   16.41792154647768      3.97276211593412      7.71107414201522
 C   16.45068251245130      5.34037493852759      7.96484714437727
 H   17.08897714742572      5.98872807357692      7.36559707884470
 C   11.94790980903437     10.78745489390782      9.06586505298550
 H   11.43552427012396     10.83073269422830      8.09606554653035
 H   11.91376585361471     11.80321313640663      9.51375038902691
 C   11.25132031654537      9.77181164909930      9.94833830132894
 H   11.57158363718066      9.86465050915892     11.00397613028154
 H   10.16479744607893      9.98225730492766      9.97152190206963
 C   10.97806220590948      7.47703236301082     10.23730990539408
 C   11.13343320639431      6.06201148830705      9.74988637705366
 C   10.73862587386335      5.67822336396216      8.46217198978957
 H   10.39700447699431      6.42544821702566      7.74666243117895
 C   10.73718230179694      4.32030710158004      8.10703487467213
 C   11.22215368909267      3.35628814570198      9.00309239407427
 H   11.25804069428018      2.29401831710759      8.75940436564507
 C   11.65630908528377      3.76601913823820     10.26980217449145
 C   11.56849578299329      5.09650383590975     10.66679089939993
 H   11.83293806969951      5.40277259957693     11.67980357832008
 C   16.41307019464566      3.26903820382822      5.49430829100432
 H   15.56414525962991      2.58564880023569      5.68641498075652
*
```

[CoL^{OMe}]$^-$

```
! UKS OLYP NumFreq RI def2-SVP def2/J D3 TightSCF SlowConv
%SCF
 MaxIter 5000
end
%geom
 MaxIter 2000
end
%basis
newgto Co "def2-TZVP(-f)" end
end

* xyz -1 4
 Co  4.29643463223766      5.51610554205373      8.70281655783809
 H   -0.37026024890979     4.64535023827916      7.34862411923804
 O   4.00975646601747      9.60470231067582      8.14949903263349
 O   5.04940580742268      1.85799386210948      7.48965721071325
 O   3.51167182778241      3.63329554397319     12.21981702138533
 O   -1.08077748912096     8.86923210981993      7.49777245855497
 O   1.00850422573955      5.36112215946109      5.19540453464650
```

O	-0.42333512982664	3.65547877948383	9.18209987416093
O	2.07304614841131	0.35960894721613	11.36755182345178
O	-1.33142135112213	5.50196267068329	11.97087336928484
O	1.49764099581420	9.10705938867753	11.05534044086353
H	0.31832071123124	5.55411695947802	8.73064559575950
H	-1.44958807430388	5.31137046607579	8.62666737760243
C	3.34601885671363	0.02374037875819	11.87806013086168
H	3.91919128621877	-0.61982041199244	11.18112947912237
H	3.16997727571828	-0.53740644781503	12.81116405514560
H	3.93011396059809	0.93598774278930	12.09605733745151
C	3.49214906433995	4.55580208084345	11.38644605774042
C	2.31656330681736	5.50767413574386	11.34783612220636
C	1.04895005164749	5.01205492198387	11.68199281091936
H	0.96460238162247	3.95232433344606	11.90280574547185
C	-0.04933961966165	5.88226464398111	11.69997596193746
C	0.13079092898678	7.25146763015367	11.45584079060792
H	-0.72208008085818	7.92939537370529	11.51937559461737
C	1.41099821742259	7.75208876627190	11.18647999950923
C	2.50647872262558	6.87640830007472	11.09027081750028
H	3.50123724855129	7.23849789832322	10.84409544182273
C	-1.56703613010157	4.14156220137554	12.25938640329129
H	-1.32704310044641	3.50671194916930	11.39205247594064
H	-2.64114172154534	4.05707546470604	12.49275326340605
H	-0.98092963672705	3.79898207844088	13.13437031457463
C	2.77615236874736	9.70632657627964	11.16081129491765
H	3.39199577468992	9.54414848461179	10.26242650309528
H	3.31167493899440	9.33753411563711	12.05706325783148
H	2.59774431631736	10.78932394396859	11.26624618186364
N	6.51417505911237	5.38337323923523	8.69444807088600
N	4.65377623269339	7.39079571249812	8.30480000266290
N	4.47107742154859	4.09071342220823	7.32411614132779
N	4.47859075324272	4.83809175330933	10.50207665609137
C	6.96004385961415	6.72527548251764	8.32123731247688
H	7.00939432193958	6.76258762901392	7.22358507682809
H	7.97945996879741	6.92821931729070	8.71108641687918
C	5.96302945328501	7.77821889640695	8.77959105043220
H	5.97512053643909	7.85360056500645	9.88601438914671
H	6.24481307100050	8.77458159530282	8.39618004387255
C	6.83953575849679	4.36132206644436	7.69703284806832
H	6.86084185664489	3.38119779421659	8.18857115614497
H	7.84095481892212	4.54507752771027	7.25542159868155
C	5.73656077566846	4.29571847626902	6.64460679822853
H	5.69020099017027	5.24067390635943	6.07593349345669
H	5.96095424598184	3.46806090996719	5.94959213642415
C	6.82847249657348	4.99728657308206	10.07048675724940
H	6.87977567288675	5.91607771493861	10.67184560516969
H	7.81828717376792	4.49772853152545	10.12278112717116
C	5.72423069756741	4.11807724798285	10.64387847801582
H	5.69294326457617	3.14935462405268	10.11173030136247
H	5.92890177129679	3.88364633133475	11.70376702492544
C	3.79966874620140	8.38590696084388	7.98583122964354
C	2.48779751074811	7.92455338890891	7.40656035538536
C	1.34071950641263	8.66542440923425	7.71188490132041
H	1.45087668181643	9.52287782767898	8.37013424027977
C	0.10205907424176	8.26123404024158	7.19488966576797
C	0.02523507925039	7.16952143369069	6.31800000856423

H -0.93895929741254 6.86421939999086 5.90961156485174
C 1.18153932701263 6.43884344419665 6.01056145504822
C 2.41979718297328 6.80704483636757 6.56209844500127
H 3.32611042394263 6.24740499704790 6.34592069775869
C -1.04424352097202 9.93145707065032 8.42468883817169
H -0.49136099285823 10.80491513602147 8.02485347076628
H -0.57233829450870 9.63282401194099 9.37804923029752
H -2.09092073100432 10.22299499680320 8.60660571397887
C 2.16562061820733 4.64353693745343 4.80266778916898
H 1.81019949955556 3.78868809351016 4.20630784099391
H 2.74726822338957 4.27879160681333 5.66642152934052
H 2.82964774049157 5.27472619419536 4.17942710601795
C 4.27670047097721 2.80599264428851 7.72895959105603
C 3.05484058285029 2.56593744816296 8.57576127125884
C 1.87698454779293 3.31722177570993 8.43530879381392
H 1.84475343076976 4.13313515684354 7.71748922493873
C 0.76318020009879 2.99237135465523 9.22730189836283
C 0.84263285500068 1.95717148310050 10.17010271882584
H -0.00308468446844 1.73681914340844 10.82173298768654
C 2.04554590084415 1.26856769997750 10.35100484737983
C 3.14262209862409 1.54197451312985 9.52383781349466
H 4.08172268906156 0.99848726858939 9.59538937069777
C -0.47142297130845 4.85015627142892 8.42600546865873
*

[Co(NCMe)L^{OCH2O}]$^{-}$

! UKS OLYP NumFreq RI def2-SVP def2/J D3 TightSCF SlowConv
%SCF
 MaxIter 5000

end
%geom
 MaxIter 2000
end
%basis
newgto Co "def2-TZVP(-f)" end
end

* xyz -1 4
Co 13.29108660687319 8.04080321697397 8.48650267618197
H 16.91888467093055 2.63503534156303 4.87418324902343
O 12.16230559234748 8.68511406830656 4.56572429537063
O 17.36708477901589 7.83126557165852 9.45775861785907
O 9.99011967371549 7.65070430714562 11.10938522734219
O 15.85870614731083 4.36978703139409 4.96958534799065
O 17.18757016097224 3.43837930124638 6.67630015740456
O 14.72547394300706 2.83316579484437 10.71124220723431
O 12.37847790771424 2.79391760071805 10.68748861646842
O 9.83622978460347 3.97100658924582 6.78445059078415
O 11.04924436140505 3.57710220651993 4.79019704067818
C 13.54401545147307 3.21142686244263 11.37899387482847
H 13.51830787816496 4.29215976116830 11.56896117690892
H 13.55377644959581 2.65592971289202 12.33065326071256
C 10.44832058376760 3.02798019919175 5.93850008862470
H 11.16959593972967 2.41367232110371 6.50811513826020

H	9.65270859010269	2.37579016719543	5.53954585979955
N	13.35687071518856	10.25707899709341	8.87044898977516
N	13.31356378642283	8.73530875457108	6.56946745773517
N	15.08460395572909	8.17782851619577	9.50439306837051
N	11.52785219542076	8.31152946978527	9.51411583960559
C	13.91023143811120	10.81936616519253	7.64846749742542
H	14.98347441035763	10.58847844397501	7.62958986869556
H	13.79872823050642	11.92533204709835	7.62521247384211
C	13.25276834056990	10.17918858703700	6.44462389273069
H	12.20891755000695	10.52656117737143	6.32027057771777
H	13.76274685352529	10.51604787839336	5.52175875908312
C	12.77765736421731	8.12165131083395	5.48968752103371
C	13.01266776836602	6.64118271329188	5.34341303258990
C	14.31493775358777	6.13467166649134	5.29596255405343
H	15.16111385856509	6.78744006401994	5.51092202074374
C	14.53876132803162	4.77885562246924	5.02399640012137
C	13.45059119795014	3.91853266902173	4.79359935174710
H	13.59547496135714	2.85738431875463	4.58259805569465
C	12.14274066341137	4.42424944983387	4.93422137040044
C	11.92260489019702	5.78099603270399	5.15976138135720
H	10.90312225968004	6.16146482105121	5.21549032343673
C	14.21396289962846	10.38949782005421	10.03553837586515
H	13.63923241958833	10.04182007017591	10.90452226587064
H	14.48872129252004	11.45157075126894	10.21824137648439
C	15.44946033028810	9.53177759252876	9.88778256765201
H	16.15802813419836	9.96875406742807	9.15890536289149
H	16.01137541804618	9.53282706175886	10.84170186809723
C	16.19454097021903	7.41768601712678	9.36590627333838
C	16.04870833325814	5.93530718284475	9.13267207800221
C	15.40786937639838	5.11270264650123	10.07215400837087
H	14.86848114142654	5.59387255829835	10.88739290772837
C	15.41321624097500	3.72399399150348	9.89961337278941
C	16.09911673689631	3.15046329424453	8.81563456915753
H	16.06051237456060	2.06992648848038	8.66755144742769
C	16.65517376167272	3.98608306172596	7.83749041598354
C	16.66447584501800	5.37056367111768	8.00908672764478
H	17.13232644609290	6.00645424565767	7.25773194360711
C	11.98098732680829	10.65661528357287	9.11611064810836
H	11.46652629492870	10.67435157782439	8.14713168668796
H	11.93153834120013	11.68111417878699	9.54510180475610
C	11.28945158553001	9.65403370812873	10.01091409407159
H	11.62371788918345	9.75401671241474	11.06258625588129
H	10.20672048884786	9.87435920644058	10.04880475134667
C	10.75269995586053	7.41106193659687	10.15428334125181
C	10.82067441971473	5.98870405845620	9.66852719130938
C	10.28908285036567	5.61185437180006	8.43392233999045
H	9.89259171907242	6.36189544201605	7.74977678215835
C	10.34678769782038	4.26935085869151	8.03396849437678
C	10.92758087471307	3.31052316040941	8.87710585692038
H	11.02451877600759	2.26587785176571	8.58211521847938
C	11.57966960855760	3.73225250125411	10.05334972203188
C	11.44721488512107	5.04555731432078	10.49484245222305
H	11.88124388806697	5.38984444618226	11.43287766277146
C	16.30907153159710	3.19745577003977	5.60271526010137
H	15.44770810735455	2.57648807201419	5.91820145219454
N	13.27408041059400	6.10143716965697	8.16812898538712

```
 C   13.35278427353260      4.95384023193794      7.98468015381055
 C   13.47119130273805      3.54828969881204      7.70293350546173
 H   14.39934424691783      3.37705187907112      7.16919186284729
 H   12.64926922897456      3.24101512730532      7.06558557457316
 H   13.46698920377426      2.94895917299057      8.61357782071712
*
```

[Co(NCMe)L^{OMe}]$^{-}$

```
! UKS OLYP NumFreq RI def2-SVP def2/J D3 TightSCF SlowConv
%SCF
 MaxIter 5000

end
%geom
 MaxIter 2000
end
%basis
newgto Co "def2-TZVP(-f)" end
end

* xyz -1 4
 Co  1.47643326092012      0.17882641645214     -0.30841452000534
 H   -2.62988911039549     -3.66207619278997     -3.33378097022097
 O   1.19432234845939      4.23314070351389     -1.15799710520434
 O   2.42235190847365     -3.54174314406985     -1.25524021427047
 O   1.47925183719044     -1.12054341614558      3.64617480900433
 O   -3.86842368499518      2.90105890241709     -1.76265121426857
 O   -1.33680143280434     -0.24578817736725     -4.16587473216113
 O   -3.29711756688398     -3.06112615761557     -1.44824112251815
 O   -1.26939401256275     -3.69730286805219      2.73118875152193
 O   -3.59477839566175      0.02610431121591      3.42012553480584
 O   -1.30296159958241      3.83906501790465      1.94114935710127
 H   -2.77277871166362     -1.89084165084431     -3.10749455834488
 H   -4.25583864465045     -2.89925775362819     -3.21970402441622
 C   -0.12694616473205     -3.92702031263288      3.54058783553559
 H   0.47806293706212     -4.76198667000531      3.13675829965443
 H   -0.51232319485759     -4.20958384862448      4.53355508348905
 H   0.50347617920527     -3.02613952734612      3.63263759140043
 C   1.24582495655561     -0.38717758555137      2.66012709628229
 C   -0.00135496668134      0.45663294860256      2.67574229279363
 C   -1.17849444709885     -0.15254423823682      3.12820351251810
 H   -1.13362268797665     -1.19036925070560      3.44117115479589
 C   -2.37777768514689      0.56558954683507      3.09657780287064
 C   -2.39059165210978      1.90503123950586      2.68333366910185
 H   -3.32628147431134      2.46727413625599      2.68276820920654
 C   -1.19553890565232      2.52588049469986      2.29005659646469
 C   0.00548849991856      1.79622844522593      2.26559751611516
 H   0.92855694143707      2.23129577906972      1.89047570581436
 C   -3.59967715764175     -1.28013963034791      3.95810303951670
 H   -3.16630474568564     -2.02299349228334      3.26606604756123
 H   -4.65448682004312     -1.53093105324530      4.15435261763750
 H   -3.03135937135399     -1.32698149074596      4.90703389633065
 C   -0.11099346491474      4.59563765649159      1.81758134832995
```

H	0.46780101022564	4.33677146354653	0.91592107264621
H	0.52498984785087	4.47672032406767	2.71602746513836
H	-0.42631040232551	5.64872554777513	1.73370397181706
N	3.90721805406397	0.14701156311083	-0.45184572110927
N	1.90439827571957	2.04016075289383	-0.89734847654840
N	1.81286757240008	-1.33173838655576	-1.61990332202545
N	2.01695030037861	-0.27776878907555	1.56030271102852
C	4.23210548134863	1.47072842032996	-0.95298120774075
H	4.18595106954550	1.44040283541075	-2.05179878125989
H	5.26572411884336	1.76932642446058	-0.66946615433131
C	3.21002994780018	2.48937745273138	-0.47334095182041
H	3.26618445170565	2.58207364564804	0.62969588503128
H	3.43134756957888	3.49021979326102	-0.88361648074025
C	4.20587245524932	-0.94469327746208	-1.36472104600694
H	4.29030525577521	-1.87432646474688	-0.78839582718044
H	5.17218558831642	-0.78037211440749	-1.89019803569560
C	3.05490238578894	-1.14145870177356	-2.34138864191788
H	2.95498693124373	-0.25095079524871	-2.98674252766530
H	3.28063116136733	-2.01473608017114	-2.97966170174549
C	4.32171381844107	-0.09992055146034	0.91882276540221
H	4.37449720977546	0.86516672305289	1.44188027292578
H	5.33302471952548	-0.56026905682700	0.95592688673191
C	3.29200773786288	-0.96128359321703	1.62710898265359
H	3.23867419161549	-1.95254157194224	1.13934327317143
H	3.58398972588515	-1.14034044591325	2.67566805539910
C	1.04164829452597	3.00000803186592	-1.28868053901386
C	-0.20829008712354	2.46862719797301	-1.93557798379672
C	-1.43775137033306	3.05004928503366	-1.60871806728703
H	-1.44076174984126	3.88959035653007	-0.91824771245809
C	-2.61325191532340	2.48152920563238	-2.11356389667442
C	-2.55662258956948	1.38511054939092	-2.98893761093304
H	-3.47886541917786	0.94022178949889	-3.36551465068763
C	-1.31293598728825	0.84093068847553	-3.33938008554251
C	-0.13346419009395	1.38471221425221	-2.81478180391598
H	0.83911649497235	0.94262825110620	-3.00682105638355
C	-3.95872355635792	3.99942503579266	-0.88061458941220
H	-3.53018189437820	4.91691065270367	-1.32931658544304
H	-3.43791138066849	3.81067866250138	0.07578380296897
H	-5.03195242628024	4.15767319693700	-0.68930277899285
C	-0.09257459133032	-0.85750803569958	-4.46120668317366
H	-0.32575057052020	-1.76485315007635	-5.03990115031991
H	0.45549152624611	-1.12797713037828	-3.54131637723890
H	0.54493994594393	-0.18935564687262	-5.07263270817318
C	1.62334741180273	-2.59276541957508	-1.16024110727843
C	0.27785759415407	-2.83725810148384	-0.51336884311134
C	-0.86874070351679	-2.81024163915326	-1.31803660182230
H	-0.77237433404381	-2.53462073475641	-2.36353242280878
C	-2.11923147643186	-3.08441764617304	-0.74839033586726
C	-2.22495857948598	-3.37349608391647	0.61769868623313
H	-3.20146615064207	-3.58302722427670	1.05667044873921
C	-1.06902942759633	-3.40715210437099	1.41399608751573
C	0.18999311956471	-3.14936735565753	0.84827385799744
H	1.09175222531487	-3.14085449716562	1.45548098003646
C	-3.22085597030279	-2.86366174126632	-2.84366704223174
N	-0.51319264006736	0.12493291694851	-0.16995987945871
C	-1.67276794326569	0.05240767574439	-0.09568694257592

```
 C   -3.11197367770347    -0.04743837838028    -0.01261603980094
 H   -3.56364235786760     0.94873098727966    -0.10966048501067
 H   -3.39979355542374    -0.47937500977492     0.95427328394611
 H   -3.48603409768923    -0.69362655412867    -0.81815703062444
*
```

[Co(NCH)L^{OCH2O}]$^{-}$

```
! UKS OLYP NumFreq RI def2-SVP def2/J D3 TightSCF SlowConv
%SCF
 MaxIter 5000

end
%geom
 MaxIter 2000
end
%basis
newgto Co "def2-TZVP(-f)" end
end


* xyz -1 4
 Co  1.47643326092012     0.17882641645214    -0.30841452000534
 H   -2.62988911039549    -3.66207619278997    -3.33378097022097
 O   1.19432234845939     4.23314070351389    -1.15799710520434
 O   2.42235190847365    -3.54174314406985    -1.25524021427047
 O   1.47925183719044    -1.12054341614558     3.64617480900433
 O   -3.86842368499518     2.90105890241709    -1.76265121426857
 O   -1.33680143280434    -0.24578817736725    -4.16587473216113
 O   -3.29711756688398    -3.06112615761557    -1.44824112251815
 O   -1.26939401256275    -3.69730286805219     2.73118875152193
 O   -3.59477839566175     0.02610431121591     3.42012553480584
 O   -1.30296159958241     3.83906501790465     1.94114935710127
 H   -2.77277871166362    -1.89084165084431    -3.10749455834488
 H   -4.25583864465045    -2.89925775362819    -3.21970402441622
 C   -0.12694616473205    -3.92702031263288     3.54058783553559
 H   0.47806293706212    -4.76198667000531     3.13675829965443
 H   -0.51232319485759    -4.20958384862448     4.53355508348905
 H   0.50347617920527    -3.02613952734612     3.63263759140043
 C   1.24582495655561    -0.38717758555137     2.66012709628229
 C   -0.00135496668134     0.45663294860256     2.67574229279363
 C   -1.17849444709885    -0.15254423823682     3.12820351251810
 H   -1.13362268797665    -1.19036925070560     3.44117115479589
 C   -2.37777768514689     0.56558954683507     3.09657780287064
 C   -2.39059165210978     1.90503123950586     2.68333366910185
 H   -3.32628147431134     2.46727413625599     2.68276820920654
 C   -1.19553890565232     2.52588049469986     2.29005659646469
 C   0.00548849991856     1.79622844522593     2.26559751611516
 H   0.92855694143707     2.23129577906972     1.89047570581436
 C   -3.59967715764175    -1.28013963034791     3.95810303951670
 H   -3.16630474568564    -2.02299349228334     3.26606604756123
 H   -4.65448682004312    -1.53093105324530     4.15435261763750
 H   -3.03135937135399    -1.32698149074596     4.90703389633065
 C   -0.11099346491474     4.59563765649159     1.81758134832995
 H   0.46780101022564     4.33677146354653     0.91592107264621
```

H	0.52498984785087	4.47672032406767	2.71602746513836
H	-0.42631040232551	5.64872554777513	1.73370397181706
N	3.90721805406397	0.14701156311083	-0.45184572110927
N	1.90439827571957	2.04016075289383	-0.89734847654840
N	1.81286757240008	-1.33173838655576	-1.61990332202545
N	2.01695030037861	-0.27776878907555	1.56030271102852
C	4.23210548134863	1.47072842032996	-0.95298120774075
H	4.18595106954550	1.44040283541075	-2.05179878125989
H	5.26572411884336	1.76932642446058	-0.66946615433131
C	3.21002994780018	2.48937745273138	-0.47334095182041
H	3.26618445170565	2.58207364564804	0.62969588503128
H	3.43134756957888	3.49021979326102	-0.88361648074025
C	4.20587245524932	-0.94469327746208	-1.36472104600694
H	4.29030525577521	-1.87432646474688	-0.78839582718044
H	5.17218558831642	-0.78037211440749	-1.89019803569560
C	3.05490238578894	-1.14145870177356	-2.34138864191788
H	2.95498693124373	-0.25095079524871	-2.98674252766530
H	3.28063116136733	-2.01473608017114	-2.97966170174549
C	4.32171381844107	-0.09992055146034	0.91882276540221
H	4.37449720977546	0.86516672305289	1.44188027292578
H	5.33302471952548	-0.56026905682700	0.95592688673191
C	3.29200773786288	-0.96128359321703	1.62710898265359
H	3.23867419161549	-1.95254157194224	1.13934327317143
H	3.58398972588515	-1.14034044591325	2.67566805539910
C	1.04164829452597	3.00000803186592	-1.28868053901386
C	-0.20829008712354	2.46862719797301	-1.93557798379672
C	-1.43775137033306	3.05004928503366	-1.60871806728703
H	-1.44076174984126	3.88959035653007	-0.91824771245809
C	-2.61325191532340	2.48152920563238	-2.11356389667442
C	-2.55662258956948	1.38511054939092	-2.98893761093304
H	-3.47886541917786	0.94022178949889	-3.36551465068763
C	-1.31293598728825	0.84093068847553	-3.33938008554251
C	-0.13346419009395	1.38471221425221	-2.81478180391598
H	0.83911649497235	0.94262825110620	-3.00682105638355
C	-3.95872355635792	3.99942503579266	-0.88061458941220
H	-3.53018189437820	4.91691065270367	-1.32931658544304
H	-3.43791138066849	3.81067866250138	0.07578380296897
H	-5.03195242628024	4.15767319693700	-0.68930277899285
C	-0.09257459133032	-0.85750803569958	-4.46120668317366
H	-0.32575057052020	-1.76485315007635	-5.03990115031991
H	0.45549152624611	-1.12797713037828	-3.54131637723890
H	0.54493994594393	-0.18935564687262	-5.07263270817318
C	1.62334741180273	-2.59276541957508	-1.16024110727843
C	0.27785759415407	-2.83725810148384	-0.51336884311134
C	-0.86874070351679	-2.81024163915326	-1.31803660182230
H	-0.77237433404381	-2.53462073475641	-2.36353242280878
C	-2.11923147643186	-3.08441764617304	-0.74839033586726
C	-2.22495857948598	-3.37349608391647	0.61769868623313
H	-3.20146615064207	-3.58302722427670	1.05667044873921
C	-1.06902942759633	-3.40715210437099	1.41399608751573
C	0.18999311956471	-3.14936735565753	0.84827385799744
H	1.09175222531487	-3.14085449716562	1.45548098003646
C	-3.22085597030279	-2.86366174126632	-2.84366704223174
N	-0.51319264006736	0.12493291694851	-0.16995987945871
C	-1.67276794326569	0.05240767574439	-0.09568694257592
C	-3.11197367770347	-0.04743837838028	-0.01261603980094

```
 H   -3.56364235786760     0.94873098727966    -0.10966048501067
 H   -3.39979355542374    -0.47937500977492     0.95427328394611
 H   -3.48603409768923    -0.69362655412867    -0.81815703062444
*
```

$[Co(NCH)L^{OMe}]^{-}$

```
! UKS OLYP NumFreq RI def2-SVP def2/J D3 TightSCF SlowConv
%SCF
 MaxIter 5000

end
%geom
 MaxIter 2000
end
%basis
newgto Co "def2-TZVP(-f)" end
end


* xyz -1 4
 Co  1.50183546558163     0.17148135737094    -0.31673411191805
 H   -2.84617745707353    -3.37551928962173    -3.17396967462552
 O   1.18402534712086     4.21315555425226    -1.16696723028735
 O   2.42107015692514    -3.55623387558102    -1.23049192373469
 O   1.45132897300936    -1.10055086190117     3.64629771409620
 O   -3.87548042541181     2.75779188646265    -1.71328644699060
 O   -1.29030924135458    -0.27076445458015    -4.20463108955309
 O   -3.33384476017717    -2.74274468481355    -1.24447435649562
 O   -1.17673568665674    -3.74055440970482     2.78898502594586
 O   -3.65244221243255    -0.00659795189111     3.19355590583690
 O   -1.31663377439244     3.82862165751890     1.85326995950145
 H   -2.78788066636398    -1.60652475676802    -2.92105365312166
 H   -4.37313721729336    -2.44825591850060    -2.95235850610441
 C   -0.01264549745946    -3.98888600822595     3.56016869315153
 H   0.57093042218000    -4.82779462300980     3.13330277200047
 H   -0.36951275496555    -4.27403096950464     4.56287847373526
 H   0.62504303567782    -3.09145338358387     3.63695030607443
 C   1.22498394787835    -0.38228870125697     2.64910872110722
 C   -0.03067689755848     0.44894240297864     2.62805667749631
 C   -1.22227124037792    -0.17186056377630     3.02296837633886
 H   -1.18463078292412    -1.20961905791497     3.33869421118536
 C   -2.42748409823878     0.53335176002548     2.91898826434482
 C   -2.42862689815792     1.87505559751792     2.50553731275235
 H   -3.36778869251536     2.42956705586945     2.46550844895937
 C   -1.21917517574154     2.51152508750503     2.18967298748343
 C   -0.01375501518687     1.79085567279497     2.22326029905706
 H   0.92382186206700     2.23397323920872     1.89657612501508
 C   -3.67605049848447    -1.29437870054907     3.77128371413817
 H   -3.19753722526189    -2.05202914935478     3.12928131858826
 H   -4.73790417363311    -1.55148472465867     3.91508189263098
 H   -3.16226182092524    -1.30639717424931     4.75220766173010
 C   -0.12360091217620     4.59187457362788     1.79892897847422
 H   0.49649466681055     4.34619325172014     0.92119159514898
```

H	0.46805267023163	4.46222610300696	2.72572329421576
H	-0.43969737819624	5.64469218753316	1.71476473882964
N	3.90950852097663	0.13759263982239	-0.44621468916918
N	1.91723829878585	2.02747527587767	-0.91669560127501
N	1.82356813670426	-1.34766563951914	-1.61882818554217
N	2.01290545872415	-0.27532998164969	1.55902158446902
C	4.24241042477377	1.45379980011005	-0.96472603190714
H	4.19967008828418	1.40754709051780	-2.06300557030442
H	5.27637607365636	1.75039914469636	-0.68168209099424
C	3.22319479844297	2.48399287777947	-0.50311651903658
H	3.28115163231534	2.59950032777318	0.59778702323048
H	3.44623879147404	3.47613177657540	-0.93331806573101
C	4.21402089356449	-0.96684185497371	-1.34329883007564
H	4.28803844492412	-1.88965343222654	-0.75469704659799
H	5.18696544503269	-0.81187630660491	-1.85885364180919
C	3.07296105779955	-1.17031272643104	-2.33069970784075
H	2.98317461267759	-0.28685869344026	-2.98710673565738
H	3.30187644862453	-2.05170304896031	-2.95627734983776
C	4.32013755317151	-0.08978698278369	0.93014240231322
H	4.36444637882657	0.88307642932329	1.43919435503326
H	5.33393255910496	-0.54324569520929	0.97661088885069
C	3.29242768468338	-0.94708702799708	1.64671537229744
H	3.24864753302604	-1.94730882956906	1.17683435846680
H	3.57947004856457	-1.10504258408692	2.70016515504785
C	1.04027918776387	2.97993714613252	-1.29800002590821
C	-0.20781039801692	2.43281903545802	-1.93679467572853
C	-1.44780652151431	2.97498291358958	-1.58380940130040
H	-1.46713783616939	3.79777304584976	-0.87368935125844
C	-2.61557396078347	2.37593133866947	-2.07533215161704
C	-2.53823508111223	1.29569214715854	-2.97146894934599
H	-3.45418000653492	0.84003764512758	-3.35079727511418
C	-1.28466831839140	0.79794349415675	-3.35474867373199
C	-0.11463107152950	1.36421138716619	-2.83464097223322
H	0.86704185035138	0.95275105080172	-3.04720445414734
C	-3.99130636677352	3.82177508822347	-0.79427681297553
H	-3.60122542523786	4.76932922556553	-1.21574003311347
H	-3.45009525059855	3.62066199913689	0.14743911319278
H	-5.06652440192604	3.93656789489987	-0.58364492713637
C	-0.03794263445101	-0.85557562403710	-4.51685489829688
H	-0.25868413570534	-1.75135975814435	-5.11840217502757
H	0.51510527689449	-1.14212643235957	-3.60487600717404
H	0.58929062204719	-0.16322448927868	-5.11211783727567
C	1.62420268807586	-2.60460232600664	-1.14707311916021
C	0.28474410089701	-2.82507506822895	-0.48166131877470
C	-0.88867168004302	-2.69260974180086	-1.23513551483293
H	-0.82118151473023	-2.35029389140707	-2.26252020450047
C	-2.12951449625364	-2.90748304911592	-0.61790993947144
C	-2.19396193053662	-3.26659940289514	0.73413645665548
H	-3.16414251005233	-3.43786239819292	1.20184050166845
C	-1.01167572562117	-3.40854861276074	1.47739490948841
C	0.23455609371222	-3.19992192608384	0.86708300799796
H	1.16251848627674	-3.27934342427327	1.42742071735226
C	-3.31999677419812	-2.52859025873626	-2.63871120160171
N	-0.46445054672040	0.13954953360248	-0.18950542939673
C	-1.62165300611124	0.05381633140707	-0.11459771463790
H	-2.69024847166809	-0.04119158059467	-0.04534748153177

*

[Co(NCMe)L^{OMe}]$^{-}$, d(Co-N$_{axial}$) constrained to 2.25 Å

```
! UKS OLYP NumFreq RI def2-SVP def2/J D3 TightSCF SlowConv
%SCF
 MaxIter 5000

end
%geom
 MaxIter 2000
end
%basis
newgto Co "def2-TZVP(-f)" end
end


* xyz -1 4
 Co  1.57368857505710   0.18642637800704  -0.32375370777254
 H  -2.61774228784410  -3.64822634733017  -3.34031760283265
 O   1.18376846010903   4.25174062204911  -1.15114220933358
 O   2.43696151103460  -3.54235659199345  -1.17069234743864
 O   1.46215016531138  -1.14978514055870   3.63273294201621
 O  -3.87561140175531   2.90513832956210  -1.74592175926836
 O  -1.34389444656351  -0.22618204247874  -4.16845816398480
 O  -3.29717553573537  -3.05657503318598  -1.45611493392757
 O  -1.29353405914277  -3.71987101616798   2.73037933651553
 O  -3.60546379973876   0.01510157286262   3.41707135395743
 O  -1.30755380034291   3.83255906458570   1.96041791186617
 H  -2.76641454356271  -1.87838532719104  -3.10829727809066
 H  -4.24612487232615  -2.88961665190756  -3.23233423693067
 C  -0.15553905809425  -3.95006323515024   3.54583381220599
 H   0.45209264771082  -4.78443304375988   3.14464120205302
 H  -0.54622743554556  -4.23366972750043   4.53644948933657
 H   0.47414351731758  -3.04905202479783   3.64120793859801
 C   1.23658542272816  -0.39803060744047   2.65867225611413
 C  -0.01123941609077   0.44402360350721   2.67576093911509
 C  -1.18949692598952  -0.16620101513089   3.12408016750612
 H  -1.14602850324044  -1.20605680315123   3.43055287479259
 C  -2.38774382279934   0.55418873415557   3.09725750304435
 C  -2.39781777109993   1.89646624897805   2.69325259592110
 H  -3.33250356060119   2.46036474117106   2.69614144059681
 C  -1.20185228580006   2.51733581797013   2.30271637221062
 C  -0.00216571827937   1.78597105915593   2.27368930210925
 H   0.92153269259850   2.21989534856021   1.89864259121391
 C  -3.61310336537734  -1.29358115524479   3.94854505547476
 H  -3.17955748370838  -2.03402715190154   3.25407439258476
 H  -4.66860803030830  -1.54403441205959   4.14161886783834
 H  -3.04655290655557  -1.34615980662107   4.89831198015441
 C  -0.11433777342616   4.58641468602469   1.83453018247217
 H   0.46116039491091   4.32788351192014   0.93076638900681
 H   0.52421093065929   4.46458771721478   2.73082790602134
 H  -0.42733527878034   5.64037613209064   1.75309118193501
 N   3.81932390329858   0.15241689776085  -0.45964013010950
 N   1.91638821187214   2.06453400795370  -0.93174390950058
```

N	1.80888682179733	-1.35744283633128	-1.64455020249817
N	2.02685429869045	-0.26238999761510	1.57627680693225
C	4.20850520148933	1.45198070271355	-1.00324321317716
H	4.15087255455078	1.39172613107788	-2.09981543363010
H	5.25683438231120	1.69767378376837	-0.73121014747206
C	3.23026884415719	2.51854148041095	-0.54513155299621
H	3.30903763779706	2.66105462785825	0.55162979334695
H	3.46581518156205	3.49509141221323	-1.00359677882500
C	4.17677204416353	-0.96316181575582	-1.33679077513751
H	4.24516061598967	-1.87710256313307	-0.73476390370196
H	5.16365824868916	-0.79037949277778	-1.81601736283171
C	3.06566996055447	-1.19381779745533	-2.34681385177073
H	2.98194195265066	-0.32624582374228	-3.02533673127613
H	3.31213016211813	-2.08868709433903	-2.94651256708026
C	4.29634101039308	-0.05067940061193	0.90764377427172
H	4.33133758122066	0.92841779943360	1.40471588021488
H	5.32224929614015	-0.47399792651647	0.90833079137758
C	3.31211080704116	-0.92155470513373	1.66161827979570
H	3.27722290135249	-1.93189324154407	1.21236761936504
H	3.62261087770895	-1.05339209217535	2.71217724292427
C	1.03725264135074	3.01939101960492	-1.29589489625654
C	-0.21473173956578	2.48560564706563	-1.93580824393599
C	-1.44472069302664	3.06175704165381	-1.60126985902512
H	-1.44719818782994	3.89889082007060	-0.90776447018848
C	-2.62059708354676	2.49168796291359	-2.10363998000792
C	-2.56372441523996	1.39946367281043	-2.98421766410838
H	-3.48601888661062	0.95254915526689	-3.35832995039806
C	-1.31988240766086	0.85942944119423	-3.34042830319155
C	-0.14007839761772	1.40524436522313	-2.81908004109876
H	0.83278159205493	0.96441215118173	-3.01288951527870
C	-3.96635648485687	3.99691544367484	-0.85616625075643
H	-3.54389304238711	4.91957446073752	-1.30011610710053
H	-3.44023302080729	3.80383106547918	0.09651356176547
H	-5.03929062853746	4.14937577369205	-0.65849602988632
C	-0.09995824636590	-0.83999536838134	-4.46142447506911
H	-0.33403532617864	-1.74858851137537	-5.03790379353600
H	0.44778811714437	-1.10877435984213	-3.54033310769380
H	0.53800195801287	-0.17421894417888	-5.07513795587463
C	1.62382283240422	-2.60050859475681	-1.13299814087488
C	0.27222008417310	-2.83934679402871	-0.49970751945539
C	-0.86980016151815	-2.80382418847045	-1.31044406614121
H	-0.76662073897403	-2.51933366992292	-2.35288199997294
C	-2.12338956005216	-3.08343466770182	-0.75000431336201
C	-2.23653908348707	-3.38306381883167	0.61320295078442
H	-3.21566871123075	-3.59516528121779	1.04508002700922
C	-1.08524045254584	-3.42235695272641	1.41596252660226
C	0.17696581600080	-3.16276545916212	0.85877053580549
H	1.07667429840677	-3.16162082588876	1.46910447605189
C	-3.21338981966856	-2.85324213176869	-2.85019355338122
N	-0.44011321162799	0.13250715376234	-0.18513833585362
C	-1.59880800528246	0.05417638264252	-0.10703971828192
C	-3.03766771434543	-0.04727649531306	-0.01880864912903
H	-3.48713046001767	0.95357907730580	-0.05866756308976
H	-3.31973193964402	-0.53036761269926	0.92503336983707
H	-3.42022223119968	-0.64669094831551	-0.85540240820989

*

MeCN (geometry optimization and numerical frequency)

```
! RKS OLYP Opt RI def2-SVP def2/J D3 TightSCF TightOpt SlowConv NumFreq
%SCF
 MaxIter 5000

end
%geom
 MaxIter 2000
end


* xyz 0 1

  N   13.27408041059400     6.10143716965697     8.16812898538712
  C   13.35278427353260     4.95384023193794     7.98468015381055
  C   13.47119130273805     3.54828969881204     7.70293350546173
  H   14.39934424691783     3.37705187907112     7.16919186284729
  H   12.64926922897456     3.24101512730532     7.06558557457316
  H   13.46698920377426     2.94895917299057     8.61357782071712
*
```

HCN (geometry optimization and numerical frequency)

```
! RKS OLYP Opt RI def2-SVP def2/J D3 TightSCF TightOpt SlowConv NumFreq
%SCF
 MaxIter 5000

end
%geom
 MaxIter 2000
end

* xyz 0 1

N     0    0   0
C     0    0   1.20
H     0    0   2.15
*
```

S6.5. Input Files for Relaxed Surface Scans of [Co(NCR)L^{OCH2O}]$^{–}$ (R = Me, H)

[Co(NCMe)L^{OCH2O}]$^{–}$

```
! UKS OLYP Opt RI def2-SVP def2/J D3 TightSCF SlowConv XYZFile
%SCF
 MaxIter 5000

end
%geom
        Scan
        B 0 71 = 1.95, 5.55, 13
        end
        Constraints
```

```
    { A 17 0 71 C }
    end
end
%basis
newgto Co "def2-TZVP(-f)" end
end


* xyz -1 4

Co      0.00000000      0.00000000      0.00000000
H       3.65129469     -2.72624104      5.88793289
O      -1.16917053     -3.96165917      0.00911488
O       4.08617299      0.95078650      0.01416030
O      -3.27400217      2.68493072     -0.01138462
O       2.57436162     -2.90215715      4.16987812
O       3.92736455     -1.08079290      4.80098090
O       1.50656815      3.02377409      4.76586437
O      -0.84002384      3.03060211      4.82874862
O      -3.42780998     -0.98540718      4.32149894
O      -2.22814630     -2.90183181      5.02273780
C       0.32713917      3.63347807      4.29466778
H       0.29206645      3.64622041      3.19769942
H       0.35079358      4.66236786      4.68856396
C      -2.81348555     -1.67371307      5.38377323
H      -2.08108690     -1.01953517      5.89143170
H      -3.60583360     -1.95368718      6.09883533
N       0.04651960      0.01937541     -2.24968468
N      -0.00118862     -2.00431273     -0.37496167
N       1.80076861      0.96395008     -0.31561671
N      -1.75694348      0.98813635     -0.41834586
C       0.58352838     -1.28309271     -2.61136422
H       1.65886455     -1.27527446     -2.38976329
H       0.46056562     -1.48395955     -3.69794922
C      -0.07777005     -2.36064745     -1.77896730
H      -1.12614284     -2.52895787     -2.09256264
H       0.42074539     -3.33102236     -1.96628314
C      -0.54013795     -2.96497919      0.41011759
C      -0.29133026     -2.87202274      1.89263814
C       1.01557345     -2.85010892      2.38885843
H       1.85688314     -2.75230130      1.70259329
C       1.25083769     -2.90124658      3.76883502
C       0.16953672     -2.97821862      4.66450022
H       0.32339344     -3.01610133      5.74451952
C      -1.14213276     -2.90800955      4.15401917
C      -1.37412072     -2.90287134      2.78061299
H      -2.39690651     -2.89909131      2.40497756
C       0.91226733      1.13886147     -2.57649791
H       0.34864970      2.05848005     -2.36921843
H       1.17776098      1.14439543     -3.65647659
C       2.15511487      1.11933964     -1.71689420
H       2.85286034      0.32215089     -2.03614346
H       2.72522314      2.05472880     -1.87731669
C       2.91715298      0.93905596      0.44728669
C       2.78441866      0.95039406      1.94907410
C       2.16014734      2.01714112      2.61414184
```

H	1.62295892	2.74917604	2.01195242
C	2.17814981	2.07166336	4.01235994
C	2.86045308	1.08790856	4.74795205
H	2.83158003	1.11711914	5.83849861
C	3.39948290	-0.01823197	4.07703795
C	3.39616298	-0.07315076	2.68301812
H	3.85099159	-0.92227098	2.17321346
C	-1.33118593	0.21111662	-2.67181828
H	-1.85416609	-0.74265764	-2.52790382
H	-1.38735678	0.46906302	-3.75181137
C	-2.00470239	1.26343180	-1.82148126
H	-1.66238473	2.28158114	-2.09339200
H	-3.08925371	1.27618144	-2.03565768
C	-2.51731268	1.77350803	0.37320149
C	-2.43905555	1.52377315	1.85481878
C	-2.97744283	0.37194934	2.43126956
H	-3.38746021	-0.42052637	1.80541817
C	-2.90952557	0.19405649	3.82027999
C	-2.31171964	1.17534287	4.62480282
H	-2.20669937	1.05239995	5.70255791
C	-1.65380821	2.26108498	4.01247807
C	-1.79581174	2.48545539	2.64619751
H	-1.35721270	3.35090366	2.15076331
C	3.04210958	-2.09220147	5.22022411
H	2.18987977	-1.67158321	5.78931460
N	-0.00000000	-0.00000000	1.96539861
C	0.00000309	0.00000014	3.13022756
C	0.02077809	-0.05656423	4.56735755
H	0.92448826	-0.56593124	4.88283114
H	-0.83250889	-0.62864876	4.91492991
H	-0.00343687	0.93757071	5.01412053

*

[Co(NCH)L^{OCH2O}]$^{-}$

```
! UKS OLYP Opt RI def2-SVP def2/J D3 TightSCF SlowConv XYZFile
%SCF
 MaxIter 5000

end
%geom
	Scan
	B 0 71 = 1.95, 5.55, 13
	end
	Constraints
	{ A 17 0 71 C }
	{ A 0 71 72 C }
	end
end
%basis
newgto Co "def2-TZVP(-f)" end
end

* xyz -1 4
```

Co	13.27586278539648	8.10046899306029	8.39879715970994
H	16.70165359604756	2.53495873282038	4.93028693224302
O	12.27121210773457	8.80514876376814	4.41877931069806
O	17.29215701724333	7.70745397359064	9.65203746886560
O	10.14981216994257	7.58274409299680	11.16010218226012
O	15.80650690743713	4.36925430289034	4.99770884178215
O	17.02578074835958	3.37952402988934	6.71437990930780
O	14.72128258866113	2.76691679867762	10.82744483930252
O	12.39389546830200	2.73273805162221	10.81085085757474
O	9.93642951712035	3.86463777304036	6.84970917602105
O	11.02168485675137	3.68403825879552	4.79618672709711
C	13.54838934018600	3.07799244396566	11.53294606383063
H	13.52889796165944	4.13491853815425	11.83915587553621
H	13.56308129421379	2.42889204977948	12.42289507324089
C	10.61710438838205	3.01595745468888	5.95788509628190
H	11.48481149364741	2.54857838998771	6.45979012644549
H	9.90628789388759	2.24245212104822	5.61842409653264
N	13.33877519009863	10.28999339368740	8.82782723260417
N	13.29278717127211	8.82350421930411	6.49170174882411
N	15.04278363601917	8.17951891820316	9.44776581748099
N	11.54336538587169	8.30859470084497	9.46603800934183
C	13.87560900235573	10.88822413238145	7.61477986655446
H	14.95219312594928	10.67267756423449	7.58710441345035
H	13.74926028438887	11.99276163760024	7.61906563596320
C	13.22455379981543	10.26922406312868	6.39526629798970
H	12.17825589118981	10.61315968714858	6.27753284271488
H	13.73282343458984	10.62949513548871	5.48096108807790
C	12.81980865770346	8.22592648519205	5.37417987625947
C	13.02734314154568	6.73810768483645	5.24610442783858
C	14.32041364698792	6.20546360260513	5.21509605029913
H	15.18279801709826	6.85036484989952	5.38668433914752
C	14.51173507382779	4.82925413126680	5.04399462758860
C	13.40593652216210	3.97613831757490	4.90154612601081
H	13.52176053950805	2.90538721121945	4.72550647274959
C	12.11322917804543	4.52557115827825	4.94234906821905
C	11.91705815262669	5.89561966445092	5.08569386131423
H	10.90535725345837	6.30128894660562	5.10560225087677
C	14.21057610060126	10.40334015604068	9.98552529463391
H	13.64053258203438	10.05838978554441	10.85844742995991
H	14.50133233428908	11.46038169625810	10.17051223985089
C	15.43414266821361	9.52720921287438	9.82773980810090
H	16.14166710604111	9.95348036165767	9.09033485592733
H	16.00361050334499	9.52068780241130	10.77597188482001
C	16.11498631155127	7.35779876017102	9.43822596760521
C	15.92380449090011	5.88037544826137	9.18650798035975
C	15.29370533660902	5.05452478156670	10.13014272154447
H	14.82001101109308	5.51877286021032	10.99487086140487
C	15.25157159057397	3.66759682911524	9.92784958004060
C	15.82032619988182	3.10475851254104	8.77542207730790
H	15.77865871392414	2.02415086524035	8.62434312299874
C	16.45297686296784	3.94007314699128	7.84471238877373
C	16.52480364695258	5.31602019094350	8.05122946578683
H	17.02889141911986	5.94720157669987	7.31947480288278
C	11.95949380059684	10.66653840179740	9.09661737070192
H	11.43473407438155	10.69443809188660	8.13312730372660
H	11.90093232301196	11.68265662255715	9.54379013177519

C	11.28966701263536	9.63990347475922	9.98316942358360
H	11.63171192678843	9.72915978101571	11.03296188108314
H	10.20410601525912	9.84580406755481	10.03393847000481
C	10.85628188291044	7.37253166689279	10.15630280469473
C	10.96038462173593	5.95034291974372	9.67035674836096
C	10.40668487286628	5.55023739294275	8.45126554286811
H	9.95056218364221	6.27913309006399	7.78118069986204
C	10.51460640262631	4.21419726549070	8.04733764040585
C	11.18663597563396	3.28198668710273	8.85209286384254
H	11.28987696195917	2.23357173868526	8.56833104418916
C	11.74636503088085	3.70132121834526	10.07163386191226
C	11.59257664553576	5.01693036576568	10.50369856060856
H	11.98002573553586	5.36034083029352	11.46227073045807
C	16.14739875693611	3.16784294440601	5.64595091121113
H	15.23341290801545	2.64930078251174	5.99269895005481
N	13.27225763389703	6.19333084497082	8.04302543200339
C	13.42496573199167	5.05519326576706	7.86317142923378
H	13.62197259557495	4.01366674819306	7.71083655738630

*

S7. References

1. Stoll, S.; Schweiger, A. *Mag. Res.* **2006**, *178*, 42.
2. *CrysAlisPro*, version 171.42.64a; Rigaku Corporation: Oxford, UK, 2022
3. Sheldrick, G. M. *SHELXT*, version 2018/2; *Acta. Crystallogr.* **2015**, *A71*, 3-8.
4. Sheldrick, G. M. *SHELXL*, version 2019/2; *Acta. Crystallogr.* **2015**, *C71*, 3-8.
5. Dolomanov, O. V.; Bourhis, L. J.; Gildea, R. J.; Howard, J. A. K.; Puschmann, H. *Olex2*, version 1.5; *J. Appl. Cryst.* **2009**, *42*, 339-341.
6. Neese, F. *Wiley Interdiscip. Rev.: Comput. Mol. Sci.* **2012**, *2,* 73.
7. Handy, N. C.; Cohen, A. J. Left-Right Correlation Energy. *Mol. Phys.* **2001**, *99* (5), 403-412.
8. Grimme, S.; Ehrlich, S.; Goerigk, L. *J. Comp. Chem.* **2011**, *32*, 1456-1465.
9. Schaefer, A.; Horn, H.; Ahlrichs, R. *J. Chem. Phys.* **1992**, *97,* 2571.
10. Weigend, F.; Ahlrichs, R. *Phys. Chem. Chem. Phys.* **2005**, *7*, 3297-3305.
